# Covalently linked ferrocene–polyoxometalate dyads for light-induced radical generation

**DOI:** 10.1039/d6sc02948e

**Published:** 2026-06-17

**Authors:** Sebastian Knoll, Heiner Schmidt, Kevin Sowa, Benjamin Dietzek-Ivanšić, Celina Titze, Stephan Kupfer, Linda Zedler, Carsten Streb

**Affiliations:** a Institute of Inorganic Chemistry I, Ulm University Albert-Einstein-Allee 11 89081 Ulm Germany; b Institute of Physical Chemistry, Friedrich Schiller University Jena Helmholtzweg 4 07743 Jena Germany stephan.kupfer@uni-jena.de; c Leibniz Institute of Photonic Technology Albert-Einstein-Straße 9 07745 Jena Germany linda.zedler@leibniz-ipht.de; d Department of Chemistry, Johannes Gutenberg University Mainz Duesbergweg 10-14 55128 Mainz Germany carsten.streb@uni-mainz.de; e Leibniz Institute of Surface Engineering Permoserstraße 15 04318 Leipzig Germany

## Abstract

The design of covalently linked photoactive donor–acceptor dyads offers major opportunities for photocatalysis and solar energy conversion. Here, we report a noble metal-free dyad obtained by covalent anchoring of a ferrocene moiety to a Dawson-type polyoxotungstate. The resulting dyad shows visible light photoinduced charge-separation and electron transfer from the ferrocene to the polyoxometalate. The separated charges can be used for the photoinduced, oxidative and reductive activation of organic peroxides to generate oxygen-based radicals. Structural and mechanistic studies using *in situ* spectroscopy, time-resolved spectroscopy and spectro-electrochemistry as well as quantum chemical calculations shed light on the underlying photoinduced reactivity. The study presents a blueprint for the design of photoactive covalent photosensitizer–polyoxometalate dyads based on earth-abundant elements.

## Abstract 2

A photoactive, covalently linked ferrocene-Dawson-polyoxometalate dyad is reported and the photophysical and photoredox properties of the dyad are studied by spectroscopic and theoretical methods.

## Graphical abstract

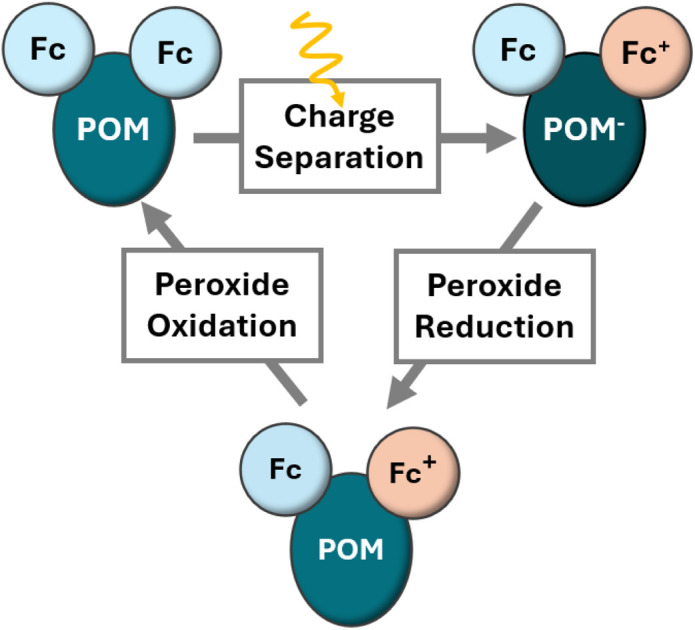

## Introduction

Covalently linked photoactive donor–acceptor molecules, so-called dyads, have received widespread attention in the fields of solar energy conversion and photocatalysis. The linkage of a photoactive donor, or photosensitizer, to a redox-active acceptor for charge-storage and subsequent charge-transfer opens a multitude of opportunities for light-induced charge separation and charge-transfer. In addition, these systems offer ultimate control over structure and reactivity by chemical modification of each component. In recent years, molecular metal oxides, so-called polyoxometalates (POMs), covalently functionalized with photosensitizers have received widespread attention as models for molecular light-harvesting systems.^1,2^ Groundbreaking research has shown that several types of POMs including Keggin-,^3–5^ Dawson-^4,6^ or Anderson-type^7–11^ clusters can be covalently functionalized with photosensitizers including metal complexes^3,4,6–12^ and organic dyes.^5,13,14^ In seminal studies, Izzet, Proust, Artero and co-workers reported the light-induced charge-accumulation and light-driven hydrogen evolution using Ir-complex functionalized Dawson-polyoxotungstates.^4,6^ The authors demonstrated that up to two electrons could be transferred to the cluster whilst maintaining structural integrity of the molecular assembly. Building on these studies, Rau, Dietzek and Streb have explored the attachment of ruthenium or iridium complexes to various POMs and observed that robust, selective and mild functionalization strategies are critical to prevent uncontrolled aggregation^15^ or even POM/photosensitizer degradation.^16,17^ In addition, the groups showed that separation of light-harvesting and catalytic turnover is possible, leading to decoupled hydrogen evolution in the dark.^18^

Most of the reported metal complex photosensitizer–POM (PS–POM) dyads utilize noble metal-based PS species such as ruthenium or iridium complexes.^3,4,6–12,18^ In contrast, very little is known about PS–POM dyads based on earth-abundant metals. Pioneering work on noble metal free PS–POM dyads was reported by Lampre, Ruhlmann and Hasenknopf, where a zinc(ii)-tetraphenyl porphyrin PS was covalently attached to a Dawson-vanadotungstate *via* a triol (TRIS) anchoring group. Detailed photophysical analyses showed reduced excited-state lifetimes and reduced emission intensity in the dyad and provided critical insights into the charge-separated states formed upon excitation.^19^ Recently, Orio, Blanchard and co-workers reported a structurally related Cu-PS-Dawson-POM dyad capable of light-induced generation of CF_3_ radicals.^20^

Building on these ideas, we were intrigued to utilize earth-abundant metals as photoactive units in PS–POM dyads. In this context, ferrocenes are a promising class of photoactive metal complexes, which combine facile synthetic access with promising photoredox activity. Seminal work by Hirsch, Echegoyen and Guldi demonstrated the covalent linkage of ferrocene-azafullerene dyads. Using emission and transient absorption (TA) spectroscopy, they observed charge-transfer (CT) from ferrocene to the azafullerene resulting in rapid formation of the charge-separated state (C_59_N^−^/Fc^+^).^21^ Also, Ziegler and Nemykin developed a ferrocene acceptor dyad by covalent linkage of two ferrocene units to the α-pyrrole positions of an aza-Bordipyrromethen (aza-BODIPY) moiety. These dyads exhibit low-energy CT bands corresponding to electron transfer from ferrocene to the aza-BODIPY core, which were further corroborated by time-dependent density functional theory (TD-DFT) simulations. The origin of these bands was confirmed through electrochemical oxidation of the ferrocene units, which led to the disappearance of the CT bands and thus verifying the chromophore role of the ferrocenes.^22^ Wu exploited ferrocene as a redox-switchable group by grafting ferrocene units onto an Anderson-polyoxometalate, thereby enabling redox-control over self-assembly.^23^ To the best of our knowledge, no photoactive ferrocene–POM dyads have been reported to-date, despite the promising nature of both components as outlined above.

Here, we report synthetic access to covalently linked ferrocene-Dawson-polyoxometalate dyads. We explore their photophysical properties and photochemical reactivity and discuss their function as one-electron radical generators. This work provides a rare glimpse into entirely noble metal-free polyoxometalate dyads with unique visible light photoredox reactivity.

## Results and discussion

### Dyad synthesis and characterization

**Fig. 1:**
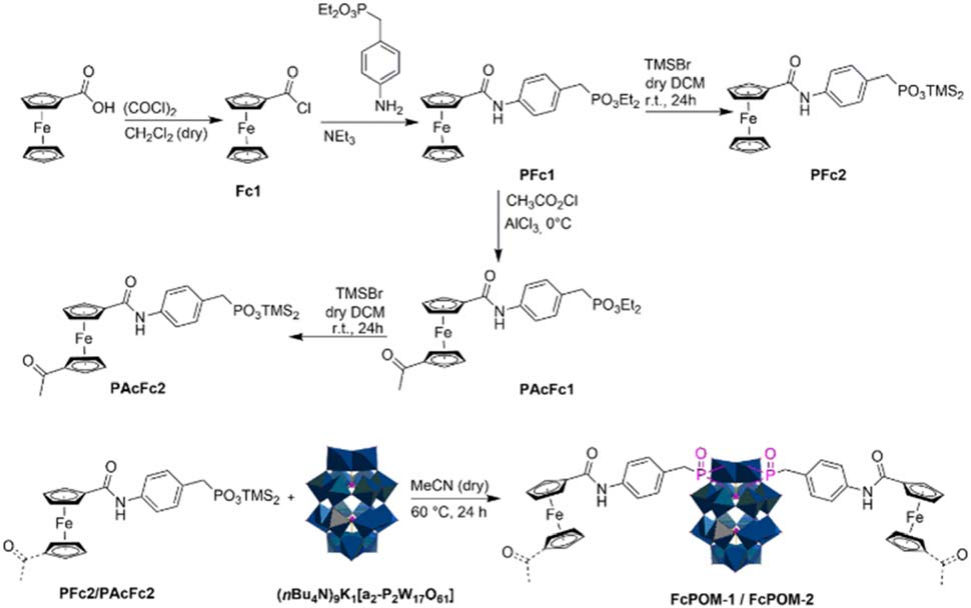
Synthesis of the ferrocene derivatives PFc2 and PAcFc2, and the Fc–POM dyads FcPOM-1 (based on PFc2) and FcPOM-2 (based on PAcFc2).

Briefly, the title compound was obtained starting from commercially available ferrocene carboxylic acid, which was converted to the corresponding ferrocene carboxylic acid chloride (Fc1) using oxalyl chloride. Fc1 reacted under alkaline conditions with *O*,*O*-diethyl *p*-aminobenzyl phosphonate, resulting in *O*,*O* diethyl(4-ferrocene-amidobenzyl) phosphonate (PFc1). Then, a Friedel–Crafts acylation (using acetyl chloride and aluminium chloride as catalyst) was used to convert PFc1 into the acylated PAcF1 (*O*,*O*-diethyl (4-(1'acetylferrocene) amidobenzyl) phosphonate). PFc1 and PAcFc1 were then reacted with trimethyl bromosilane to obtain activated phosphonates, resulting in PFc2 and PAcFc2. These species were then reacted with the lacunary Dawson derivative (*n*Bu_4_N)_9_K_1_[α_2_-P_2_W_17_O_61_] under inert, water-free conditions in acetonitrile (MeCN) to give the ferrocene-functionalized PS–POM dyads FcPOM-1 and FcPOM-2 (Fig. 1). For synthetic and analytical details, see SI, Section 2.

### Photochemical and electrochemical dyad characterization

**Fig. 2:**
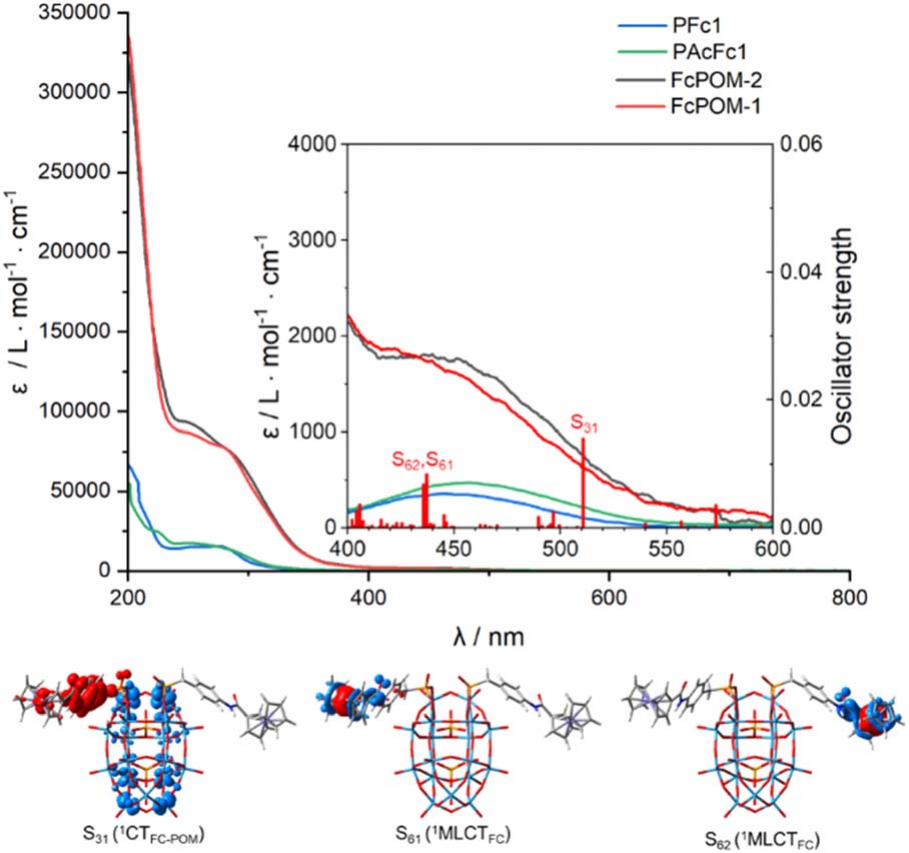
Top: experimental and calculated UV-vis-NIR absorption spectra of the ferrocene precursors PFc1 (blue) and PAcFc1 (green), as well as the Fc–POM dyads FcPOM-1 (red) and FcPOM-2 (black). Inset: magnified view of the ferrocene-centered transition region (*λ* = 400–600 nm). TD-DFT-simulated transitions are provided for FcPOM-1 (red). Solvent: water-free de-aerated MeCN. Bottom: charge-density difference plots indicating the nature of the key electronic transitions; charge-transfer occurs from red to blue.

UV-vis-NIR absorption spectroscopy of the ferrocene ligands PFc1/PAcFc1 revealed characteristic, low intensity bands assigned to electron transitions of the complex coordination environment from the d(Fe) orbitals and the π-system of the cyclopentadienyl ligands.^24^ For the non-acetylated PFc1, these metal-to-ligand charge-transfer (MLCT) transitions were observed at *λ* = 446 nm (*ε* = 358 L mol^−1^ cm^−1^). For the acylated PAcFc1, these transitions were observed with 456 nm (*ε* = 471 L mol^−1^ cm^−1^) at a slightly lower excitation energy. Also, cyclopentadienyl-centered π–π* transitions were observed for both compounds in the range of 200–300 nm (Fig. 2). For the Fc–POM dyads FcPOM-1 and FcPOM-2, the characteristic ferrocene-based absorptions were likewise detected; however, the UV region is predominantly governed by the intense POM-centered O → W ligand-to-metal charge-transfer (LMCT) bands below *ca.* 350 nm, which partially overlap with the ferrocene π–π* and d–d transitions (Fig. 2). The corresponding molar extinction coefficients are in good agreement with values previously reported for related organo-functionalized Dawson POMs.^25^ The assignment of the electronic transitions underlying the electronic absorption features was further rationalized for FcPOM-1 by means of quantum chemical simulations. In synergy with the experimental findings, time-dependent density functional theory (TD-DFT, see SI for details regarding the computational setup) predicts several weakly dipole-allowed singlet ^1^MLCT_FC_ transitions, *i.e.* into S_61_ and S_62_, at 437 and 436 nm (Fig. 2) as well as into S_90_ and S_94_ at 406 and 404 nm, respectively. Interestingly, TD-DFT predicts in addition to these Fc-centered transitions several low-lying CT transitions from the Fc units and amid-linkers toward the POM in the visibly region between 957 and 511 nm, see *e.g.* S_31_ (at 511 nm) in Fig. 2. Likewise, these ^1^CT_Fc–POM_ and ^1^CT_L–POM_ transitions are merely weakly dipole-allowed, yet these transitions indicate the desired light-driven CT and charge-separation from the Fc redox moieties to the Dawson POM. Finally, POM-centered LMCT transitions are predicted, *e.g.* into S_174_, S_176_, S_198_, S_212_ and S_262_ between 371 and 347 nm. For further details, see SI, Fig. S43, Tables S10 and S11 as well as the respective repository *via* ref. 26.

### Electrochemistry

**Fig. 3:**
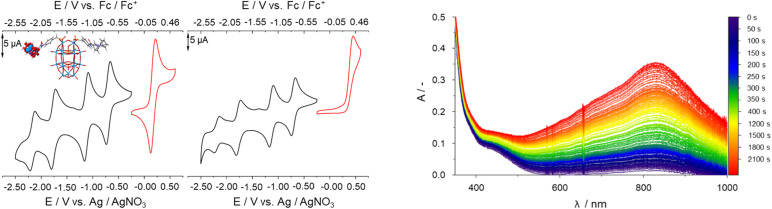
Left: cyclic voltammetry of the Fc–POM dyads FcPOM-1 (left) and FcPOM-2 (right), showing the tungsten-based W^VI/V^ redox couples (black), and the iron-based Fe^III/II^ redox couples (red). Spin density of the singly oxidized doublet species of Fc-POM-1 reveals a Fc-centered oxidation event. Conditions: solvent: de-aerated, water-free *N*,*N*-dimethyl formamide (0.1 M *n*Bu_4_NPF_6_ supporting electrolyte), [FcPOM-1, FcPOM-2] = 1 mM. Right: *in situ* UV-vis-NIR spectroscopy of the photoreduction of the Fc–POM dyad FcPOM-1 (0.1 mM) in water-free, de-aerated MeCN in the presence of the sacrificial electron donor triethylamine (0.5 M) by LED irradiation, *λ*_max_ = 470 nm. The emergence of the characteristic IVCT band (*λ*_max_ = 840 nm)^18^ indicates one-electron reduction of the Fc–POM dyads.

Electrochemical analysis of FcPOM-1 and FcPOM-2 (Fig. 3) was performed in a standard three-electrode setup (working electrode: glassy carbon, counter electrode: platinum wire, pseudo-reference electrode: (Ag/AgNO_3_) in water-free, de-aerated *N*,*N*-dimethyl formamide containing 0.1 *n*Bu_4_NPF_6_ as supporting electrolyte (details see SI, Section 2). All potentials are referenced against the ferrocene/ferrocenium (Fc^+^/Fc) redox couple used as internal standard. Cyclic voltammetry shows that both species feature four reversible tungsten-based^27^ redox events at half-wave potentials (*E*_1/2_) of approx. −0.8 V, −1.2 V, −1.8 V, and −2.2 V (Fig. 3). FcPOM-1 exhibits a reversible, formally two-electron redox wave (*E*_1/2_ = 0.13 V), which is assigned to two simultaneous one-electron Fe^III/II^ transitions. The localization of the first oxidation event was further investigated by density functional theory. Full structural equilibration of the singly reduced doublet species (^2^[FcPOM-1]) reveals in agreement with the electrochemical results a Fc-based oxidation and a redox potential for the one-electron process of 0.26 V against the previously investigated Fc^+^/Fc couple.^28^ In contrast, FcPOM-2 shows a non-reversible formal two-electron oxidation (*E*_ox_ = 0.41 V). This behavior suggests an electrochemical-chemical process, where ferrocene oxidation is followed by a subsequent chemical reaction due to the instability of ferrocenium cations featuring electron withdrawing groups.^29^ The anodic behaviour of the ferrocene redox potentials is attributed to the substitution pattern. The electron-withdrawing amide group present in both derivatives, along with the acetyl group in FcPOM-2, could reduce the energy of the highest occupied molecular orbitals (HOMO), which may account for the positive shift of the redox potentials relative to the Fc^+^/Fc ref. 30.

### Photophysical studies and light-induced charge-accumulation

Initial steady-state irradiation experiments of the Fc–POM dyads were performed to study the light-induced charge-transfer between ferrocene and POM units. To this end, the FcPOM-1 dyad was dissolved in water-free, de-aerated MeCN in the presence of the electron/proton donor triethyl amine (TEA) and irradiated using LED light sources. Irradiation (at *λ*_exc_ = 400 nm or *λ*_exc_ = 470 nm) resulted in the formation of a broad intervalence charge-transfer (IVCT) band (between *ca.* 500 nm 1100 nm, *λ*_max_*ca.* 840 nm), see Fig. 3, bottom and SI, Section 3.2). This observation is characteristic for the formation of a one-electron reduced Dawson polyoxotungstate species.^4,6,18,31^

Photoreduction of FcPOM-1 was compared with two non-covalent intermolecular reference systems (PFc1 + [P_2_W_17_O_57_(PO_3_C_7_H_7_)_2_]^6−^; PFc1 + [P_2_W_17_O_61_]^10−^),^32,33^ and faster photoreduction kinetics and higher photonic efficiencies (PE) were observed for the covalent dyad compared with two intermolecular reference systems (SI, Section 3.2).

To gain mechanistic understanding of the photochemical and photophysical processes occurring under irradiation, we performed femtosecond transient absorption (fs-TA) spectroscopy as well as UV-vis-NIR and resonance Raman (rR) spectro-electrochemistry (SEC) in combination with TD-DFT simulations. Our goal was to explore charge-separation and charge transfer processes when irradiating into the ferrocene absorption bands. To probe the individual charge transfer steps between Fc and POM, SEC was used, where the one-electron reduced state of Fc–POM was accessed by electrochemical reduction, and spectroscopic probing of this species was performed using UV-vis-NIR and rR-SEC. The spectro-electrochemical studies were performed in MeCN.

**Fig. 4:**
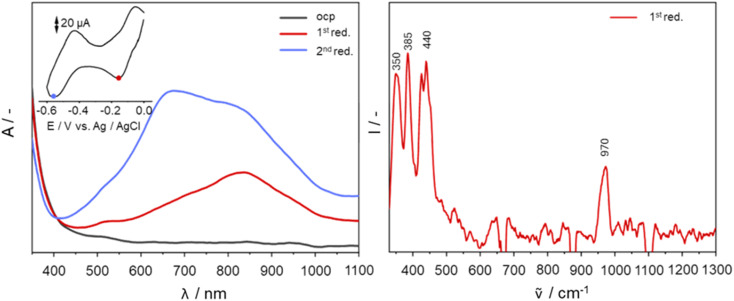
Left: *in situ* UV-vis-NIR spectro-electrochemical analysis of the one- and two-electron reduction of FcPOM-1, showing the emergence of characteristic IVCT absorption bands of the one-electron reduced (840 nm) or the two-electron reduced POM cluster (650 nm). Inset left: CV of FcPOM-1 in de-aerated MeCN containing 0.1 M *n*Bu_4_NBF_4_. The potentials applied during acquisition of the UV-vis-NIR spectra are indicated by a red or blue dot. Conditions: scan rate 100 mV s^−1^, electrodes: glassy carbon (working), Pt (counter), Ag/AgCl (pseudo-reference). Right: resonance Raman spectrum of the one-electron reduced FcPOM-1 in MeCN containing 0.1 M *n*Bu_4_NBF_4_, *λ*_exc_ = 643 nm. Modes assigned to the one-electron reduced POM cluster are labeled.

SEC analyses in a miniaturized cell show that the one- and two-electron reductions of FcPOM-1 detected by cyclic voltammetry (CV) (Fig. 3, left inset) can also be observed under SEC conditions. Correspondingly, UV-vis-NIR absorption maxima at *λ*_max_ = 840 nm (first reduction) and *λ*_max_ = 650 nm (second reduction) are observed (Fig. 4, left).^4,6,18,31^ Next, we used rR-SEC and probed the IVCT absorption region of the one-electron reduced FcPOM-1 dyad (*λ*_exc_ = 643 nm, Fig. 4, right). This study showed the characteristic resonance Raman features associated with a one-electron reduced Dawson polyoxotungstate, specifically W–O–W asymmetric stretching modes (970 cm^−1^) as well as further W–O–W bending and wagging modes below 450 cm^−1^.^34,35^ The results of these SEC analyses show, that the one- and two-electron reduced Fc–POM species are stable on the experimental timescales, that a clear differentiation between one and two-electron reduction is possible based on the IVCT band maxima, and that the electrons added are delocalized across several tungsten centers of the POM cluster.

**Fig. 5:**
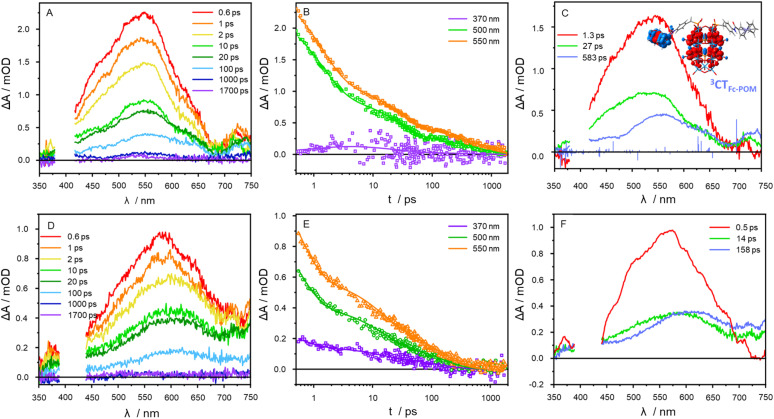
Transient absorption spectroscopic data for FcPOM-1 (A–C) and FcPOM-2 (D–F). (A and D): transient absorption spectra at selected delay times; (B and E): transient kinetics at selected wavelengths; (C and F): spectral changes associated with each kinetic process (decay associated spectra, DAS). The TD-DFT simulated spectra signature of the triplet charge-separated species of FcPOM-1 is shown along with the respective spin density as inset (C). Conditions: solvent: water-free, de-aerated MeCN, pump wavelength: 403 nm.

To investigate the CT dynamics upon excitation of the ferrocene moiety (at *λ*_exc_ = 403 nm), fs-TA spectroscopy was performed. In the following we will discuss and compare the photoinduced electron transfer in FcPOM-1 and FcPOM-2, as the peripheral acetyl group is known to affect the electronic structure and (photo)redox properties of ferrocene.^36^ In both Fc–POM dyads, excitation of the Fc unit results in a broad excited state absorption (ESA) spanning from 400 to 750 nm (Fig. 5A and D). The ESA maximum is marginally red-shifted for FcPOM-2 compared with the non-acetylated FcPOM-1, indicating that Fc-to-POM electron transfer is energetically favored for FcPOM-2 compared with FcPOM-1. This is in line with the significantly more positive Fc redox potential of FcPOM-2 (see Fig. 3). The faster charge recombination observed in FcPOM-2 further supports this and is reflected in the kinetic traces plotted for selected probe wavelengths (Fig. 5B and D).

Global analysis of the TA spectra was performed using the software package KiMoPack22 (ref. 37) and reveals spectral changes associated with tree distinct kinetic processes (Fig. 5C and F). The respective time-constants are: Fc-POM-1: *τ*_1_ = 1.3 ps, *τ*_2_ = 27 ps, *τ*_3_ = 583 ps; FcPOM-2: *τ*_1_ = 0.5 ps, *τ*_2_ = 14 ps, *τ*_3_ = 158 ps. These three kinetic processes are consistent with the two excited states invoked, *i.e.*, the Fc-localized excited state and the charge-separated (Fc^˙+^–POM^˙−^) state: *τ*_1_ reflects decay of the Fc-localized excited state by Fc-to-POM forward electron transfer.^38–40^ Notably, the early-time transient features show strong spectral resemblance to those of the reference compound PFc1 (see SI, Fig. S39), suggesting contribution from a ferrocene-centered excited state. However, given the presence of low-lying charge transfer states indicated by TD-DFT, we cannot exclude that locally excited and charge-transfer states coexist on ultrafast timescales (Fig. 5C and F, red; for reference see TA spectra of the ferrocene PFc1, SI, Fig. S40).

*τ*_2_ corresponds to the evolution of the initially formed charge-separated state toward the relaxed, thermally and structurally equilibrated charge-separated state, *i.e.*, formation of a Fc^˙+^–POM^˙−^ charge-separated transient state (Fig. 5C and F, green). This species has a much higher absorbance ratio Abs (750 nm)/Abs (550 nm) compared with the first transient species. *τ*_3_ Represents the decay of the relaxed charge-separated state by back electron transfer. The lifetime of the charge-separated state is much higher for FcPOM-1 (583 ps) compared with FcPOM-2 (158 ps) since the back electron transfer from the reduced POM to the excited ferrocenium is faster in FcPOM-2 than in FcPOM-1.

Further evidence that Fc–POM linkage results in efficient Fc-to-POM electron transfer and the formation of a charge-separated state is provided by comparing the TA spectra of FcPOM-1 with those of the native ferrocene PFc1 (SI, Fig. S40). While the ESA in the TA spectra of FcPOM-1 extends over a range of 400 to 750 nm, the ESA of PFc1 is spanning from 400 to 650 nm. The broadening of the ESA in the dyad is caused by the additional absorption arising from the reduced POM. In addition, the PFc1 photosensitizer has a significantly shorter lifetime in comparison to the FcPOM-1 dyad and relaxes back to the ground state in less than 50 ps.

Additional insights into the excited state relaxation processes and the formation of the charge-separated species are provided by means of computational modelling. To this end, the lowest energy triplet state of FcPOM-1 was structurally relaxed at the unrestricted DFT level of theory; notably, intersystem crossing typically occurs for Fc-based systems within the sub picosecond timeframe till few picoseconds.^38–40^ The spin density as shown in Fig. 5C (Table S13) reveals a triplet ^3^CT_Fc–POM_ species which features one (photo)oxidized Fc and a singly (photo)reduced Dawson POM. Relative to the relaxed singlet ground-state this ^3^[FcPOM-1] species is predicted at an energy level of merely 0.89 eV. The measured lifetime of 583 ps reflects on one hand the large spatial separation of the electron–hole pair (decoupling and prolonging the lifetime) whereas the comparatively small T_1_–S_0_ energy gap of 0.41 eV (typically associated with decreasing lifetime) is predicted within ^3^[FcPOM-1]. In addition, the TA spectrum of FcPOM-1 was simulated, while the ESA features were modelled by means of the spin and dipole-allowed triplet-to-triplet transitions within ^3^[FcPOM-1] while ground state bleach (GSB) was accounted for by the respective singlet-to-singlet transitions within the Franck–Condon point; see SI for details. This way, the electronic transitions underlying the TA spectra of FcPOM-1 could be assigned based on the charge-separated species, *i.e.* at comparably long delay times and correlated to the signature of the decay associated spectra (DAS) at 583 ps, see Fig. 5C and S43. The computational modelling allows us to associate the ESA at 750 nm with an excitation into the POM-centered ^3^LMCT_POM_ state T_16_ at 702 nm. In a similar fashion the broad ESA feature ranging from 400 to 650 nm is associated with several ^3^LMCT_POM_ transition, *e.g.* into T_25_ and T_28_ (at 634 and 627 nm) as well as transitions associated with charge-recombination from the amide linker to the respective photooxidized ferrocene unit (T_110_ at 463 nm). Further information regarding the TD-DFT simulated signature and the electronic transitions involved in the ESA are collected in Tables S12 and S13 of the SI.

These data indicate that covalent linkage between ferrocene and POM results in significant ferrocene–POM electronic coupling. The observations can be summarized as follows: irradiation into the ferrocene absorption band (403 nm) results in a fast fc-to-POM electron transfer, resulting in transient formation of an oxidized ferrocenium cation and a one-electron reduced POM species. Charge-separation and charge-recombination are faster for FcPOM-2 compared with FcPOM-1. This is rationalized by the presence of the electron withdrawing acetyl group, which destabilizes the charge-separated state and results in faster electron transfer kinetics.

### Ferrocenium cation generation

**Fig. 6:**
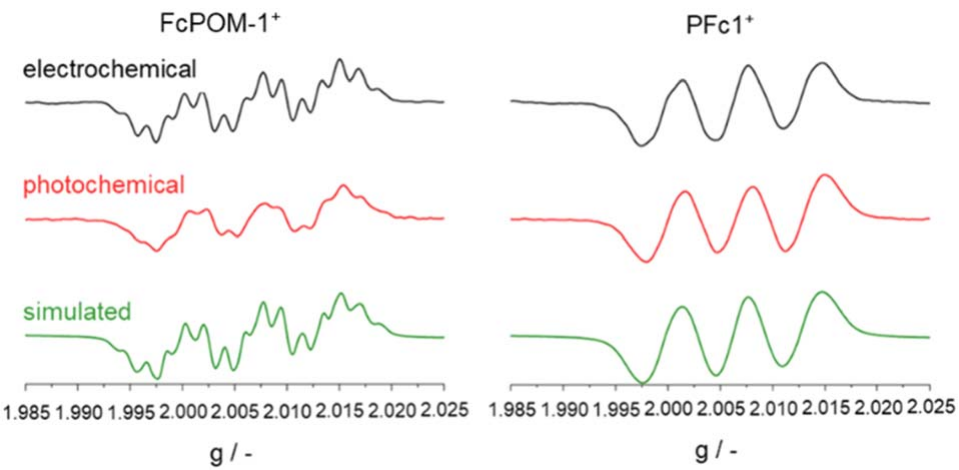
EPR spectroscopic analysis of the oxidized ferrocenium species FcPOM-1^+^ (left) and PFc1^+^ (right); the species were trapped using nitrosobenzene spin traps.^41^ Top: electrochemical oxidation at *E*_ox_ = 550 mV *vs.* Fc/Fc^+^) (*t*_electrolysis_ = 90 min); center: photochemical oxidation with *λ*_exc_ = 470 nm (*t*_irrad_ = 30 min), bottom: simulated EPR spectra (simulation details see SI, Section 3.3).

Next, we set out to probe whether trapping of transient, oxidized ferrocenium in the Fc–POM dyads is possible. This work was inspired by a report from Heinze and co-workers, who demonstrated the presence of ferrocenium using nitrosobenzene as a spin trap.^41^ Their method involved reaction of ferrocenium with nitrosobenzene, resulting in generation of metastable carbon-centered radicals which were subsequently detected by electron paramagnetic resonance (EPR) spectroscopy.^41^ Following this procedure, both the ferrocene ligand PFc1 and the dyad FcPOM-1 were subjected to bulk electrolysis in MeCN at *E*_ox_ = 550 mV *vs.* Fc^+^/Fc in the presence of the nitrosobenzene spin trap (for details see SI, Section 3.3). EPR spectroscopic analysis of the resulting solution showed that both PFc1 and FcPOM-1 feature nearly identical EPR signals which are consistent with the trapped radical signals reported by Heinze and colleagues (Fig. 6).^41^ Notably, FcPOM-1 featured a hyperfine structure (Fig. 6, left), which was not observed for PFc1 (Fig. 6, right). A similar EPR signal was also obtained by *in situ* photo-oxidation of the FcPOM-1 or PFc1 by LED irradiation (*λ*_exc_ = 470 nm) in the presence of the nitrosobenzene spin trap (Fig. 6), highlighting that data from electrochemical and photochemical oxidation are consistent. Note that no reaction was observed when the samples were not irradiated, thereby providing evidence for the photochemical nature of the process in both, FcPOM-1 and PFc1.

### Radical generation from organic peroxides

To study the potential use of FcPOM dyads for radical generation, we first explored the photochemical reactivity of FcPOM towards O_2_: irradiation of FcPOM-2 in oxygenated MeCN in the absence of a sacrificial electron donor (LED irradiation, *λ*_exc_ = 470 nm, *t*_irrad_ = 24 h) resulted in a color change from orange to deep brown. The resulting species was precipitated by addition of *n*Bu_4_NCl and water, resulting in the isolation of a brown powder. ATR-FT-IR spectroscopy was used to examine the FcPOM-2 sample before and after irradiation (see SI, Fig. S24). Analysis of the IR spectra showed virtually identical vibrational signals assigned to the intact FcPOM-2 cluster, with only minor signal changes in the fingerprint region. Most notably, the carbonyl stretching signal at 1661 cm^−1^ exhibited peak broadening, and a new signal at 1710 cm^−1^ was observed. The difference in wavenumber (49 cm^−1^) is consistent with the formation of the ferrocenium species and agrees well with literature values for similar compounds.^42^ This data suggests that under the given reaction conditions, photooxidation of FcPOM-2 was observed, possibly by excited-state electron transfer from the dyad to molecular oxygen and formation of peroxide species.

**Fig. 7:**
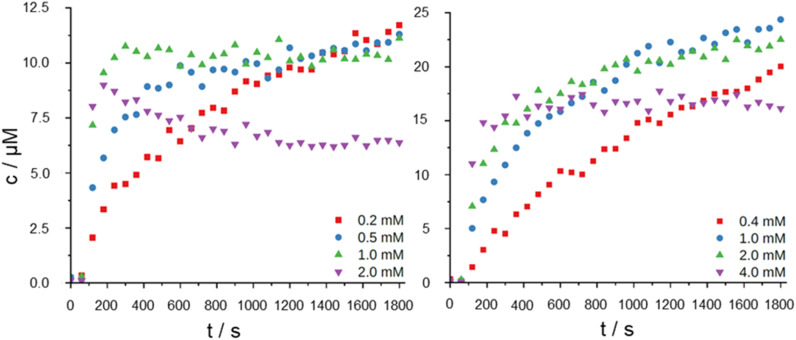
Photoinduced organic radical generation by (left) FcPOM1 and (right) PFc1. Left: variation of FcPOM-1 concentrations, constant *t*BuOOH concentration (1.25 mM). Right: variation of PFc1 concentrations, constant *t*BuOOH concentration (1.25 mM).

**Fig. 8:**
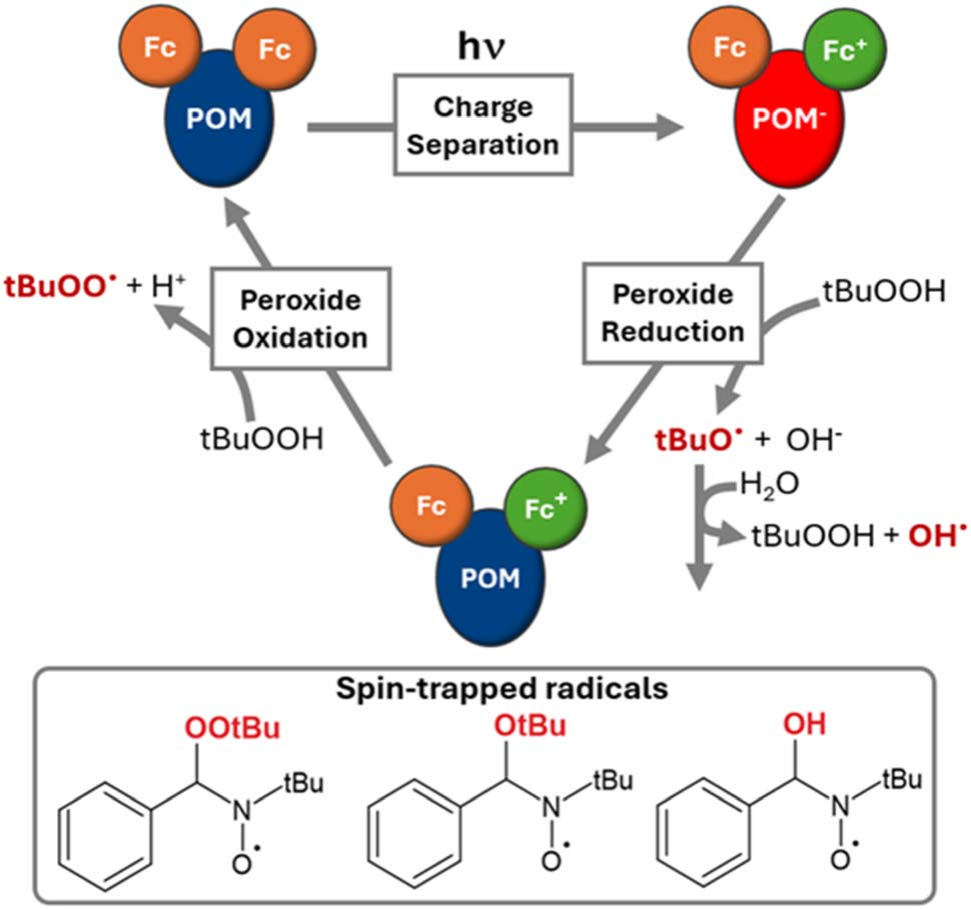
Top: simplified scheme of the photoinduced oxidative and reductive generation of organic oxygen-centered radicals starting from *t*BuOOH. Bottom: types of spin-trapped radicals observed by *in situ* EPR experiments. For details see SI, Sections 3.3–3.6.

Based on these initial results, we probed the reactivity of FcPOM-1 and the PFc1 reference with organic peroxides (using *tert*-butylhydroperoxide, *t*BuOOH as model), as a potential path for the light-driven generation of organic radicals.^20^ To this end, acetonitrile solutions containing the respective ferrocene compound (FcPOM-1 or the PFc1 reference) were irradiated inside the EPR spectrometer using LED irradiation (*λ*_exc_ = 470 nm, see SI Section 5), to enable the detection of organic radicals by *in situ* EPR spectroscopy. Radical species were trapped using the nitrone spin trap PBN (*N-tert*-butyl-α-phenyl nitrone).^43^ We observe that both, FcPOM-1 and PFc1 can initiate the light-induced generation of organic radicals by activation of organic peroxides. At identical ferrocene concentrations, both species show comparable performance in terms of amounts of radicals produced per time (Fig. 7, and SI, Sections 3.4–3.6). However, the covalent Fc–POM dyads enable reactivity that neither component delivers alone: excitation by a single VIS photon generates both an oxidant (Fc^+^), and a reductant (POM^−^), which in turn enables both oxidative and reductive activation of suitable substrates (here: *t*BuOOH, see Fig. 8). This principle can in future be harnessed to couple two redox–half reactions and develop molecular systems for two productive reactivity manifolds. In addition, covalent linkage of Fc and POM extends the charge-separated lifetime by more than one order of magnitude and threefold increase in electron transfer photonic efficiency.

Increasing the dyad concentration did not result in an increase in trapped radical concentration; instead, we observed that the rate of radical formation was influenced: for both, FcPOM-1 and PFc1, the radical formation rate increases with higher concentration of the photoactive compound. The radical formation rate increases steadily at low concentrations of FcPOM-1 or PFc1, and reaches a plateau, while at higher concentrations, we even observe a rate decrease (Fig. 7). This concentration-dependent behaviour indicates the presence of follow-up radical reactions and therefore, decrease of radical concentration in solution. For brevity, a more detailed kinetic analysis including analysis of relative reaction rates and impact of water in the reaction solution is provided in the SI, Sections 3.5 and 3.6.

Based on these findings, we propose a light-induced radical generation and trapping mechanism which involves excitation leading to fast charge separation (as shown by transient absorption spectroscopy, see above), producing the oxidized ferrocenium (Fc^+^) and a one-electron-reduced cluster. Based on analysis of the organic radicals formed (Fig. 8 and SI, Sections 3.3 and 3.4), two simultaneous activation and radical generation pathways are plausible, based on the redox-amphoteric nature of *t*BuOOH: (a) the organic hydroperoxide *t*BuOOH can be reductively activated by electron transfer from the POM cluster, resulting in formation of an alkoxy radical (*t*BuO^˙^) and a hydroxide ion (Fig. 8). Due to the redox-amphoteric nature of peroxides, the *t*BuOOH can also be oxidatively activated by the ferrocenium unit, generating a peroxyl radical (*t*BuOO^˙^) and a proton. In our studies, both radical types were trapped by PBN, forming PBN-OO*t*Bu^˙^ and PBN-O*t*Bu^˙^ adducts (Fig. 8 and SI, Section 3.4). In addition, in the presence of water in the reaction mixture, *t*BuO^˙^ radicals can trigger the formation of hydroxyl radicals (OH^˙^) which are also observed experimentally (Fig. 8 and SI, Sections 3.4–3.6). In comparison to the cluster, the pure ligand might not react from a charge-separated state, but instead from the excited state as described in the photophysics section. In sum, these observations demonstrate that FcPOM-1 can be used as a photoactive initiator for the oxidative and reductive activation of organic peroxides, resulting in organic radical generation as summarized in Fig. 8.

## Conclusions

In summary, we report a rare example of a covalently linked, noble metal-free complex-photosensitizer–polyoxometalate dyad with visible light photoactivity. The study shows that covalent linkage of ferrocene derivatives to Dawson-polyoxometalates leads to covalent dyads capable of visible light absorption, charge-separation and light-induced oxidative or reductive radical generation. Covalent linkage of both species generates a spatially separated oxidant/reductant pair (ferrocenium and reduced POM) that enables simultaneous oxidative and reductive activation, *i.e.* reactivity not accessible to the isolated ferrocene. This is an unusual and rarely reported reactivity which could open new avenues for the development of selective, photoinitiated organic radical reactions. Detailed time-resolved photophysical and (spectro)electrochemical studies as well as complimentary TD-DFT calculations shed light on the properties and reactivity of the dyad, while initial detailed EPR-spectroscopic analyses demonstrate the mode of action of the dyads for organic peroxide activation and organo-radical generation. In future, this type of compound could be utilized for selective radical generation and radical-based synthetic chemistry.

## Author contributions

S. Knoll: conceptualization, investigation, data curation, methodology, validation, visualization, writing – original draft, writing – review and editing; H. Schmidt: investigation, data curation, methodology; K. Sowa: investigation, validation, writing – original draft; B. Dietzek-Ivanšić: funding acquisition, supervision, project administration, writing – review and editing; C. Titze: investigation, data curation, methodology; S. Kupfer: investigation, data curation, methodology, funding acquisition, supervision, project administration, writing – review and editing; L. Zedler: investigation, data curation, methodology, funding acquisition, supervision, project administration, writing – review and editing; C. Streb: conceptualization, funding acquisition, supervision, project administration, writing – original draft, writing – review and editing.

## Conflicts of interest

There are no conflicts to declare.

## Supplementary Material

SC-OLF-D6SC02948E-s001

## Data Availability

The data supporting this article have been included as part of the supplementary information (SI). In addition, all computational data are available at zenodo.org at https://doi.org/10.5281/ZENODO.18835408. Supplementary information: experimental and theoretical supplementary data available. See DOI: https://doi.org/10.1039/d6sc02948e.
