# Supplementary material for: Covalently linked ferrocene–polyoxometalate dyads for light-induced radical generation
Source: Chem Sci. 2026 Jun 17. Online ahead of print. doi: 10.1039/d6sc02948e (PMC13307311; doi:10.1039/d6sc02948e)
Supplement: SC-OLF-D6SC02948E-s001 [file SC-OLF-D6SC02948E-s001.pdf]

Supplementary Information (SI) for Chemical Science.
This journal is © The Royal Society of Chemistry 2026

Electronic Supplementary Information

Covalently linked ferrocene-polyoxometalate dyads for light-induced radical generation

Sebastian Knoll,[a] Heiner Schmidt,[b,c] Kevin Sowa,[d] Benjamin Dietzek-Ivansic,[b,c,e] Celina Titze,[b] Stephan Kupfer,*[b] Linda Zedler,*[b,c] Carsten Streb*[a,d]

[a]Institute of Inorganic Chemistry I, Ulm University, Albert-Einstein-Allee 11, 89081 Ulm, Germany
[b]Institute of Physical Chemistry, Friedrich Schiller University Jena, Helmholtzweg 4, 07743 Jena, Germany
[c]Leibniz Institute of Photonic Technology, Albert-Einstein-Straße 9, 07745 Jena
[d]Department of Chemistry, Johannes Gutenberg University Mainz, Duesbergweg 10-14, 55128 Mainz, Germany
[e]Leibniz Institute of Surface Engineering, Permoserstraße 15, 04318 Leipzig, Germany

E-mail: stephan.kupfer@uni-jena.de; linda.zedler@leibniz-ipht.de; carsten.streb@uni-mainz.de

1 Experimental Section 2
2 Synthetic Section 4
3 Characterization 16
4 Transient absorption spectroscopy 37
5 EPR LED Mount 38
6 Quantum chemistry 40
7 References 45

1 Experimental Section

1.1 Reagents and Methods

All reagents and solvents were purchased from commercial suppliers and were used without further purification. Solvents for UV-Vis absorption- and emission spectroscopic measurements were at least HPLC grade. Solvents, used for cyclic voltammetry were HPLC grade, water free solvents, all other solvents were high purity or at least HPLC grade solvents.

1.2 Cyclic voltammetry (CV)

CVs were performed on a CHI720 potentiostat using a standard three electrode setup in *N,N*-dimethyl formamide: Ag/$AgNO_3$ (in acetonitrile 0.01 M / 0.1 M $(nBu_4N)PF_6$) was used as reference electrode, a platinum wire as counter electrode and d = 3 mm glassy carbon as working electrode. All solutions were purged with argon and $(nBu_4N)PF_6$ was added as supporting electrolyte (0.1 M). All redox events were given as halve-wave potential $E_{1/2.}$

1.3 UV-Vis absorption spectroscopy

UV-Vis absorption spectroscopy was performed on a V-760 JASCO UV-VIS-NIR spectrophotometer. Gas-tight quartz glass cuvettes (d = 10.0 mm) were used for optical measurements. Time-dependent (second timescale) UV-Vis spectra were recorded on a single channel SPECORD S 600 (AnalytikJena). Quartz cuvettes with a 10 mm optical path length were used.

1.4 ^{1}H, ^{13}C and ^{31}P-NMR spectroscopy

^{1}H, ^{13}C and ^{31}P-NMR spectra were recorded at ambient temperature, unless otherwise stated, with a Bruker Avance 400 MHz, Bruker Avance Neo 400 MHz or Bruker Avance Neo 600 MHz spectrometer. All spectra were referenced to the corresponding solvent residual signal.

1.5 ATR-FT Infrared spectroscopy

ATR-FT-IR spectroscopy was performed on a Bruker Tensor 27 FT-IR spectrophotometer including a Platinum ATR unit. Signals are given as wave numbers in cm^{-1}.

1.6 Electron paramagnetic resonance (EPR)spectroscopy

EPR measurements were performed on a Magnettech MS5000 (now Bruker ESR5000). All samples were measured at constant temperatures of 25 ◦C using a 50 L capillary. The magnetic field was calibrated versus a Mn^{2+} / ZnS sample. Data evaluation was performed by a Python script. Initial baseline correction was done by a fifth polynomial, followed by the first integration. The integral, representing the microwave absorption signal was baseline corrected with drPLS (doubly reweighted penalized least squares) and integrated again, resulting in the double integral or area under the microwave absorption signal. drPLS-settings: $\lambda = 1 \cdot 10^{12}$; ratio = 0.005; $\eta = 0.5$; Iterations = 200. A TEMPOL (ESR grade) calibration was performed in acetonitrile (HPLC grade), using the same EPR settings as

used for the measurements.

1.7 Transient absorption (TA) spectroscopy

The femtosecond-TA data were acquired on a custom-built experimental setup. A similar experimental apparatus has been described in detail previously.[1] Briefly, a regenerative Ti:sapphire amplifier (Astrella, Coherent, USA) has been used as pump laser delivering pulses of 5 mJ pulse energy at 1 kHz pulse repetition rate. The output of the laser is then divided by a beam splitter. The first part is focused on a rotating CaF_2 plate to generate a broadband white light supercontinuum beam covering λ = 300 to 700 nm. This broadband pulse is subsequently split into two parts, which are used as reference and probe pulse. A second fraction of the Ti:Sa output is used to pump an optical parametric amplifier (TOPAS prime, Light conversion, Lithuania) for generating pulses of about 100 fs pulse duration and 2.5 mm^2 beam diameter. The output of the amplifier is tunable across the UV-Vis-NIR spectral range. For TA experiments, the pump pulses at 500 nm are attenuated to a pulse power of approximately 8 W. The repetition rate of the pump pulses is then reduced to 500 Hz by a mechanical chopper. A Berek compensator and a polarizer are used to adjust the polarization difference of pump and the white light probe pulse to the magic angle of 54.7°. The probe pulse is focused into a quartz cuvette of 1 mm path length by a concave mirror of 500 mm focal length. The spectra of probe and reference pulses are acquired by a 150 mm focal length Czerny-Turner spectrograph (SP2150, Princeton Instruments) equipped with a two CCD arrays (Pascher Instruments AB, Sweden). Since strong contributions of coherent artefact signals[3] are observed within the 300 fs at temporal overlap of pump and probe pulse, this temporal pulse overlap range of ±150 fs is removed from the data analysis procedure, because the multiexponential fitting algorithms cannot be applied. The TA data analysis of the remaining data includes a spectral preprocessing to correct for the chirp of the broadband pulse. Afterwards, a sum of exponential functions is fitted to the data using a least squares regression analysis for optimization using two different software tools (Pascher instruments AB or python tool KiMoPack).[4] The amplitudes of the exponential fitting correspond to the decay associated spectra (DAS). For the investigation of the primary photoinduced processes, the donor-acceptor dyads were dissolved in anhydrous acetonitrile (OD (500 nm) = 0.3 in a cell with 1 mm path length or concentrations of 10^{-4} mol · L^{-1}). UV-Vis spectra before and after TA measurement were conducted to confirm stability of the sample.

1.8 UV-Vis absorption- and resonance Raman spectro-electrochemistry

UV-Vis-, resonance Raman, spectro-electrochemistry and electrochemical measurements were performed using a three-electrode thin-layer spectro-electrochemical cell with a path length of 1 mm (Hellma, Bioanalytical Systems, USA). A three-electrode arrangement using a Pt counter electrode, an Ag/AgCl pseudo-reference electrode and a glassy carbon working electrode was utilized. Cyclic voltammetry and potential controlled monitoring were performed using a VersaSTAT 3 (Princeton Applied Research) potentiostat. UV-Vis and resonance Raman spectra were recorded immediately after applying the reduction potential to track the changes that occur during this process. UV-Vis spectra were collected in transmission mode by using a coupled UV-Vis absorption spectrometer (Avantes, AvaSpec-ULS2048XL), equipped with a deuterium-halogen light source (AvaLight DH-S-BAL). For preparing the

analyte solutions, first a 0.1 M electrolyte solution of tetra-n-butylammonium hexafluoroborate nBu_4NBF_4, Sigma-Aldrich, ≥ 99.0%) in anhydrous acetonitrile (Sigma-Aldrich) was prepared. Then the Ferrocene POM dyad was dissolved in this solution. The solutions were degassed with nitrogen before each measurement. Resonance Raman (rR) measurements were performed through excitation at 643 nm, i.e., a reduction-induced absorption band, by a diode-pumped solid-state DL laser (CrystaLaser, USA) and detected by an IsoPlane 160 spectrometer (Princeton Instruments, USA) with an entrance slit width of 0.05 mm and grating 1200 grooves/mm. The excitation power was set to around 5 mW. The Raman signals were recorded by a thermoelectrically cooled PIXIS eXcelon camera (Princeton Instruments, USA). The band of acetonitrile at 1373 cm^{-1} was used as a reference to normalize intensities and wavenumbers. The recorded resonance Raman spectra were background corrected, and the solvent spectrum was subtracted. Negative contributions in the difference spectra are due to the subtraction of the solvent spectrum.

2 Synthetic Section

2.1 Ferrocenecarboxylic acid chloride (Fc1)

0.60 g (2.60 mmol) of ferrocene carboxylic acid was dispersed in 5 mL dry dichloromethane. 2.00 mL oxalyl chloride was added dropwise (CAUTION strong gas formation). After stirring for 5 h at room temperature (20 $^{\circ}C$), the solvent was removed at 50 $^{\circ}C$ in an argon stream, resulting in a deep red slurry. The product was dissolved in 5.00 mL dry dichloromethane and was used as solution without further purification.

2.2 *O,O*-Diethyl (4-Ferroceneamidobenzyl)phosphonate (PFc1)

To 5.00 mL of the previous generated 0.52 M ferrocene carboxylic acid chloride / dichloromethane solution, 0.70 g (2.90 mmol) *O,O*-Diethyl (4-aminobenzyl)phosphonate and 0.80 mL dry triethylamine was added (a tip of a spatula 4-DMAP can be added to help with the amide formation). The mixture was stirred for 24 h at room temperature (20 °C), quenched with water and extracted three times with 5-10 mL ethyl acetate. The collected organic layers were dried over anhydrous sodium sulfate, and the solvent was removed in vacuum. The product was dissolved in a minimum amount of ethyl acetate and applied for a short Silica60 ash column, removing the Ferrocene carboxylic acid. The remaining amine was removed by a short flash column (same size) on Cu-Silica60 (CuSi). Alternatively, the reaction can be quenched with water and extracted with dichloromethane 3 times. The dichloromethane phases were then washed with 1 M HCl(aq) (3x) and half sat. aqueous sodium carbonate solution (3x). The dichloromethane phases were collected and dried over anhydrous sodium sulfate. Solvent was removed in vacuum, and obtained sticky solid was redissolved in ethyl acetate and applied for a short column with ethyl acetate as eluent. Afterwards the solvent was removed in vacuum, and the crystalline product was dried.

Yield: 1.10 g / 2.41 mmol (93% based on Ferrocene monocarboxylic acid) of an orange-red crystalline solid was obtained.

^{1}H-NMR ([D6]Acetone, 400 MHz) δ[ppm] = 8.84 (s, 1H, NH), 7.81-7.56 (m, 2H, CH(2 / 2′)), 7.41-7.14 (m, 2H, CH(1 / 1′)), 4.96 (dd, J = 1.9 Hz, 1.9 Hz, 2H, CH(α^1/α^2)), 4.42 (dd, J = 1.9 Hz, 1.9 Hz, 2H, CH(β^1/β^2)), 4.23 (s, 5H, CH(Cp′)), 4.00 (dtd, J = 8.1, 7.1, 6.4 Hz, 4H, O-CH$_2$), 3.12 (d, J = 21.3 Hz, 2H, P-CH$_2$), 1.22 (t, J = 7.1 Hz, 6H, CH$_3$).

^{13}C-NMR ([D6]Acetone, 101 MHz) δ[ppm] = 167.95, 138.24 (d, J_{CP} = 3.7 Hz), 129.99 (d, J_{CP} = 6.6 Hz), 127.05 (d, J_{CP} = 9.0 Hz), 119.73 (d, J_{CP} = 2.9 Hz), 76.99, 70.45, 69.54, 68.48, 61.32 (d, J_{CP} = 6.5 Hz), 32.41 (d, J_{CP} = 137.7 Hz), 15.85 (d, J_{CP} = 5.8 Hz).

^{31}P-NMR ([D6]Acetone, 162 MHz) δ[ppm] = 25.91.

IR (ATR-FT, $\tilde{\nu}$ [cm^{-1}]): 3300, 3255, 3185, 3112, 3047, 2977, 2958, 2931, 2907, 2853, 1657, 1632, 1599, 1529, 1513, 1478, 1455, 1441, 1410, 1377, 1346, 1320, 1270, 1223, 1192, 1186, 1157, 1140, 1132, 1103, 1060, 1052, 1031, 1015, 961, 930, 877, 858, 844, 822, 811, 795, 770, 753, 741, 714, 704, 640, 574, 541, 513, 484, 476, 455, 422

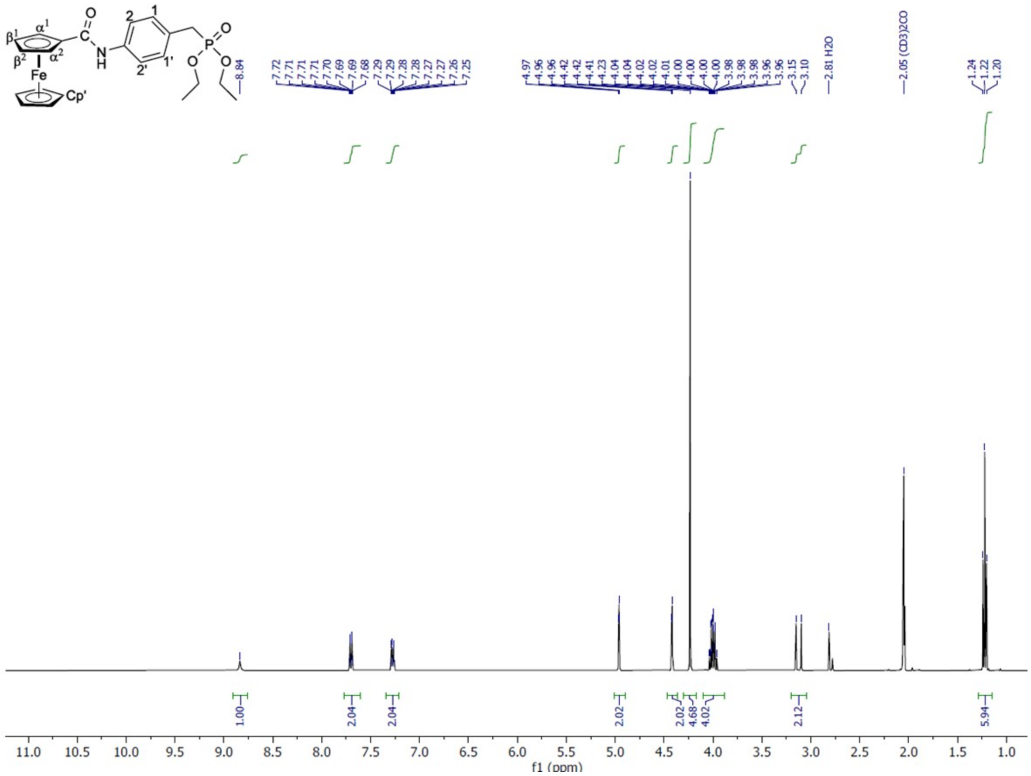

Figure S1: 1H-NMR of *O,O*-Diethyl (4-Ferroceneamidobenzyl)phosphonate (PFc1) in[D6]Acetone.

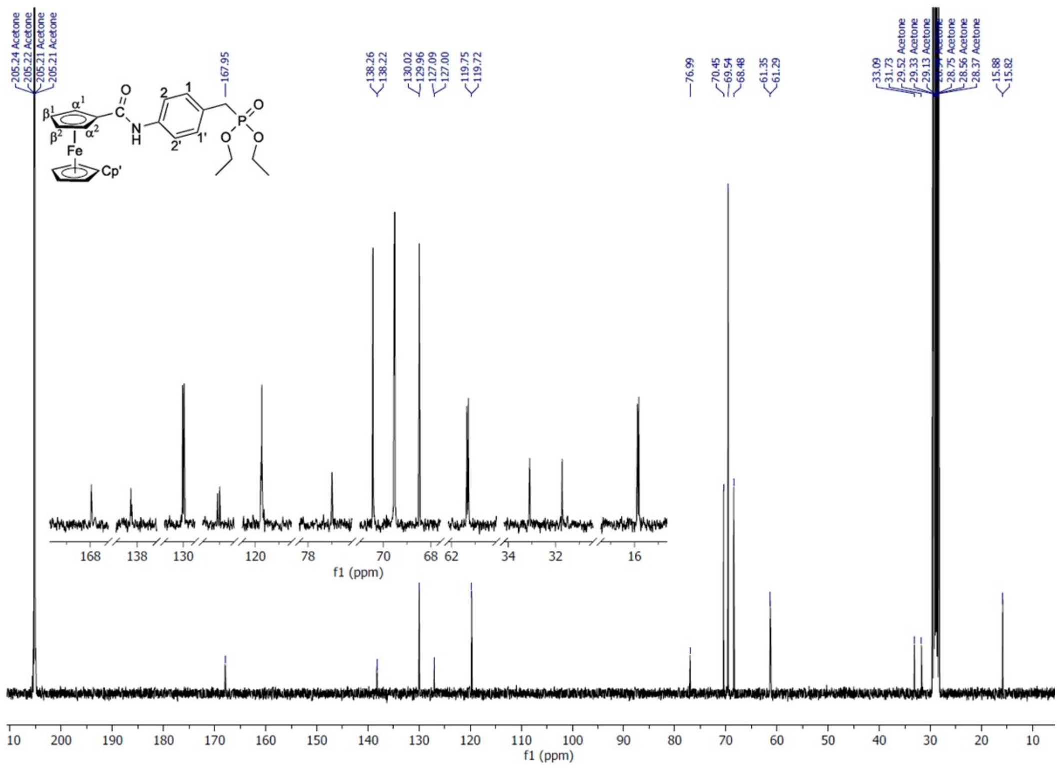

Figure S2: ^{13}C-NMR of *O,O*-Diethyl (4-Ferroceneamidobenzyl)phosphonate (PFc1) in [D6]Acetone.

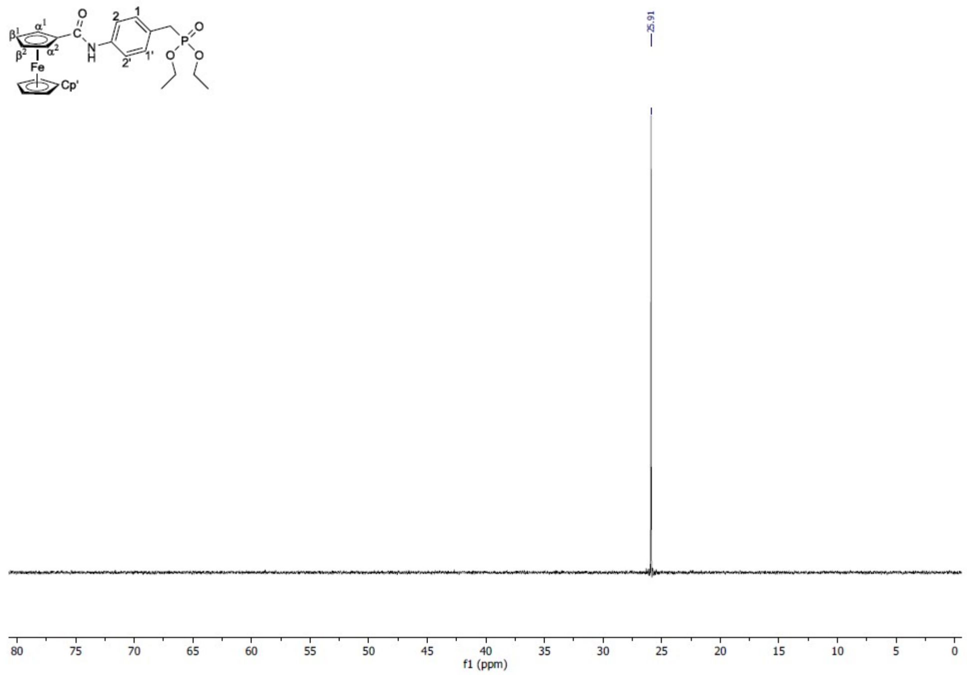

Figure S3: ^{31}P-NMR of *O,O*-Diethyl (4-Ferroceneamidobenzyl)phosphonate (PFc1) in[D6]Acetone.

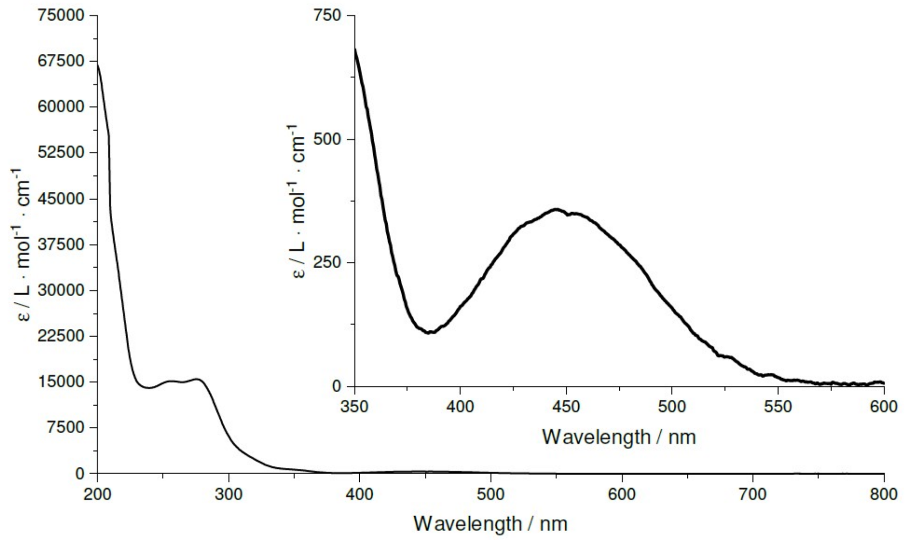

Figure S4: UV-Vis absorption spectrum of *O,O*-diethyl (4-ferrocene-amidobenzyl)phosphonate (PFc1) in acetonitrile.

2.3 *O,O*-Diethyl (4(1'-Acetyl-Ferroceneamidobenzyl))phosphonate (PAcFc1)

To 10 mL dry dichloromethane 0.22 g (1.65 mmol) $AlCl_3$ (anhydrous) was added and cooled to -5 to -10 $^{\circ}C$ (ice / water / sodium chloride) while degassing with argon. After cooling for 10 min, 0.25 g (0.53 mmol) of PFc1 was added and stirred for 5 min. 0.12 mL acetyl chloride, dissolved in 10 mL dry dichloromethane, was added within 20 to 30 min. The mixture was then stirred at 0 $^{\circ}C$ for 60 min, quenched with ice and extracted with ethyl acetate. The combined organic phases were dried over anhydrous sodium sulfate and puri ed via column chromatography with ethyl acetate as eluent.

Yield: 0.14 g (0.28 mmol) 53% based on PFc1.

^{1}H-NMR ([D6]Acetone, 400 MHz) δ[ppm] = 8.85 (s, 1H, NH), 7.72 (d, J = 7.8 Hz, 2H, CH(2 / 2′)), 7.29 (dd, J = 8.6, 2.5 Hz, 2H, CH(1 / 1′)), 4.95 (t, J = 2.0 Hz, 2H, CH(α^{1}/α^{2})), 4.81 (t, J = 1.9 Hz, 2H, CH($\alpha^{1'}/\alpha^{2'}$)), 4.59 (t, J = 2.0 Hz, 2H, CH(β^{1}/β^{2})), 4.49 (t, J = 1.9 Hz, 2H, CH($\beta^{1'}/\beta^{2'}$)), 4.13-3.85 (m, 4H, O-CH_2), 3.13 (d, J = 21.3 Hz, 2H, P-CH_2), 2.36 (s, 3H, CH_3(acetyl)), 1.22 (t, J = 7.0 Hz, 6H, CH_3).

^{13}C-NMR ([D6]Acetone, 101 MHz) δ[ppm] = 200.30, 166.75, 137.96 (d, J_{CP} = 3.6 Hz), 130.00 (d, J_{CP} = 6.6 Hz), 127.37 (d, J_{CP} = 9.2 Hz), 119.93 (d, J_{CP} = 2.9 Hz), 80.85, 78.79, 73.31, 71.89, 70.93, 70.09, 61.33 (d, J_{CP} = 6.6 Hz), 32.44 (d, J_{CP} = 137.7 Hz), 29.69, 26.87, 15.85 (d, J_{CP} = 5.8 Hz).

^{31}P-NMR ([D6]Acetone, 162 MHz) δ[ppm] = 25.87.

IR (ATR-FT, $\tilde{\nu}$ [cm^{-1}]): 3292, 3250, 3182, 3112, 3096, 3042, 2985, 2931, 2907, 2859, 1733, 1665, 1655, 1599, 1527, 1513, 1478, 1453, 1410, 1392, 1369, 1355, 1315, 1274, 1231, 1219, 1198, 1138, 1101, 1064, 1050, 1023, 954, 930, 893, 865, 852, 835, 823, 805, 790, 764, 720, 704, 673, 640, 615, 595, 578, 541, 531, 511, 488, 447, 420.

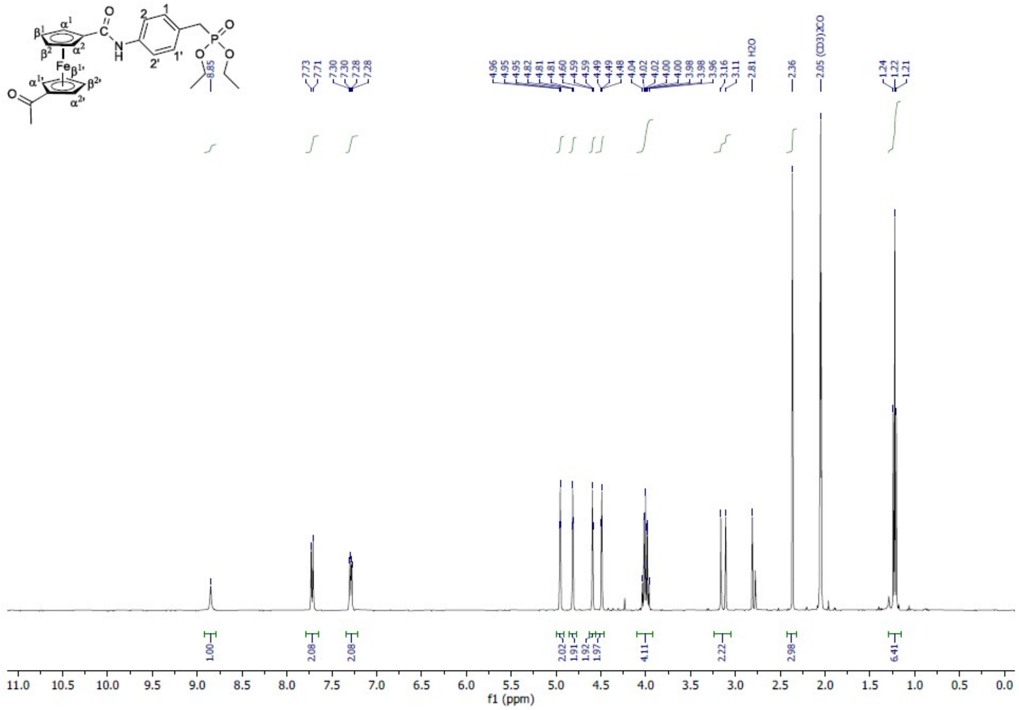

Figure S5: ^{1}H-NMR spectrum of *O,O*-diethyl (4-(1'-acetylferrocene)-amidobenzyl)phosphonate (PAcFc1) in [D6]Acetone.

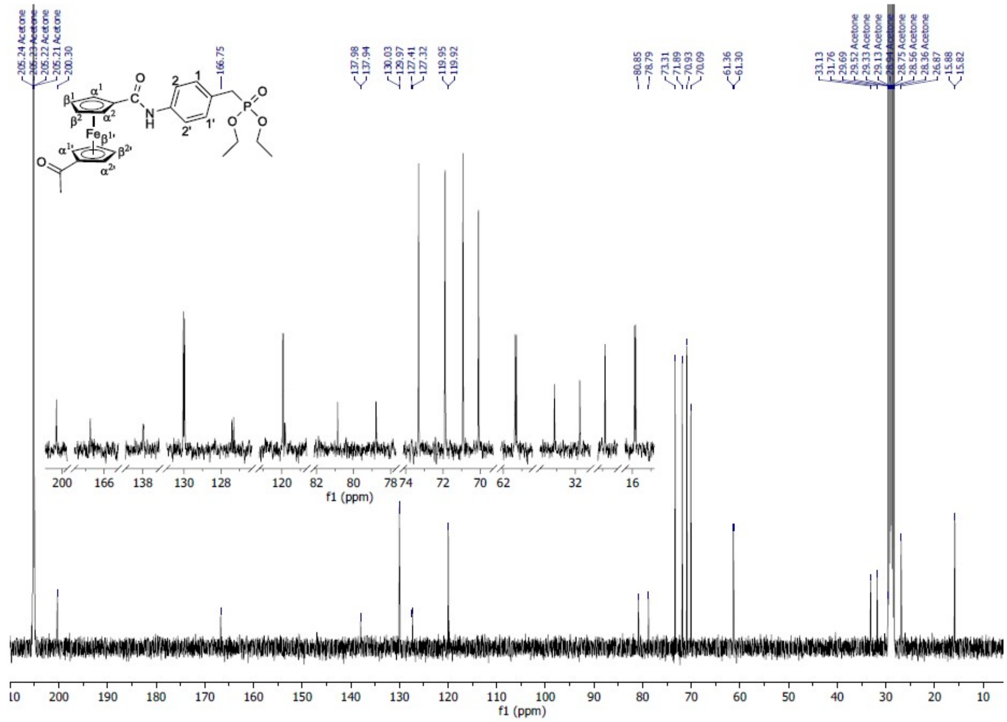

Figure S6: ^{13}C-NMR spectrum of *O,O*-diethyl (4-(1'-acetylferrocene)-amidobenzyl)phosphonate (PAcFc1) in [D6]Acetone.

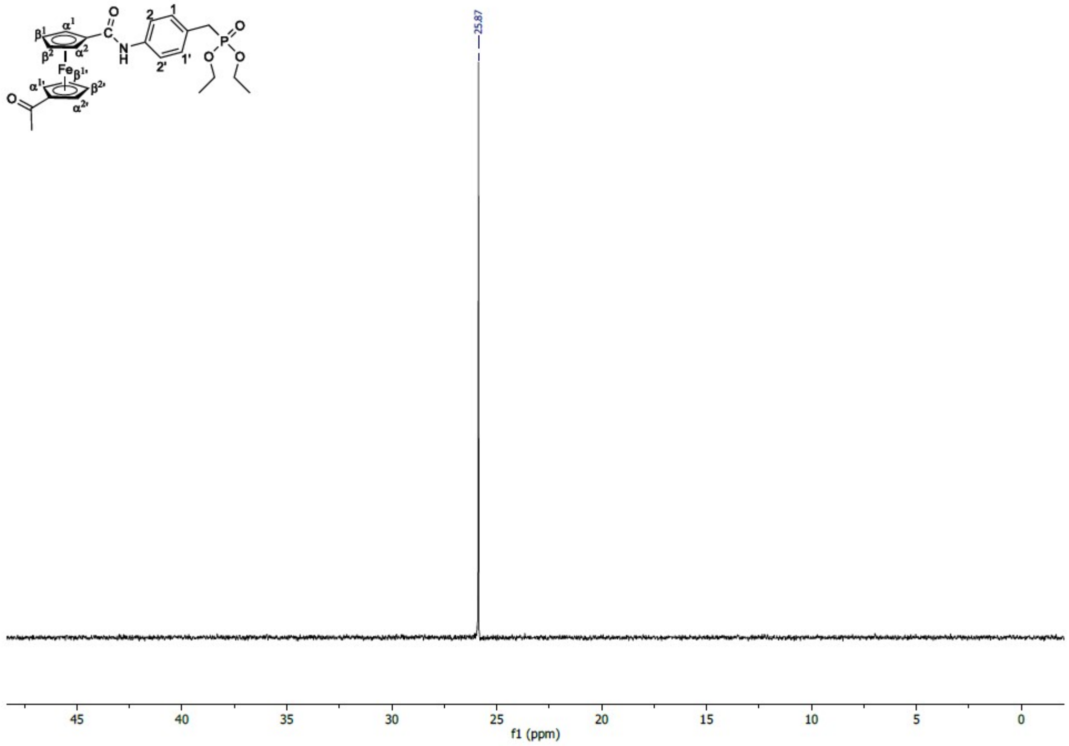

Figure S7: ^{31}P-NMR spectrum of *O,O*-diethyl (4-(1'-acetylferrocene)-amidobenzyl)phosphonate(PAcFc1) in [D6]Acetone.

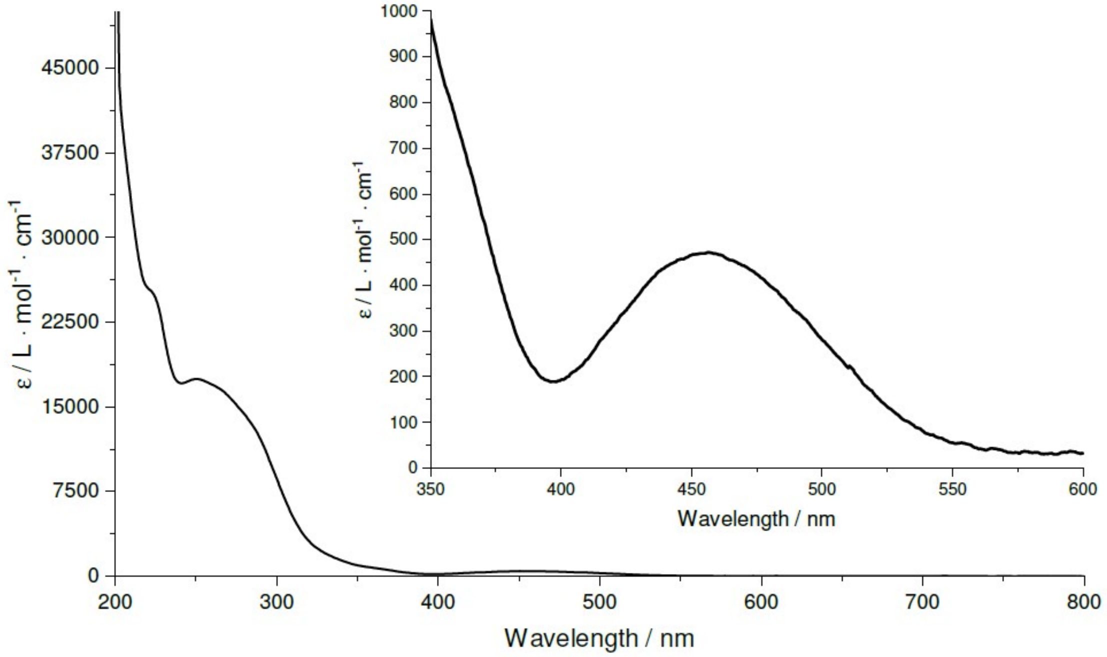

Figure S8: UV-Vis absorption spectrum of *O,O*-diethyl (4-(1'-acetylferrocene)- amidobenzyl) phosphonate (PAcFc1) in acetonitrile.

2.4 Bis(trimethylsilyl) (4-Ferroceneamidobenzyl) phosphonate (PFc2)

0.33 g (0.72 mmol) PFc1 were dissolved in 6.2 mL dry dichloromethane. 0.39 mL trimethyl-bromosilane was added and the mixture was stirred for 24 h at room temperature (20 °*C*). The solvent was removed in an argon stream and dried in vacuum. The resulting product was redissolved in 7.5 mL dry acetonitrile and used as solution without further purification.

2.5 Bis(trimethylsilyl) (4(1'-Acetyl-Ferroceneamidobenzyl)) phosphonate (PAcFc2)

0.20 g (0.40 mmol) PAcFc1 were dissolved in 3.8 mL dry dichloromethane. 0.22 mL trimethyl bromosilane was added and the mixture was stirred for 24 h at room temperature (20 °*C*). The solvent was removed in an argon stream and dried in vacuum. The resulting product was redissolved in 6 mL dry acetonitrile and used as solution without further purification.

2.6 $K_{10}[\alpha_2\text{-}P_2W_{17}O_{61}]$ (C1)

$K_{10}[\alpha_2\text{-}P_2W_{17}O_{61}]$ was synthesized using a literature known procedure.[5]

2.7 $nBu_4N_9K_1[\alpha_2\text{-}P_2W_{17}O_{61}]$ (C2)

$nBu4N_9K_1[\alpha_2\text{-}P_2W_{17}O_{61}]$ was synthesized from C1 using a literature known procedure.[6]

2.8 $nBu_4N_6[\alpha_2\text{-}P_2W_{17}O_{61}(POCH_2C_6H_4NHCOFc)_2]$ (FcPOM-1)

To 5.00 mL PFc2 solution in acetonitrile 1.00 g (0.16 mmol) of cluster C2 was added. The resulting solution was stirred for 48 h at 60 °*C*, resulting in an orange precipitate, which was collected and washed with 20.0 mL diethyl ether, 20.0 mL ethanol and again 20.0 mL diethyl ether. The product was dissolved in 3 mL *N,N*-dimethyl acetamide, 3.00 g of tetra-*n*-butylammonium chloride was added and the product was precipitated by the slow addition of 50.0 mL H_2O. The resulting precipitate was washed with 20.0 mL H_2O, 20.0 mL ethanol and 20.0 mL diethyl ether. After drying in air for one day a yellow powder was obtained. Yield: 0.88 g (0.14 mmol) 88% based on C2.

^{1}H-NMR ([D6]DMSO, 400 MHz) δ[ppm] = 9.38 (s, 2H, NH), 7.58 (d, J = 8.3 Hz, 4H, CH(2 / 2′)), 7.32 (dd, J = 8.7, 2.3 Hz, 4H, CH(1 / 1′)), 5.00 (dd, J = 1.9 Hz, 1.9 Hz, 4H, CH(α^1/α^2)), 4.41 (dd, 4H, J = 1.9 Hz, 1.9 Hz, CH(β^1/β^2)), 4.20 (s, 10H, CH(Cp′)), 3.24-3.08 (m, 48H, CH_2), 3.05 (dd, J = 21.6, 15.1 Hz, 4H, P-CH_2), 1.69-1.44 (m, 48H, CH_2), 1.44-1.21 (m, 48H, CH_2), 0.94 (t, J = 7.2 Hz, 72H, CH_3).

^{31}P-NMR ([D6]DMSO, 162 MHz) δ[ppm] = 23.36 ($RPO(OR)_2$), -11.35 (PO_4), -12.97 (PO_4).

IR (ATR-FT, $\tilde{\nu}$ [cm^{-1}]): 2962, 2935, 2874, 1658, 1594, 1516, 1483, 1463, 1412, 1379, 1318, 1267, 1246, 1156, 1089, 1052, 997, 954, 907, 812, 787, 737, 597, 567, 528, 481, 422.

CV (100 mV · s^{-1}, 0.1 M nBu$_4$NPF$_6$, c = 1 mM, E [V]): -0.74 ($W^{V\ I/V}$), -1.17 ($W^{V\ I/V}$), -1.81 ($W^{V\ I/V}$), -2.22 ($W^{V\ I/V}$), 0.13 (Fc/Fc^+).

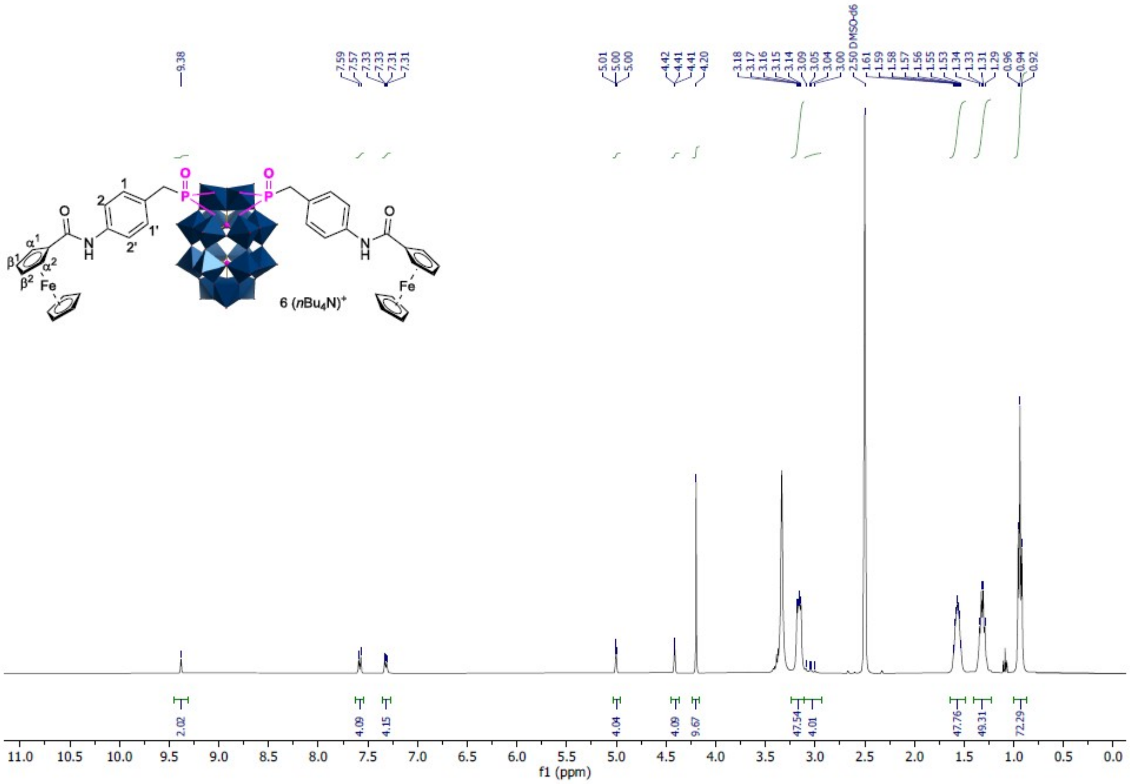

Figure S9: ^{1}H-NMR spectrum of α_2-Dawson-Ferrocene tetra-*n*-butylammonium salt (**FcPOM-1**) in [D6]DMSO.

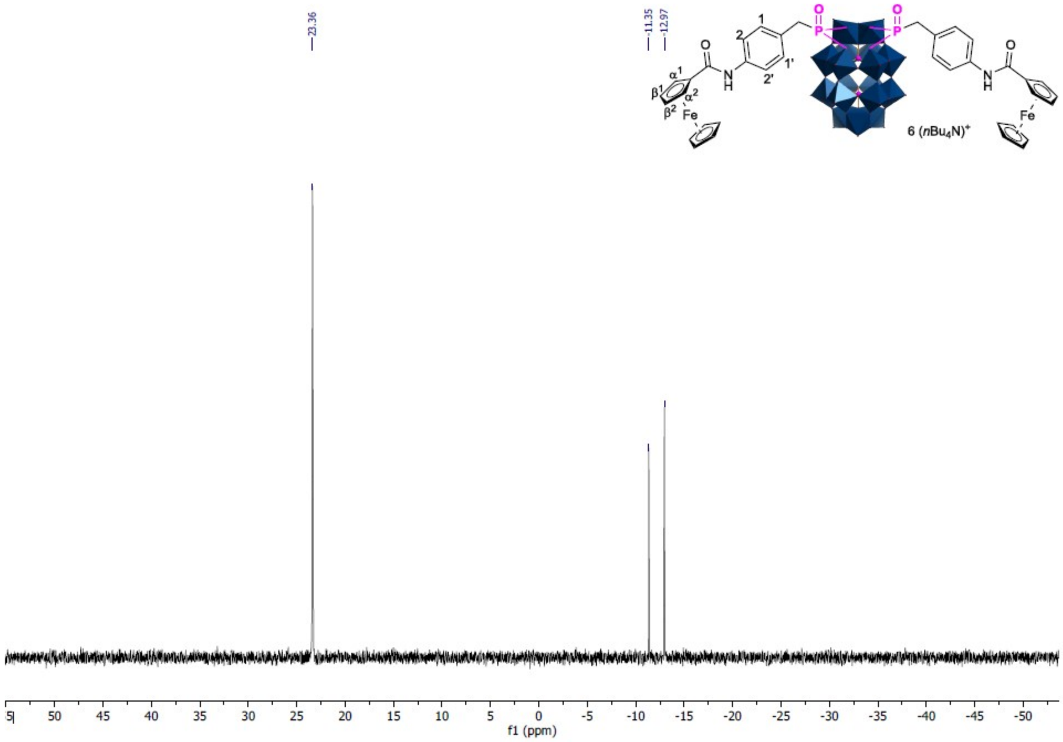

Figure S10: ^{31}P-NMR spectrum of α_2-Dawson-Ferrocene tetra-*n*-butylammonium salt (**FcPOM-1**) in[D6]DMSO.

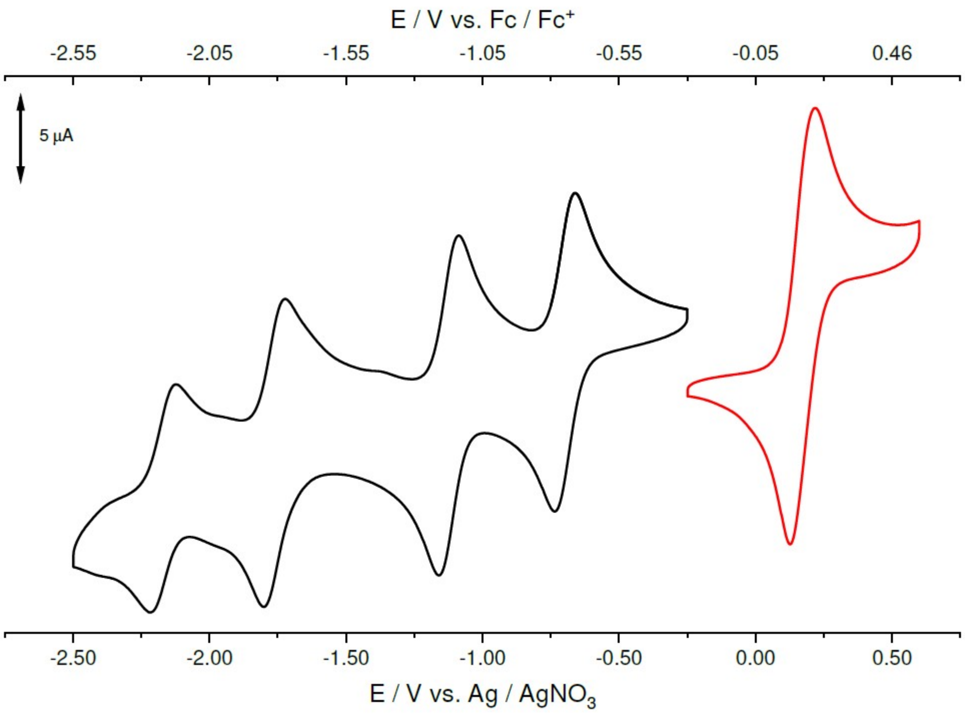

Figure S11: Cyclic voltammetry of 1 mM α_2-Dawson-Ferrocene tetra-*n*-butylammonium salt (**FcPOM-1**) in *N,N*-dimethylformamide with 0.1 M $n\text{Bu}_4\text{NPF}_6$ as supporting electrolyte.

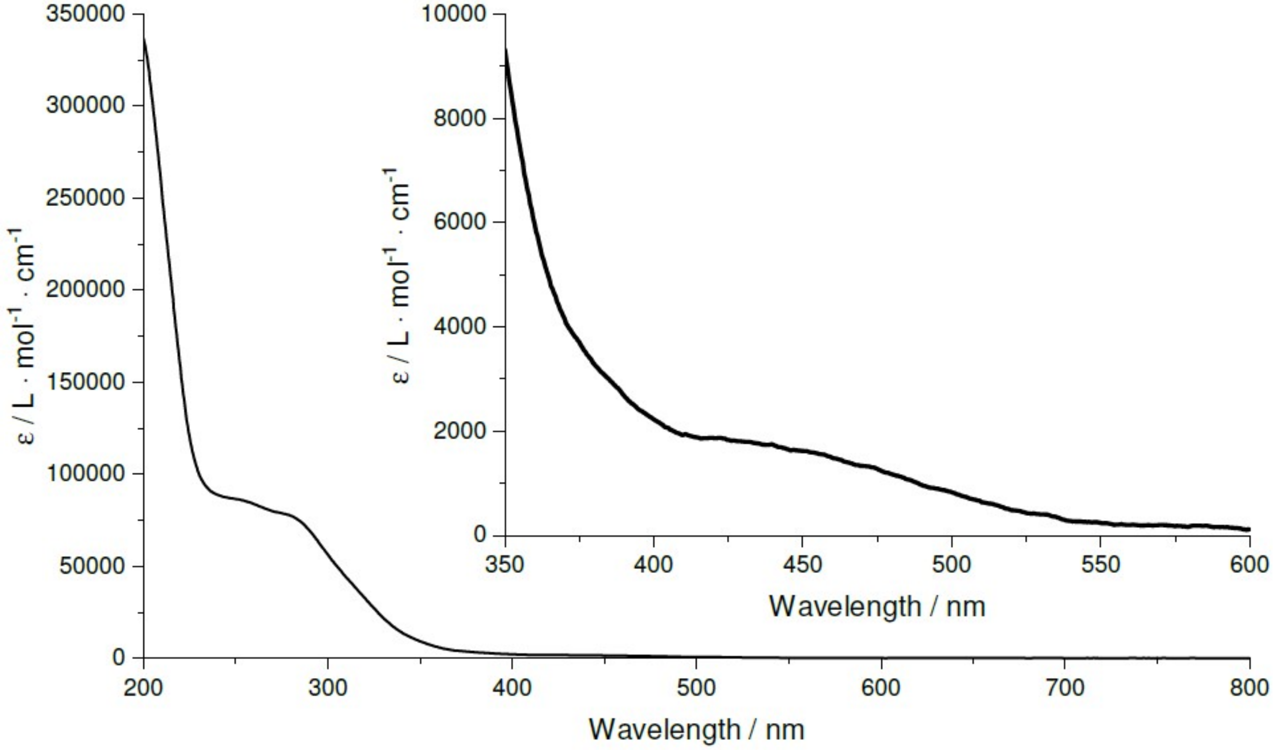

Figure S12: UV-Vis absorption spectrum of α_2-Dawson-Ferrocene tetra-*n*-butylammonium salt (**FcPOM-1**) in acetonitrile.

2.9 $n\text{Bu}_4\text{N}_6[\alpha_2\text{-P}_2\text{W}_{17}\text{O}_{61}(\text{POCH}_2\text{C}_6\text{H}_4\text{NHCOAcFc})_2]$ (FcPOM-2)

To 6 mL PAcFc2 solution in acetonitrile 0.86 g (0.13 mmol) of cluster C2 was added. The resulting solution was stirred for 48 h at 60 °*C*, resulting in an orange precipitate, which was collected and washed with 20 mL diethyl ether, 20 mL ethanol and again 20 mL diethyl ether. The product was dissolved in 3 mL *N,N* -dimethyl acetamide, 3 g of tetra-n-butylammonium chloride was added and the product was precipitate by the slow addition of 50 mL H_2O. The resulting precipitate was washed with 20 mL H_2O, 20 mL ethanol and 20 mL diethyl ether. After drying in air for one day a yellow-orange powder was obtained.

Yield: 0.74 g (0.12 mmol) 92% based on C2.

^{1}H-NMR ([D6]DMSO, 400 MHz) δ[ppm] = 9.47 (s, 2H, NH), 7.58 (d, J = 8.2 Hz, 4H, CH(2 / 2')), 7.33 (dd, J = 8.8, 2.7 Hz, 4H, CH(1 / 1')), 5.03(3) (d, J = 2.1 Hz, 2H, CH(α^2)), 5.02(8) (d, J = 2.1 Hz, 2H, CH(α^1/α^2)), 4.75 (dd, J = 2.1 Hz, 2.1 Hz, 4H, CH($\alpha^{1'}/\alpha^{2'}$)), 4.56 (dd, J = 2.0 Hz, 2.0 Hz, 4H, CH(β^1/β^2)), 4.46 (dd, J = 2.0 Hz, 2.0 Hz, 4H, CH($\beta^{1'}/\beta^{2'}$)), 3.16 (tt, J = 22.8, 8.4 Hz, 48H, CH_2), 3.05 (dd, J = 22.1, 14.9 Hz, 4H, P-CH_2), 2.31 (s, 6H, CH_3(acetyl)), 1.57 (p, J = 7.4 Hz, 48H, CH_2), 1.32 (q, J = 7.3 Hz, 48H, CH_2), 0.94 (t, J = 7.2 Hz, 72H, CH_3).

^{31}P-NMR ([D6]DMSO, 162 MHz) δ[ppm] = 23.34 ($RPO(OR)_2$), -11.36 (PO_4), -12.97 (PO_4).

IR (ATR-FT, $\tilde{\nu}$ [cm^{-1}]): 2960, 2933, 2872, 1659, 1595, 1517, 1482, 1455, 1412, 1377, 1352, 1320, 1305, 1278, 1245, 1198, 1159, 1126, 1089, 1056, 1040, 996, 982, 968, 953, 943, 920, 908, 811, 786, 737, 597, 585, 568, 530, 482, 471, 453, 441, 424, 410.

CV (100 mV · s^{-1}, 0.1 M $n$$Bu_4NPF_6$, c = 1 mM, E [V]): -0.75 ($W^{V\ I/V}$), -1.18 ($W^{V\ I/V}$), -1.82 ($W^{V\ I/V}$), -2.24 ($W^{V\ I/V}$), 0.408 (Fc/Fc^+).

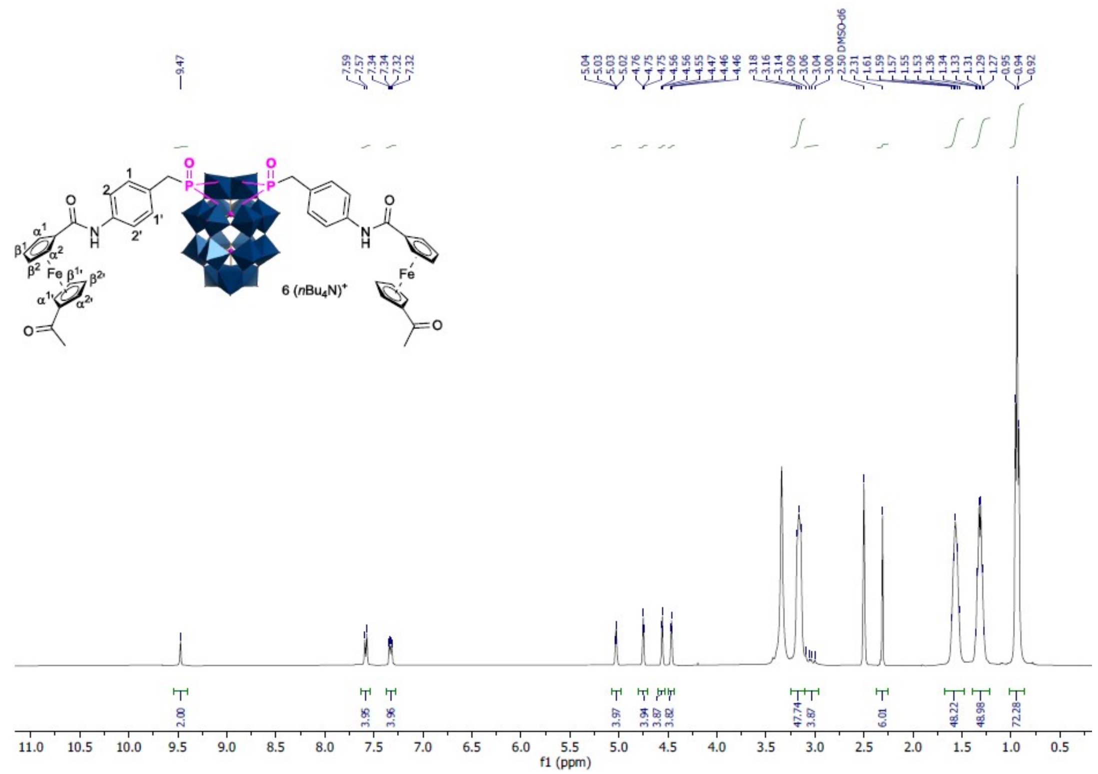

Figure S13: 1H-NMR of α_2-Dawson-AcetylFerrocene tetra-*n*-butylammonium salt (**FcPOM-2**) in [D6]DMSO.

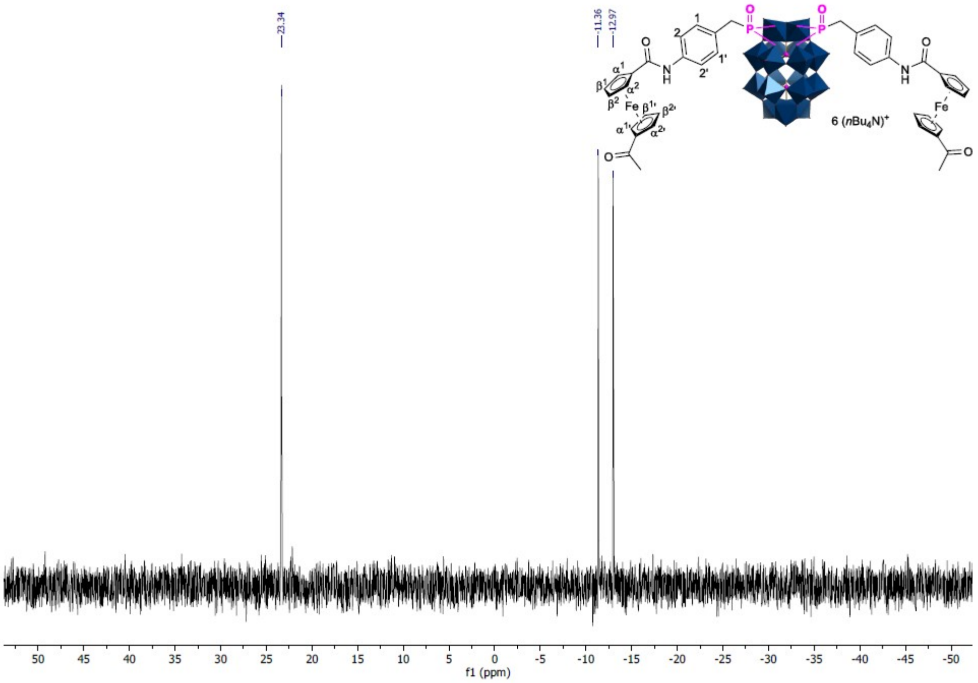

Figure S14: ^{31}P-NMR spectrum of α_2-Dawson-AcetylFerrocene tetra-*n*-butylammonium salt (**FcPOM-2**) in [D6]DMSO.

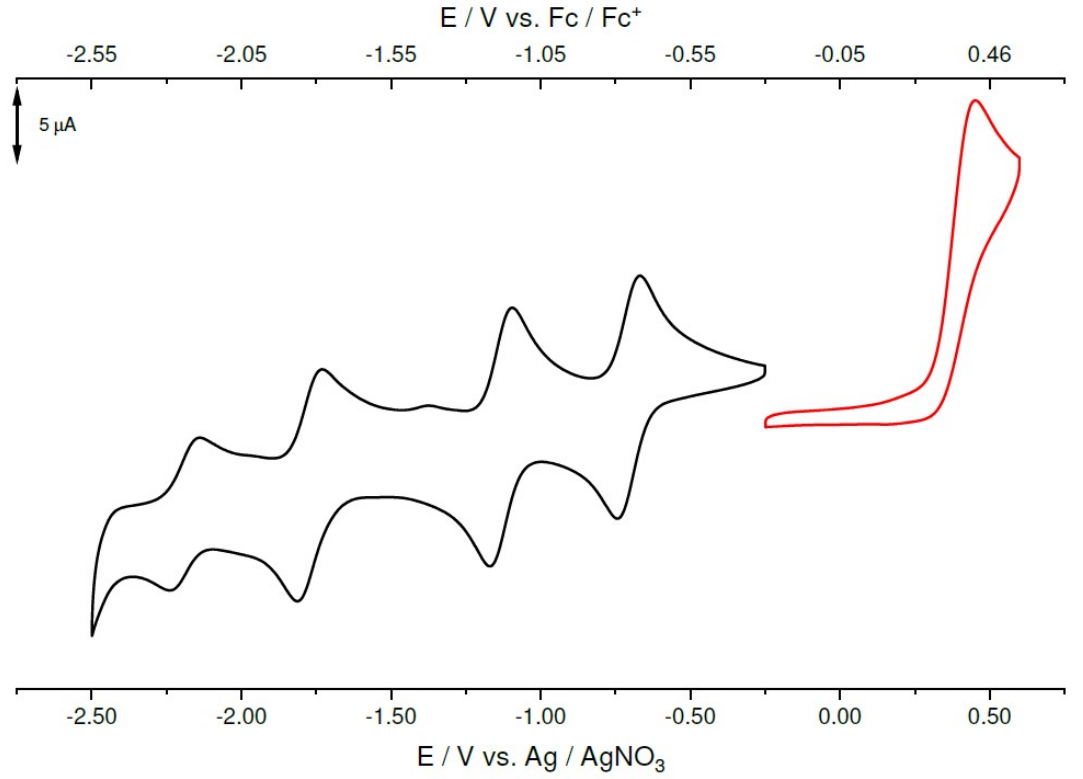

Figure S15: Cyclic voltammetry of 1 mM α_2-Dawson-AcetylFerrocene tetra-*n*-butylammonium salt (**FcPOM-2**) in *N,N* -dimethylformamide with 0.1 M $n\mathrm{Bu_4NPF_6}$ as

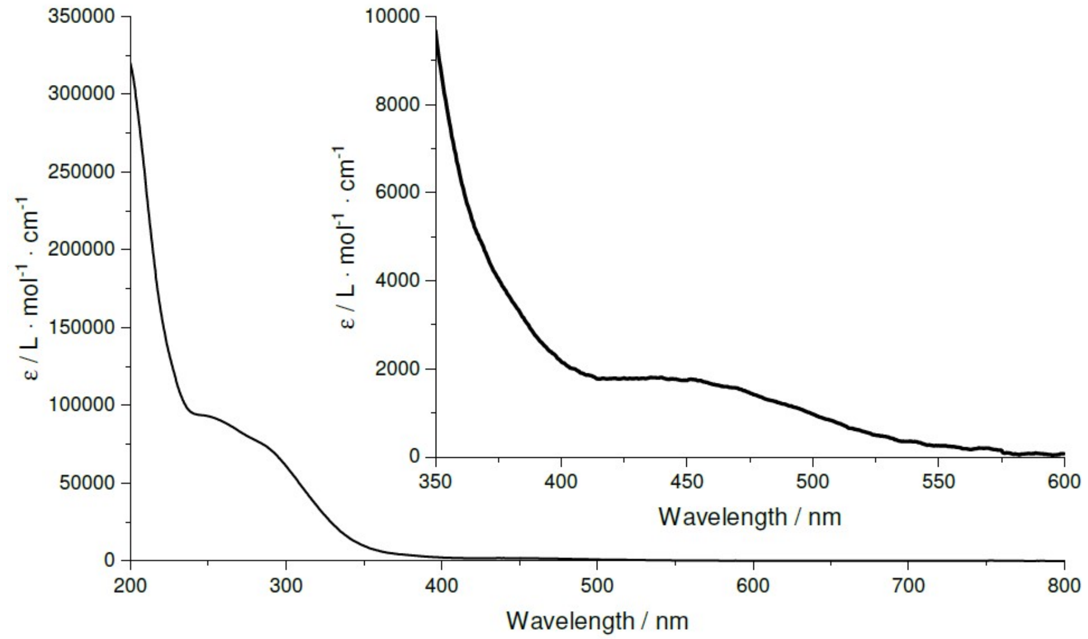

supporting electrolyte.

Figure S16: UV-Vis absorption spectrum of α_2-Dawson-AcetylFerrocene tetra-*n*-

butylammonium salt (**FcPOM-2**) in acetonitrile.

2.10 Copper(II) functionalized Silica for amine removal (CuSi)

50 g of Mesh 60 Silica (SiO_2) were dispersed in acetone and 2 g of $Cu(BF_4)_2 \cdot 2\ H_2O$ was added. The suspension was stirred for 30 min, filtered over a folded paper filter, washed with 100 mL acetone and dried for one day at air. Pale blue colored silica was obtained.

3 Characterization

3.1 ^{1}H-NMR studies

The ^{1}H-NMR spectra reveal distinct chemical shifts for the amide protons. For both free ligands (PFc1 / PAcFc1), the amide proton resonance appears at $\delta = 8.85 \pm 0.01$ ppm. Upon covalent attachment to the α_2-Dawson cluster (C2), this resonance shifts downfield to $\delta = 9.38$ ppm for **FcPOM-1** and $\delta = 9.47$ ppm for **FcPOM-2**. This downfield shift upon cluster binding is attributed to the electric field generated by the clusters' negative charge, which interacts with and further polarizes the N-H bond. The aromatic protons of the ipso-para substituted phenyl system resonate between $\delta = 7.10$ and 7.80 ppm, with minimal changes observed upon cluster binding. The ferrocene proton resonances provide more detailed structural information. While the overall structure of the ligands remains largely unchanged upon cluster attachment, the spin systems of the ferrocene moieties are of particular interest. Comparison of experimental and simulated spectra for clusters **FcPOM-1** and **FcPOM-2** reveals key differences. As expected, **FcPOM-1** exhibits signals for the ten equivalent protons of the Cp′ rings due to rapid rotation about the Cp′-Fe axis. The apparent triplets in the spectrum are overlapping doublets of doublets arising from an A-B-C-D spin system with very similar $^2J_{HH}$ and $^3J_{HH}$ coupling constants. These protons are designated α and β with respect to the carbonyl position. Further analysis of PFc1 demonstrates that the α-protons are chemically and magnetically equivalent (within experimental error), as are the β-protons. Consequently, only couplings between α^1 and β^1/β^2, and between α^2 and β^1/β^2, are observed. In the case of PAcFc1, the Cp′ proton signals are no longer observed. Instead, a second set of α/β protons (designated with a prime) is observed, corresponding to the second Cp ring. While both Cp rings exhibit A-B-C-D spin systems, the α-protons in the Cp ring attached to the amide are no longer chemically equivalent, resulting in two overlapping doublets. The coupling constants are too similar to resolve separate signals, and no coupling between α^1 and α^2 protons is observed. Experimental and simulated spectra are shown in Figure S17.

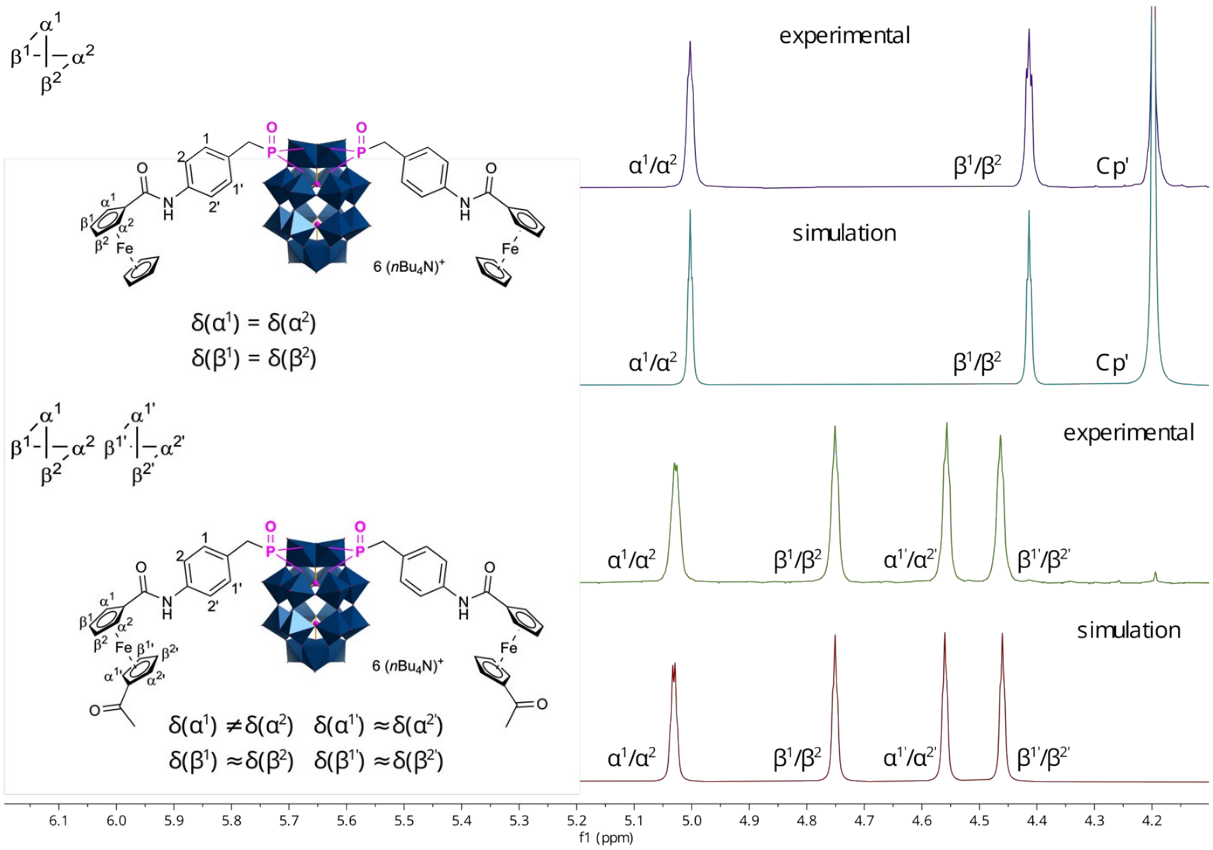

Figure S17: Comparison of experimental and simulated NMR spectra for **FcPOM-1** (top) and **FcPOM-2** (bottom).

3.2 Photoreduction

A solution of **FcPOM-1** salt (1.9 mg, $3.0 \cdot 10^{-4}$ mmol) in 2.8 mL degassed acetonitrile with 0.2 mL (1.5 mmol) triethylamine was irradiated with a 400 nm or 470 nm LED light source. Time-lapse UV-Vis absorption spectra were recorded between t = 0 and 3600 s (Figures S18 and S19). A dark control (Figure S20) revealed some activity due to stray light and the UV-Vis spectrometer's measurement beam. Initial reduced cluster formation, likely due to ambient light exposure during preparation and trace dust contamination, was unavoidable.

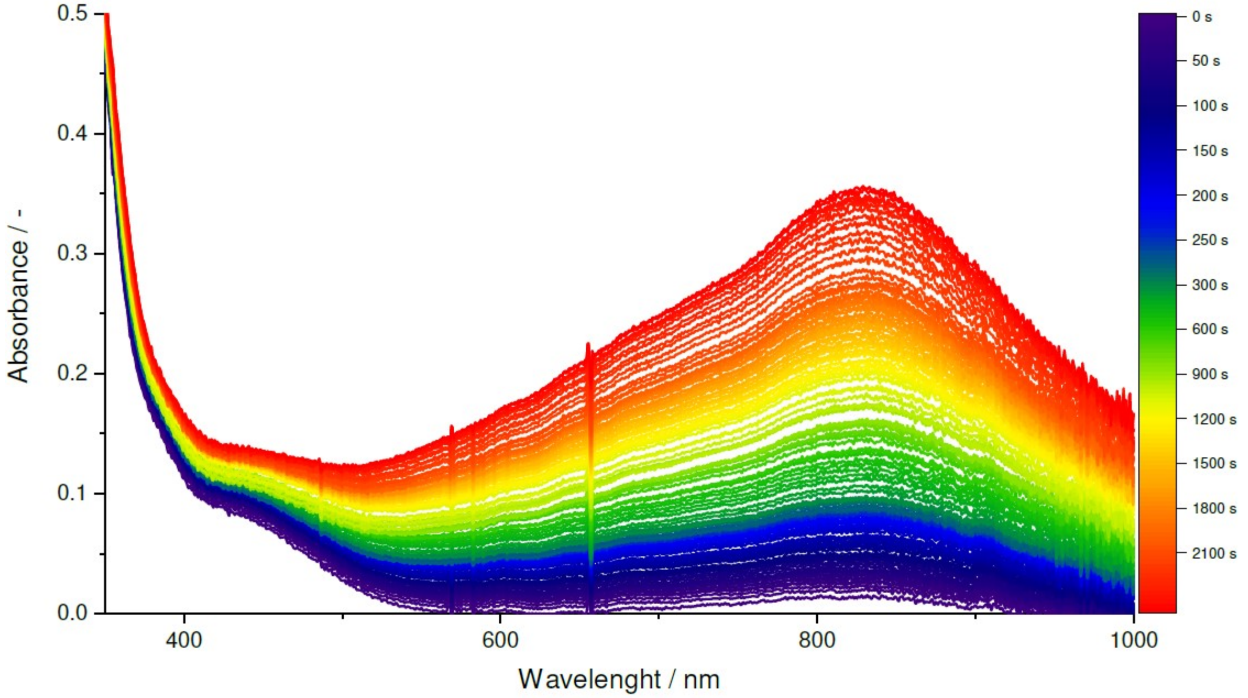

Figure S18: UV-Vis absorption spectra of the **FcPOM-1** photoreduction with a λ_{exc} = 470 nm LED light source in acetonitrile.

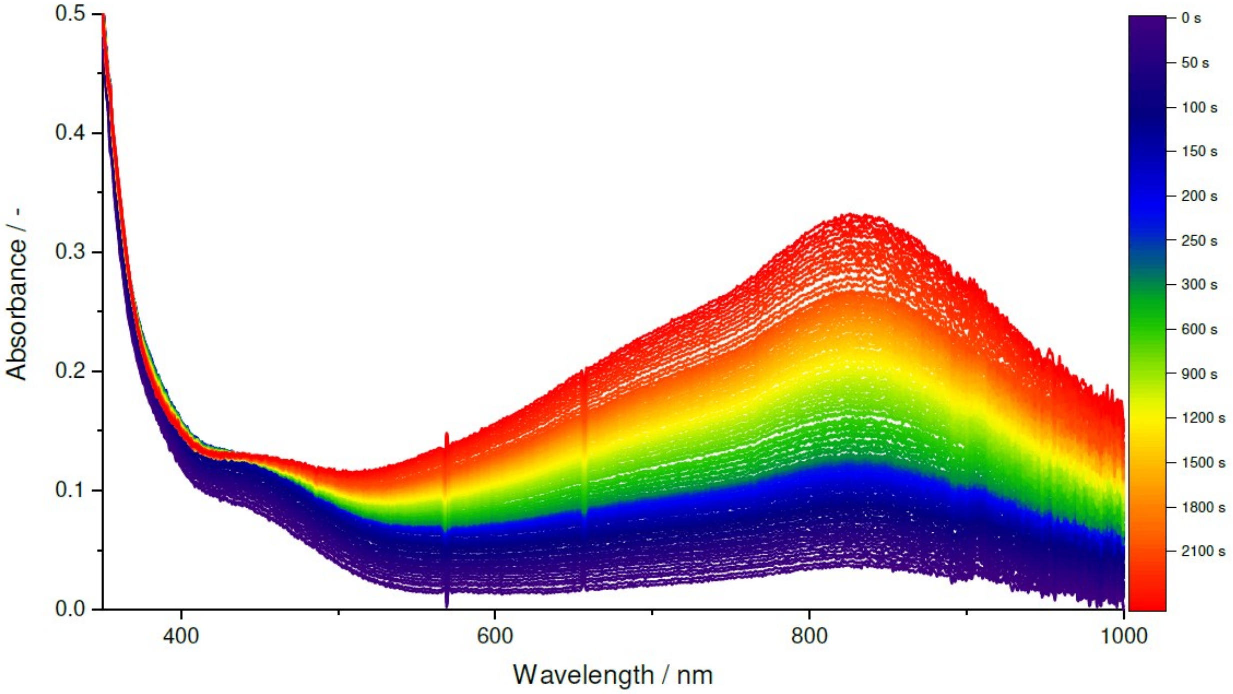

Figure S19: UV-Vis absorption spectra of the **FcPOM-1** photoreduction with a λ_{exc} = 400 nm LED light source in acetonitrile.

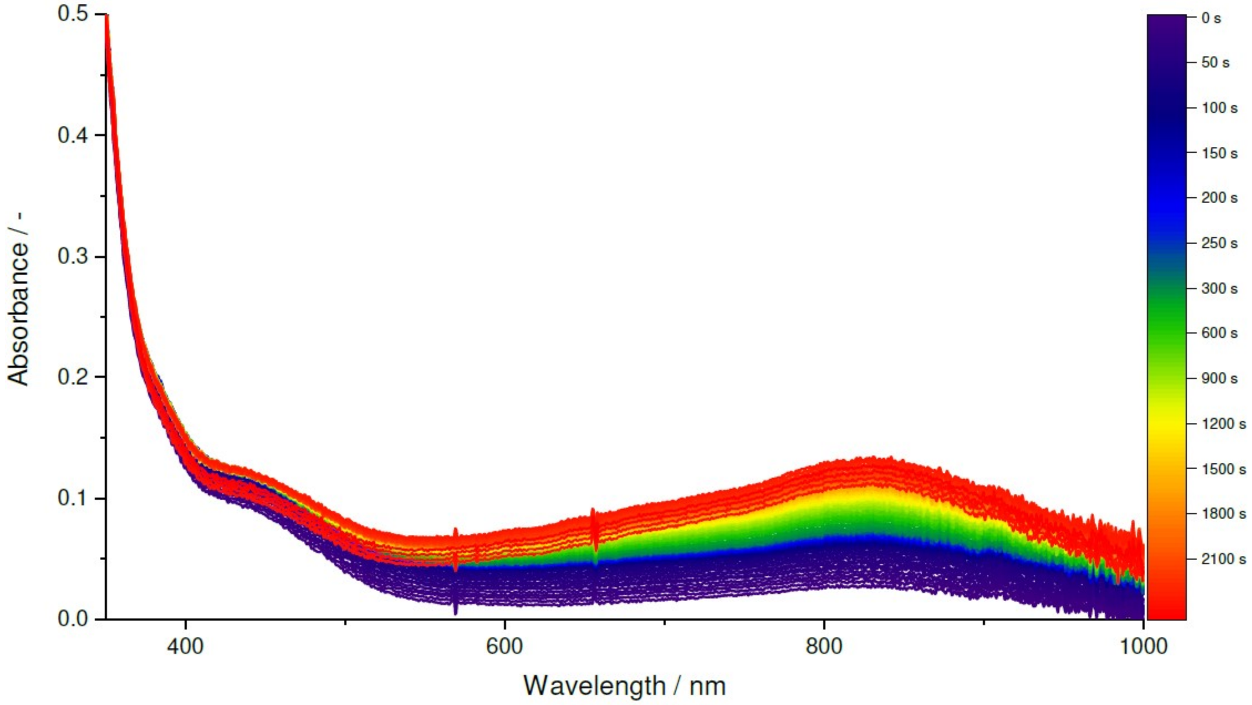

Figure S20: UV-Vis absorption spectra of the **FcPOM-1** photoreduction without irradiation. (Note: the dark reference shows some activity which is assigned to stray light as well as the measurement beam from the UV-Vis spectrometer. Initial reduced cluster could not be avoided due to light pollution during preparation.)

Comparative photoreduction studies

In-situ UV-Vis-NIR spectroscopy of the photoreduction of FcPOM-1 was compared with two non-covalent intermolecular reference systems (PFc1 + $[P_2W_{17}O_{57}(PO_3C_7H_7)_2]^{6-}$; PFc1 + $[P_2W_{17}O_{61}]^{10-}$ in water-free, de-aerated MeCN in the presence of the sacrificial electron donor triethylamine (0.5 M) by LED irradiation, λmax = 467 nm.

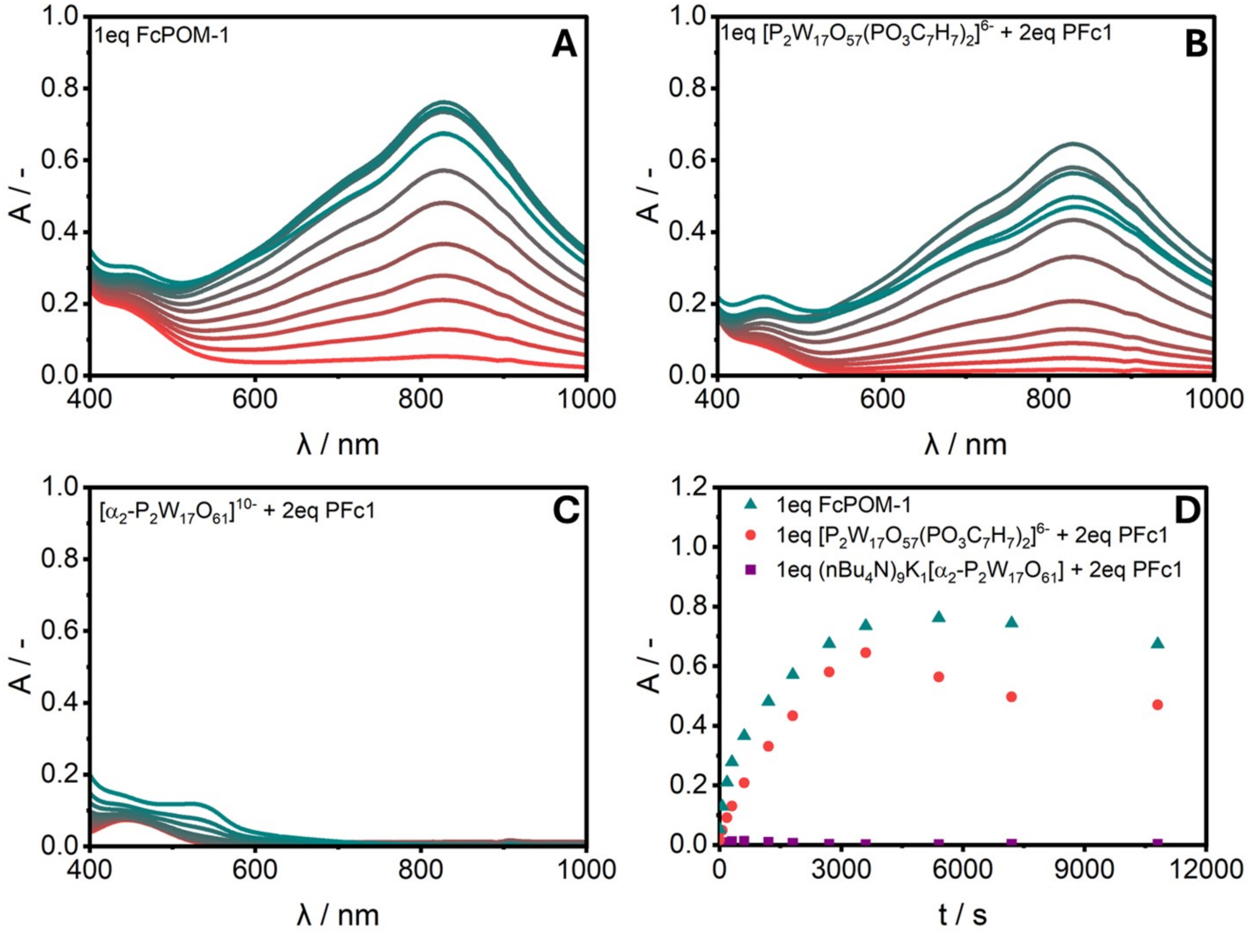

Figure S21: In-situ UV-Vis-NIR spectroscopy of the photoreduction of FcPOM-1(**A**) was compared with two non-covalent intermolecular reference systems (PFc1 + $[P_2W_{17}O_{57}(PO_3C_7H_7)_2]^{6-}$ (**B**); PFc1 + $[P_2W_{17}O_{61}]^{10-}$ (**C**) in water-free, de-aerated MeCN in the presence of the sacrificial electron donor triethylamine (0.5 M) by LED irradiation, λmax = 467 nm.). The Kinetic trace (**D**) of the emergence of the characteristic IVCT band (λmax = 828 nm) indicates one-electron reduction of the Polyoxometalate.

Determination of photonic efficiency

The photonic efficiency was determined based on the formation of the characteristic POM IVCT band (500 nm – 1100 nm) emerging upon irradiation at 467 nm. The concentration of the reduced species was determined using the Beer–Lambert law, where A is the absorbance at 828 nm, l is the optical path length of the cuvette (1 cm) and the molar extinction coefficient is 6500 $M^{-1}cm^{-1}$ (as reported previously based on spectro-electrochemical data).[7]

$$c_{IVCT} = \frac{A}{\varepsilon \cdot l}$$

The amount of reduced POM species formed was calculated as follows, where V is the sample volume:

$$n_{IVCT} = c_{IVCT} \cdot V$$

The number of incident photons was calculated from the measured LED power P, irradiation time t, and irradiation wavelength λ:

$$n_{photon} = \frac{P \cdot t \cdot \lambda}{h \cdot c \cdot N_A}$$ where:

h is Planck's constant,

c is the speed of light,

N_A is Avogadro's constant.

The photonic efficiency was then calculated as:

$$\Phi = \frac{n_{IVCT}}{n_{photon}}$$

Optical power measurements for the LEDs used were performed using a portable PM400 optical power and energy meter console from Thorlabs equipped with a calibrated S121C photodiode power sensor.

The photonic efficiencies where determined after 60 seconds of illumination:

Φ(**FcPOM-1**) = 15.1 x 10^{-3}

$\Phi[P_2W_{17}O_{57}(PO_3C_7H_7)_2]^{6-}$ 5.7 x 10^{-3}

$\Phi[P_2W_{17}O_{61}]^{10-}$= 0.53 x 10^{-3}

3.3 Generation of ferrocenium species monitored by EPR

Photoinduced generation of ferrocenium by in situ irradiation

For irradiation-EPR experiments, stock solutions in acetonitrile were prepared:

FcPOM-1 salt (7.94 mg / 0.25 mL), PFc1 (11.4 mg / 2.5 mL), nitrosobenzene (6.7 mg / 0.25 mL), and imidazole (17.02 mg / 0.25 mL). For each experiment, 50 µL of the cluster or ligand solution, 50 mL of the nitrosobenzene solution, and 20 µL of the imidazole solution were combined. For imidazole-free measurements, 20 µL of acetonitrile replaced the imidazole solution to maintain consistent concentrations. After mixing, 50 µL of the resulting solution was transferred to a 50 µL capillary and loaded into the EPR spectrometer. EPR spectra were recorded every 60 s for 1800 s. For light-dependent measurements, a λ_{ex} = 470 ± 20 nm / 1 W LED was activated after the first 60 s (i.e., beginning with the second spectrum).

EPR spectrometer settings (reaction profile) PFc1 and FcPOM-1

Sweep Range: 330 - 350 mT; Sweep Time: 20 s; Modulation: 0.2 mT; Modulation Frequency: 100 kHz; Microwave Power: 20 mW; Filter Parameter: 0.2; Filtertype: DIG.

EPR spectrometer settings FcPOM-1 (slow measurement):

Sweep Range: 332 - 342 mT; Sweep Time: 90 s; Modulation: 0.025 mT; Modulation Frequency: 100 kHz; Microwave Power: 50 mW; 20 accumulations; Filter Parameter: 0.2; Filtertype: DIG.

Electrochemical generation of ferrocenium by bulk electrolysis (BE):

For bulk electrolysis, 16.0 mg (2.50 mmol) of **FcPOM-1** and 2.27 mg (5.00 mmol) of PFc1 were separately dissolved in 3.00 mL of dry, degassed acetonitrile. Nitrosobenzene (6.80 mg, 63.5 mmol) and imidazole (6.8 mg, 100 mmol) were added to each ferrocene solution. Bulk electrolysis (BE) was performed at a potential of E = 500 mV vs. $Ag/AgNO_3$ for 5400 s, using a carbon paper working electrode and a platinum wire counter electrode. Immediately following the completion of the BE, a 50 µL aliquot was transferred to a 50 µL EPR measurement capillary, sealed, and loaded into the EPR spectrometer. Simulation data are presented in Tables S1 and S2.

EPR spectrometer settings:

Sweep Range: 330 - 345 mT; Sweep Time: 120 s; Modulation: 0.2 mT; Modulation Frequency: 100 kHz; Microwave Power: 50 mW; 5 accumulations; Filter Parameter: 0.2; Filter type: DIG.

Table S1: EPR fitting parameters for **FcPOM-1** with nitrosobenzene and imidazole during bulk electrolysis at E = 500 mV. RMSD = 0.0403.

g /-	A_N / mT	A_H / mT	A_H / mT	A_H / mT	A_H / mT	A_H / mT	A_H / mT	lwpp (gaussian)	lwpp (lorentzian)

2.0060	0.911 (1x)	0.639 (2x)	0.253 (2x)	0.091 (1x)	0.397 (1x)	0.106 (1x)	0.068 (1x)	0.065	0.103
2.0058	1.269 (1x)	0.147 (2x)	0.316 (2x)	0.094 (1x)	-	-	-	0.088	0.106

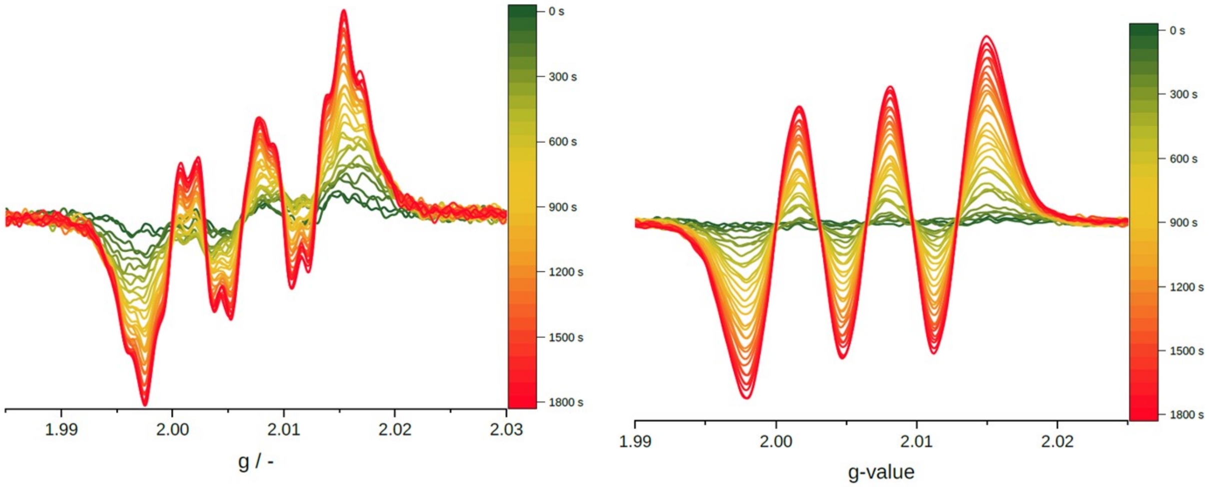

Figure S22: Left: EPR signals of **FcPOM-1**$^+$ trapped with nitroso benzene and imidazole. Photochemical oxidation with λ_{ex} = 470 nm (0 - 1800 s). Right: EPR signals of PFc1$^+$ trapped with nitroso benzene and imidazole. Photochemical oxidation with λ_{ex} = 470 nm (0 - 1800 s).

Table S2: EPR fitting parameters for PFc1 with nitrosobenzene and imidazole during bulk electrolysis at E = 500 mV. RMSD = 0.0526.

g /-	A_N / mT	A_H / mT	A_H / mT	A_H / mT	A_H / mT	A_H / mT	A_H / mT	lwpp (gaussian)	lwpp (lorentzian)
2.0058	1.000 (1x)	0.227 (2x)	0.123 (2x)	0.130 (1x)	0.306 (1x)	0.123 (1x)	0.091 (1x)	0.088	0.160
2.0059	1.298 (1x)	0.132 (2x)	0.252 (2x)	0.106 (1x)	-	-	-	0.121	0.212
Additional radicals were neglected during simulation.									

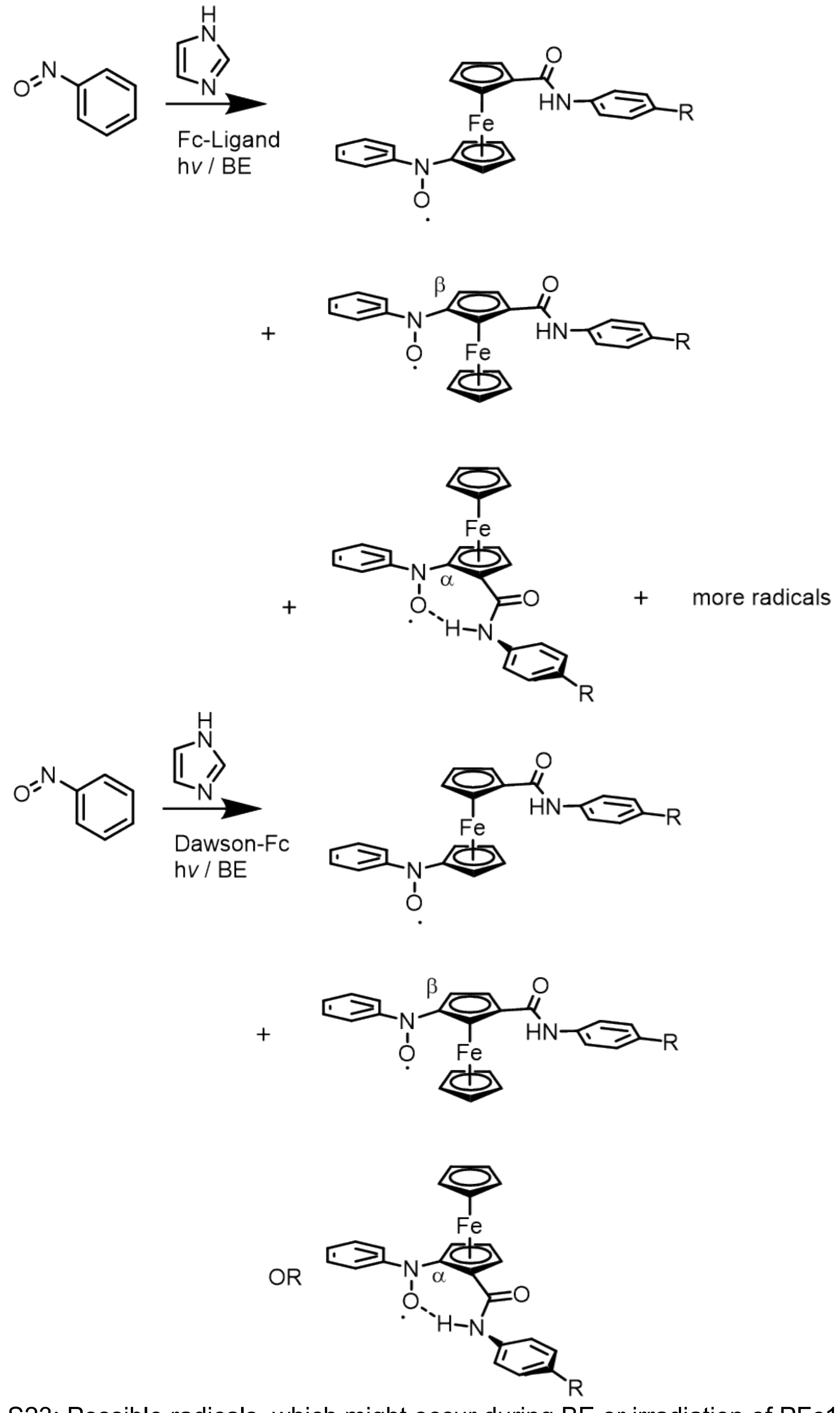

Figure S23: Possible radicals, which might occur during BE or irradiation of PFc1 (top) and **FcPOM-1** (bottom) by the addition of nitroso benzene and imidazole. R denotes either the benzyl phosphonate tether part or benzyl phosphonate anchored at the α_2-Dawson cluster (C2).

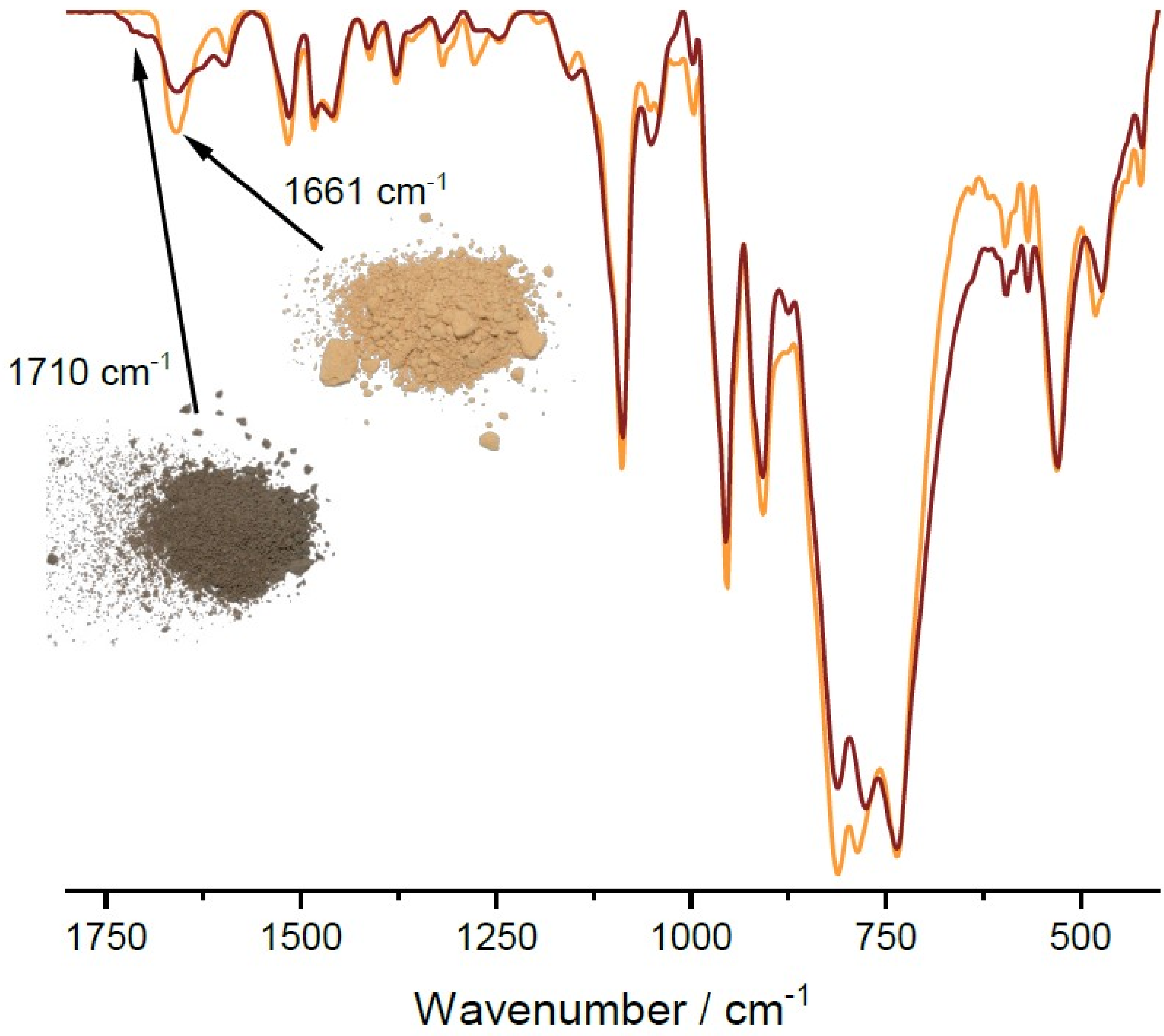

Figure S24: IR spectra of **FcPOM-2** before (orange) and after (brown) irradiation with λ_{ex} = 470 nm LED light source for 24 h.

3.4 EPR measurements - Radical generation from organic peroxides

Experiments were conducted with a constant peroxide concentration and varying cluster/ligand concentrations (ligand concentration was doubled to maintain the same concentration of photoactive ferrocene moieties). The PBN spin trap concentration was kept constant and in excess to ensure that the radical trapping rate depended only on the concentration of generated radicals. To investigate the irradiation of ferrocene species with peroxides and the PBN spin trap, a series of stock solutions were prepared to establish various measurement conditions. Two ferrocene stock solutions were created: one with 7.94 mg (1.25 μmol) of **FcPOM-1** in 0.25 mL of acetonitrile, and another with 11.4 mg (25.0 μmol) of PFc1 in 2.5 mL of acetonitrile. A PBN spin trap solution was also prepared by dissolving 11.08 mg (62.4 μmol) of PBN in 0.25 mL of acetonitrile. For experiments without peroxide dilution, the organic peroxides were used directly. For experiments involving diluted peroxides, 50 μL of the respective organic peroxide was diluted with 50 μL of either acetonitrile (for lower water

content) or water (for higher water content).

Table S3: Total peroxide and water content in measurement samples.

Peroxide	Stock solution	c(reaction)	H_2O content reaction
*t*BuOOH	7.35 M H_2O (71%)	1.25 M	4.50%
0.5 eq *t*BuOOH MeCN	3.675 M (1:1 / MeCN:H_2O)	0.625 M	2.25%
0.5 eq *t*BuOOH H_2O	3.675 M (H_2O)	0.625 M	13.0%

For all measurements, reactive solutions were prepared by combining 50 µL of the appropriate ferrocene stock solution (cluster or ligand), 50 µL of the PBN stock solution, and 20.4 µL of t-butyl hydroperoxide (*t*BuOOH). The mixture was then transferred to a 50 µL capillary and loaded into the EPR spectrometer. EPR spectra were recorded every 60 s for a total of 1800 s. For light-dependent experiments, a λ_{ex} = 470 ± 20 nm / 1 W LED was activated after 60 s (i.e., beginning with the second spectrum).

EPR spectrometer settings:

Sweep Range: 330-350 mT; Sweep Time: 20 s; Modulation: 0.2 mT; Modulation Frequency: 100 kHz; Microwave Power: 20 mW; Filter Parameter: 0.2; Filter type: DIG. For quantification purpose during irradiation-EPR measurements, a series of measurements with different TEMPOL concentrations between c = 0 mM and 1 mM were performed under same spectrometer conditions / setting as for the in-situ irradiation.

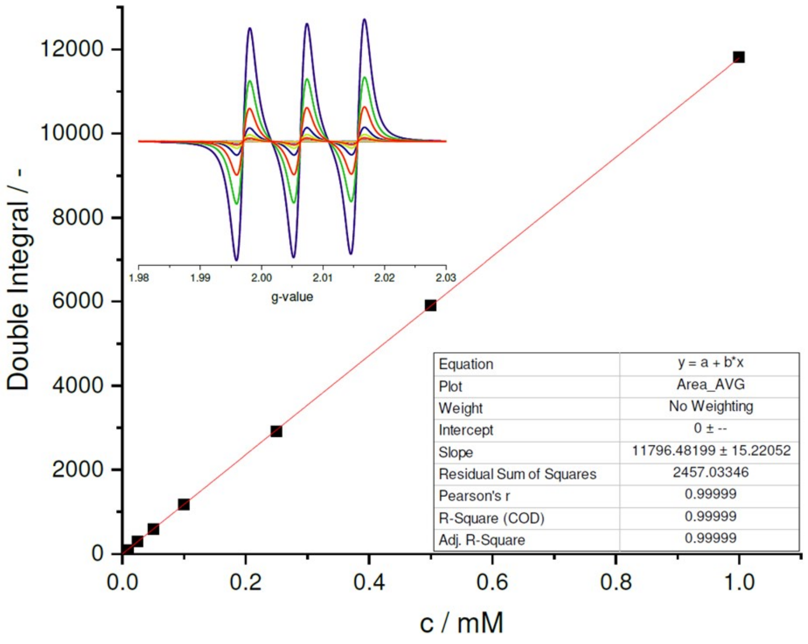

Figure S25: Calibration curve with TEMPOL c = 0 - 1 mM and linear fit.

3.5 EPR-determination of reaction rates

Alongside the general description of reaction and mechanism a concentration dependency was observed for the cluster as well as the ligand. This concentration-dependent behavior suggests that light-induced follow-up reactions, particularly involving the cluster, might degrade the trapped radicals to some extent. The reaction progress was fitted using either of four exponential functions, a biphasic exponential growth and decay function (Origin-ExpGrowDec), a standard exponential growth function (Origin-Exponential), an exponential decay function (Origin-ExpDec), or a two-component exponential growth function (Origin-ExpGro2), depending on the observed trend. Rate constants were derived from the fitted functions (Equations SI (6)-(12)). Assuming a similar initial radical generation and trapping mechanism for all reactions, the k_1 rate constants are comparable. The k_2 rate constants are more diffcult to compare due to their origin in different sub-reactions (e.g. photo-initiated follow-up reactions, radical breakdown reactions). Nevertheless, both k_1 and k_2 are summarized in ESI Tables S8 - S9.

k_1, associated with initial radical generation and trapping, tends to decrease with decreasing photocatalyst concentration, indicating a direct correlation between photocatalyst concentration and radical generation rate. k_2, arising from different reactions, is more complex. For high cluster concentrations, a decrease in signal intensity was observed after the initial rapid radical increase, suggesting a photoinduced follow-up reaction catalyzed by the cluster, rather than simple radical breakdown, as this was not observed for the ligand. At lower concentrations, the radical build up and degradation appear to reach an equilibrium (**FcPOM-1** / *t*BuOOH), with similar k_1 and k_2 values. The fact that this process required a two-component exponential suggests at least two, possibly coupled, processes.

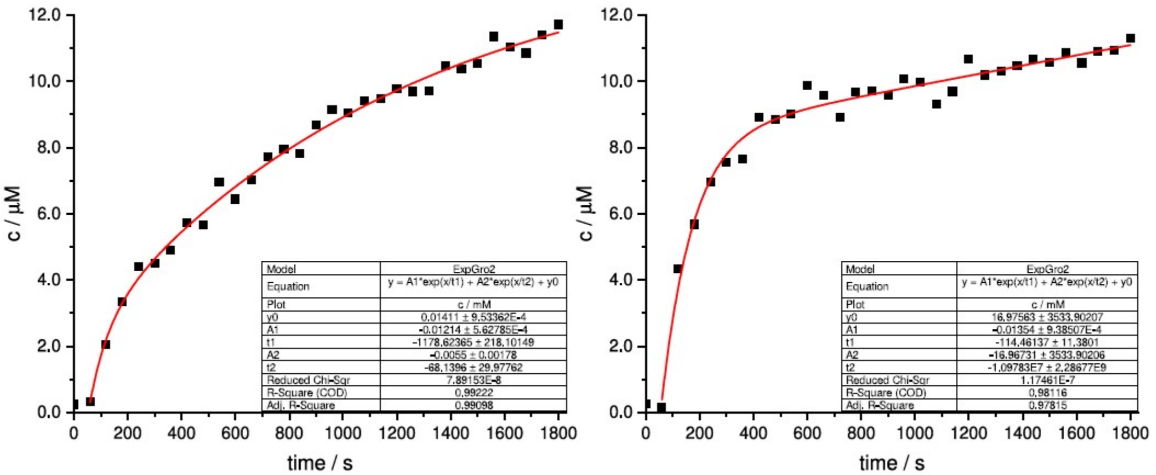

Figure S26: Time series concentration development of PBN trapped radicals:1.25 M *t*BuOOH and: Left: 0.2 mM **FcPOM-1** Right: 0.5 mM **FcPOM-1**.

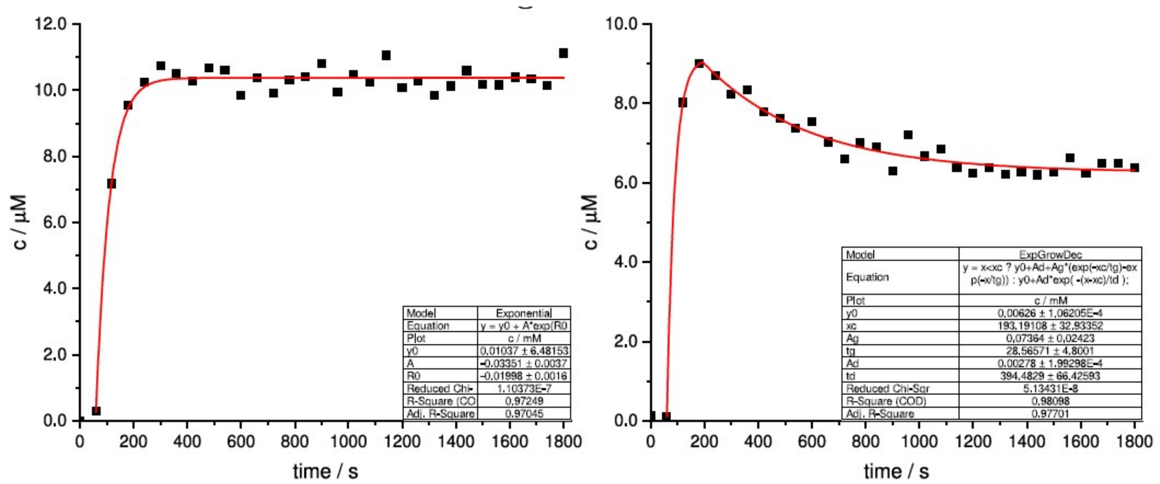

Figure S27: Time series concentration development of PBN trapped radicals:1.25 M *t*BuOOH and: Left: 1.0 mM **FcPOM-1** Right: 2.0 mM **FcPOM-1**.

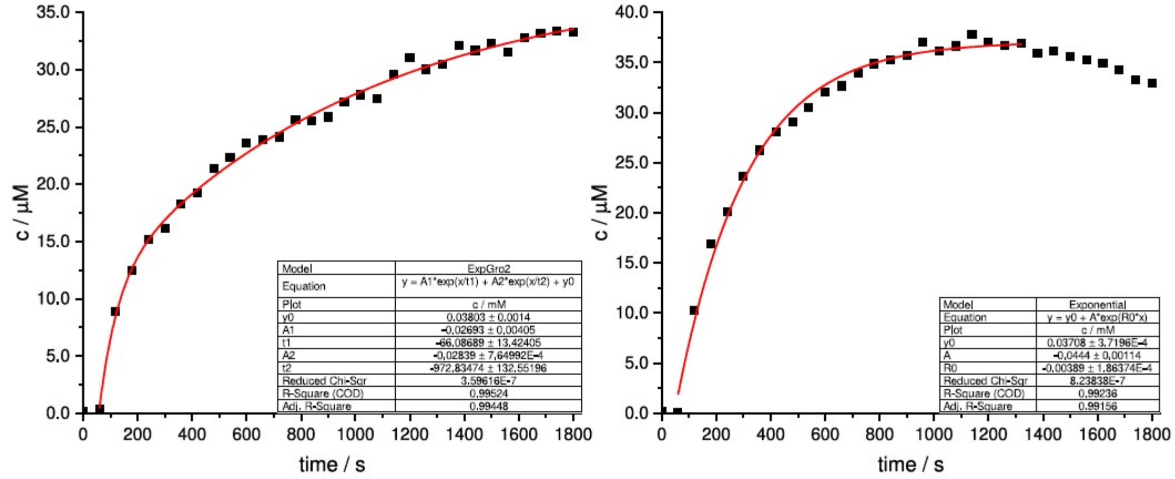

Figure S28: Time series concentration development of PBN trapped radicals: 2.0 mM **FcPOM-1** and Left: 0.625 M *t*BuOOH / MeCN Right: 0.625 M *t*BuOOH / H_2O.

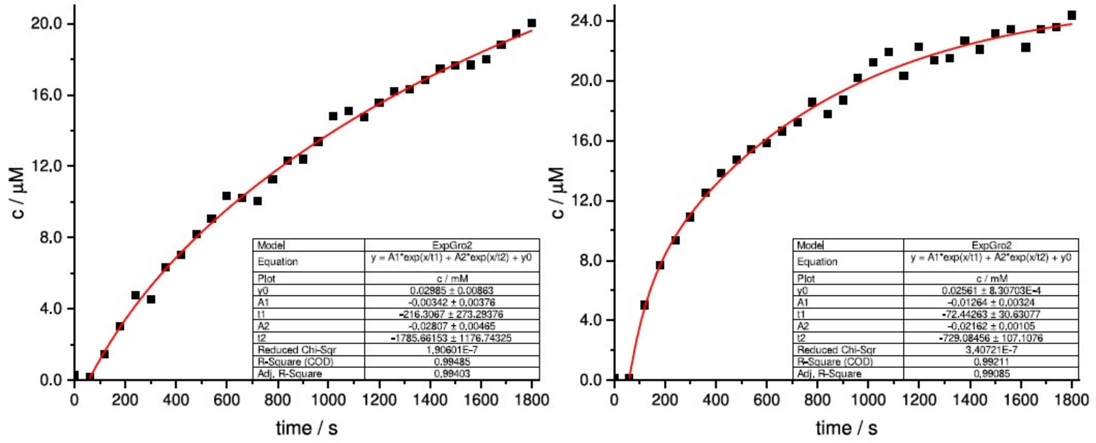

Figure S29: Time series concentration development of PBN trapped radicals, generated by 1.25 M *t*BuOOH and: Left: 0.4 mM PFc1 Right: 1.0 mM PFc1.

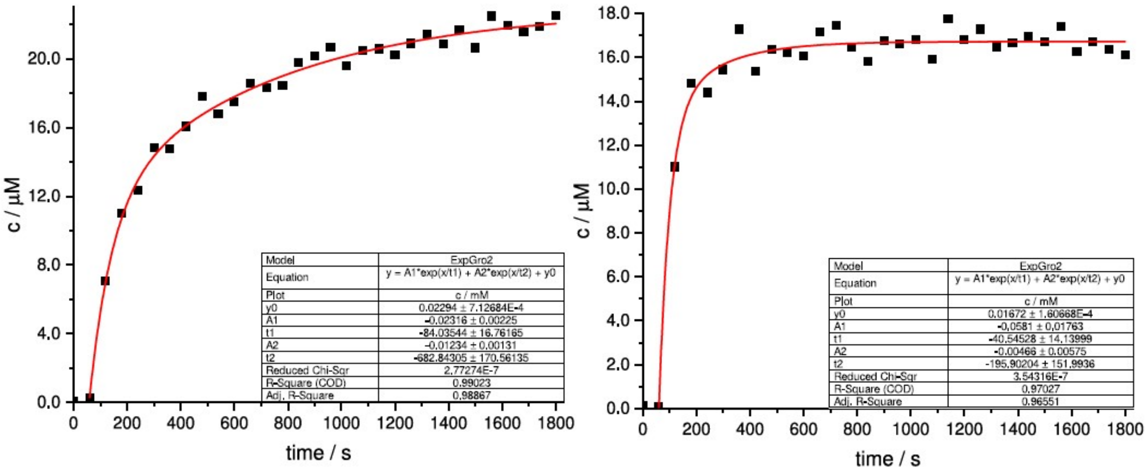

Figure S30: Time series concentration development of PBN trapped radicals, generated by1.25 M *t*BuOOH and: Left: 2.0 mM PFc1 Right: 4.0 mM PFc1.

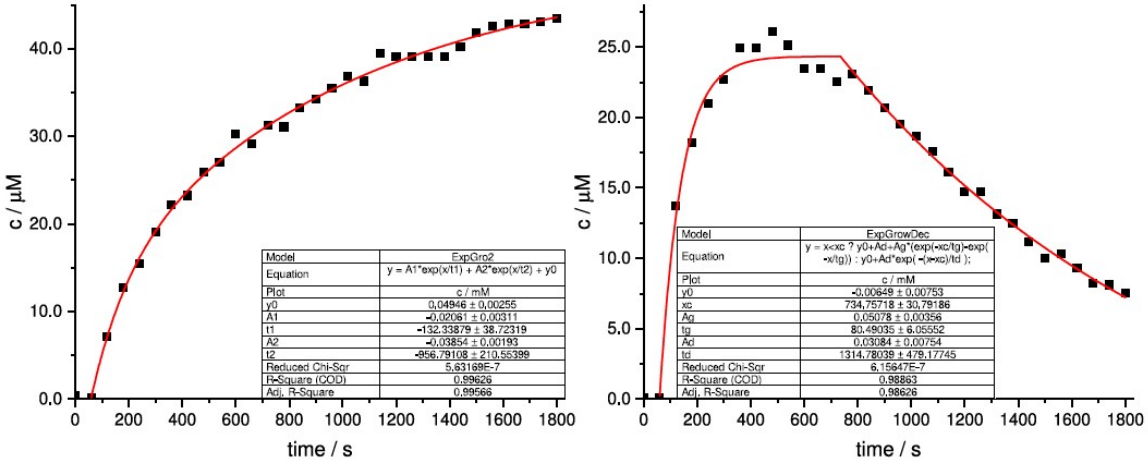

Figure S31: Time series concentration development of PBN trapped radicals, generated by4.0 mM PFc1 and Left: 0.625 M *t*BuOOH / MeCN Right: 0.625 M *t*BuOOH / H_2O.

3.6 EPR Simulations of Peroxide Degradation

FcPOM-1 with tert -Butyl hydroperoxide

Table S6: Simulated EPR parameters: Varying **FcPOM-1** concentrations (c / mM) with 1.25 M *t*BuOOH respectively 0.625 mM *t*BuOOH diluted with *MeCN or †H_2O.

c / mM	Time / s	g /-	A_N / mT	A_H / mT	Lwpp (gaussian)	Lwpp (lorentzian)	weight	RMSD
0.2 mM	1800	2.0059	1.422	0.243	0.156	0.156	-	0.0242
0.5 mM	1800	2.0059	1.403	0.219	0.134	0.173	-	0.0196
1.0 mM	1800	2.0059	1.394	0.212	0.147	0.155	-	0.0217
2.0 mM	1800	2.0059	1.374	0.159	0.078	0.198	-	0.0285
2.0 mM*	1800	2.0059	1.427	0.237	0.171	0.161	-	0.0225
2.0 mM†	120	2.0059	1.403	-	0.256	0.183	-	0.0386
	1800	2.0059	1.427	0.282	0.148	0.074	0.60	0.0380
		2.0059	1.378	-	0.166	0.201	0.35	

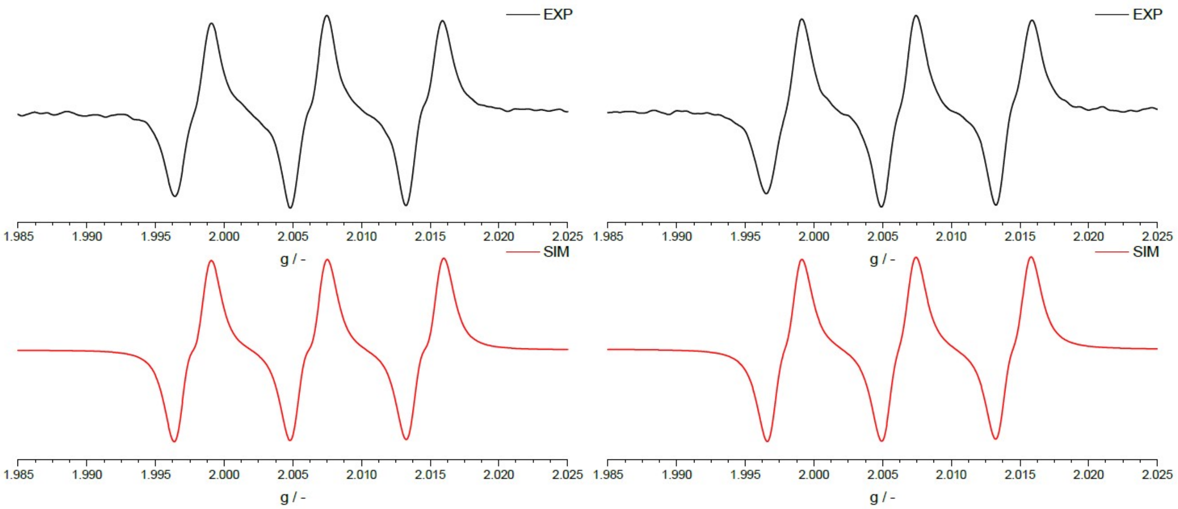

Figure S32: Simulation and experimental EPR spectra: Left: 0.2 mM **FcPOM-1**, 1.25 M *t*BuOOH and Right: 0.5 mM **FcPOM-1**, 1.25 M *t*BuOOH.

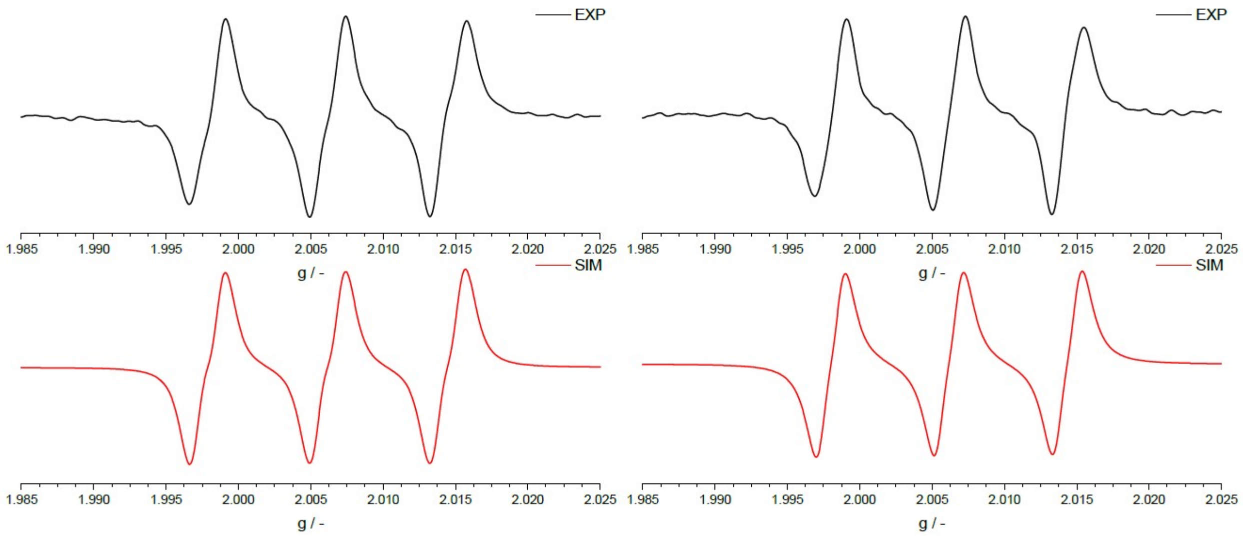

Figure S33: Simulation and experimental EPR spectra: Left: 1.0 mM **FcPOM-1**, 1.25 M *t*BuOOH and Right: 2.0 mM **FcPOM-1**, 1.25 M *t*BuOOH.

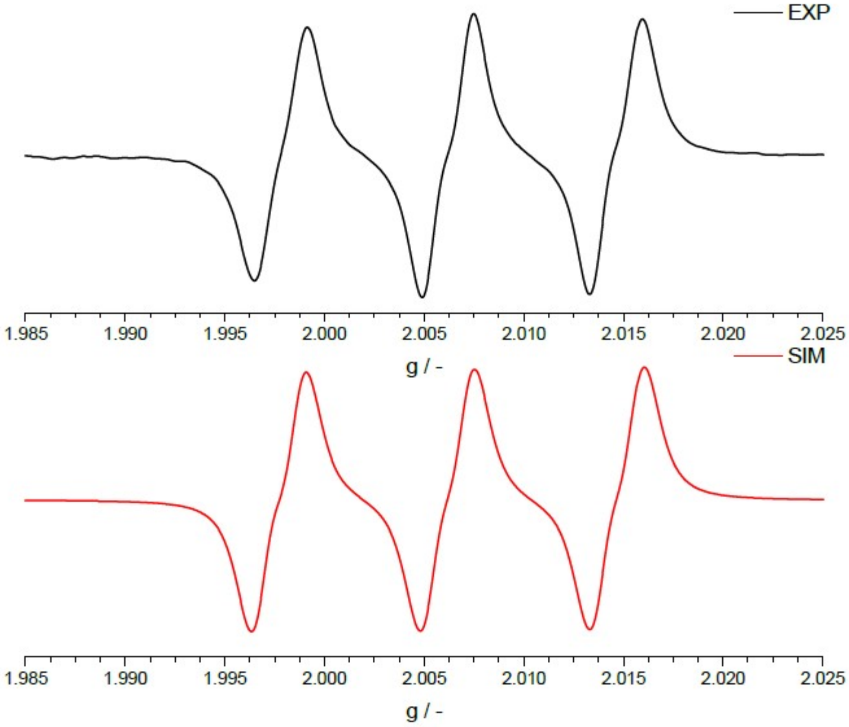

Figure S34: Simulation and experimental EPR spectra: 2.0 mM **FcPOM-1**, 0.625 M *t*BuOOH.

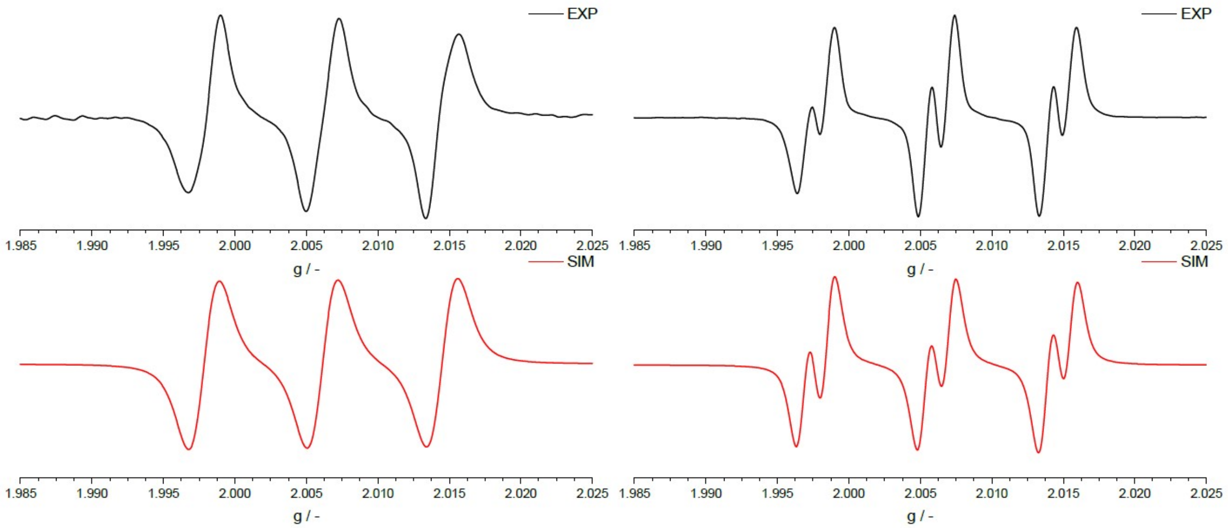

Figure S35: Simulation and experimental EPR spectra: 2.0 mM **FcPOM-1**, 0.625 M *t*BuOOH. / H_2O at Left: 120 s Right: 1800 s.

PFc1 with tert-Butyl hydroperoxide

Table S7: Simulated EPR parameters: Varying PFc1 concentrations (c / mM) with 1.25 M *t*BuOOH respectively 0.625 mM *t*BuOOH diluted with ∗MeCN or †H2O

c / mM	Time / s	g / -	A_N / mT	A_H / mT	Lwpp (gaussian)	Lwpp (Lorentzian)	weight	RMSD
0.4 mM	1800	2.0059	1.413	0.226	0.156	0.159	-	0.0242
1.0 mM	1800	2.0059	1.403	0.219	0.132	0.171	-	0.0196
2.0 mM	1800	2.0059	1.398	0.207	0.132	0.163	-	0.0217
4.0 mM	1800	2.0059	1.386	0.189	0.072	0.195	-	0.0285
4.0 mM∗	1800	2.0060	1.411	0.219	0.149	0.160	-	0.0225
4.0 mM†	120	2.0059	1.384	-	0.281	0.119	-	0.0271
4.0 mM†	1800	2.0060	1.409	0.201	0.099	0.102	0.58	0.0324
4.0 mM†	1800	2.0059	1.480	0.321	0.155	0.073	0.24	0.0324

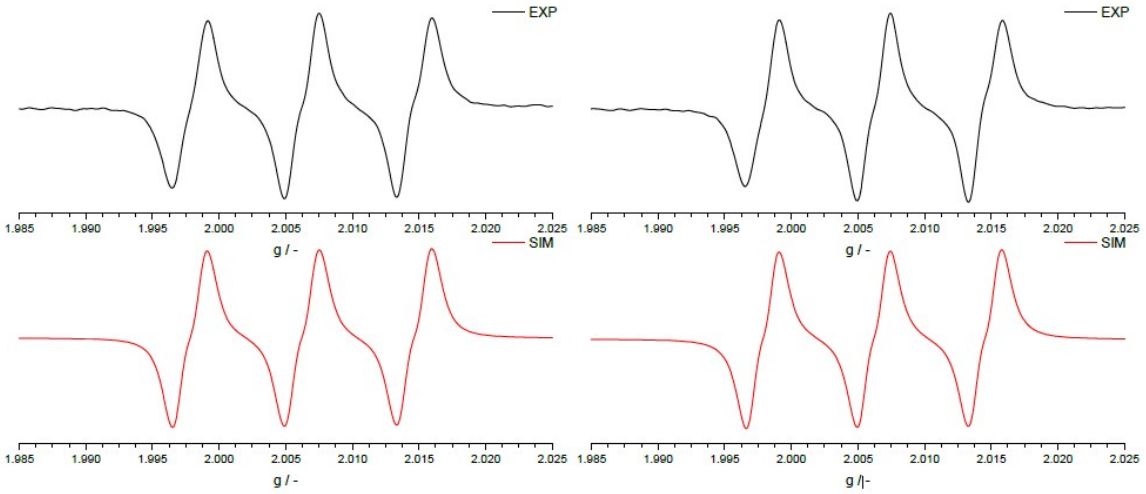

Figure S36: Simulation and experimental EPR spectra: Left: 0.4 mM PFc1, 1.25 M *t*BuOOH and Right: 1.0 mM PFc1, 1.25 M *t*BuOOH.

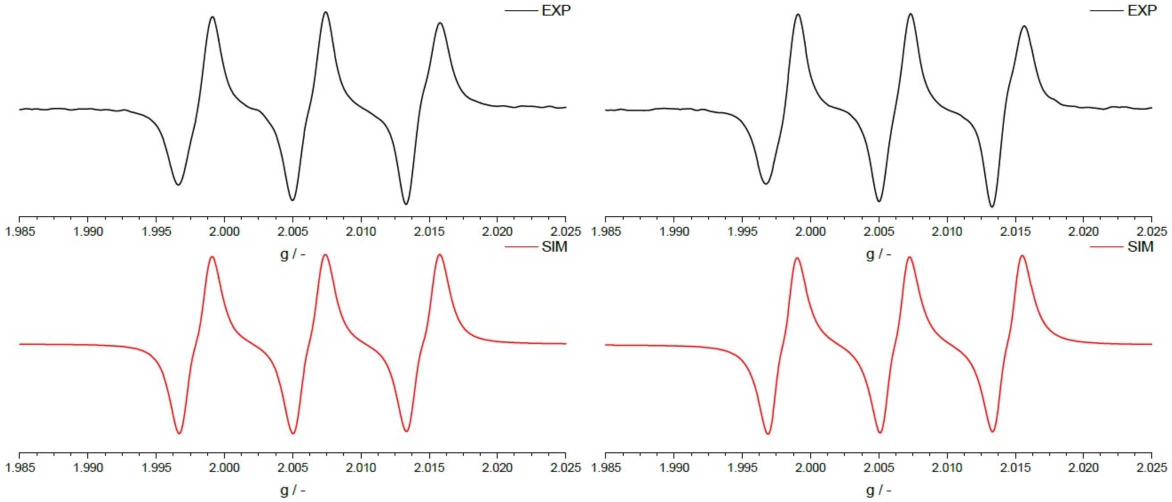

Figure S37: Simulation and experimental EPR spectra: Left: 2.0 mM PFc1, 1.25 M *t*BuOOH and Right: 4.0 mM PFc1, 1.25 M *t*BuOOH.

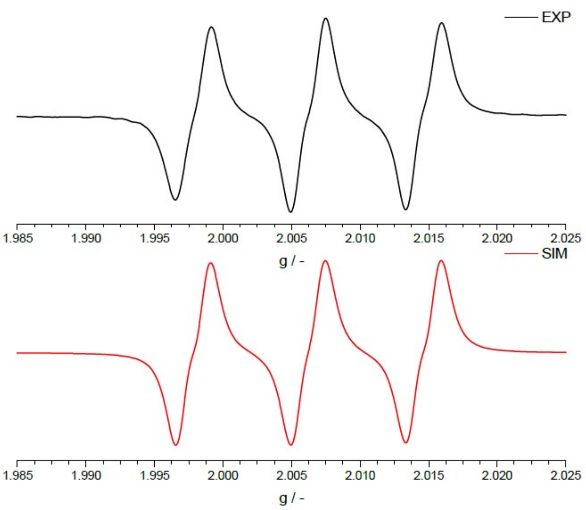

Figure S38: Simulation and experimental EPR spectra: 4.0 mM PFc1, 0.625 M *t*BuOOH / MeCN.

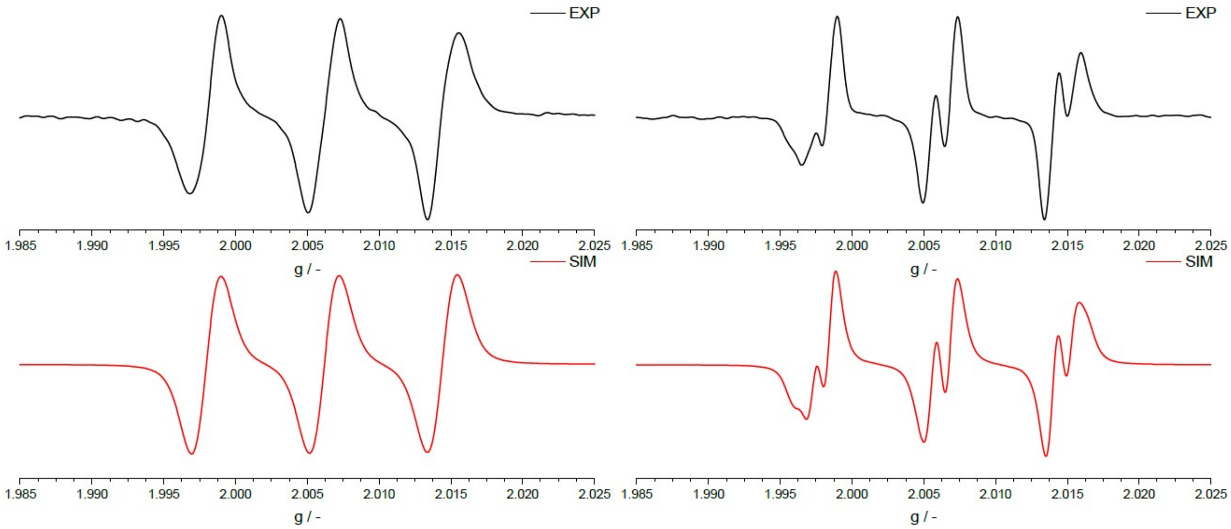

Figure S39: Simulation and experimental EPR spectra: 4.0 mM PFc1, 0.625 M *t*BuOOH / H_2O at Left: 480 s Right: 1800

Exponential fitting functions used

Biphasic exponential growth and decay function: Origin-ExpGrowDec

$$y = \begin{cases} y_0 + A_d + A_g \left(e^{-\frac{x_c}{t_g}} - e^{-\frac{x}{t_g}} \right) & \text{for } x \leq x_c \\ y_0 + A_d \cdot e^{-\frac{(x-x_c)}{t_d}} & \text{for } x > x_c \end{cases}$$

$$\begin{aligned} y(x \leq x_c) &= y_0 + A_d + A_g \left(e^{-\frac{x_c}{t_g}} - e^{-\frac{x}{t_g}} \right) \\ &= \underbrace{y_0 + A_d + A_g \cdot e^{-\frac{x_c}{t_g}}}_{=y_0} \underbrace{-A_g}_{=A} \cdot \underbrace{e^{-\frac{x}{t_g}}}_{=e^{-k\cdot x}} \\ &= y_0 + A \cdot e^{-k\cdot x} \end{aligned}$$

$$\begin{aligned} y(x > x_c) &= y_0 + A_d \cdot \underbrace{e^{-\frac{(x-x_c)}{t_d}}}_{=e^{-k\cdot(x-x_c)}} \\ &= y_0 + A_d \cdot e^{-k\cdot(x-x_c)} \end{aligned}$$

Exponential growth function: Origin-Exponential

$$\begin{aligned} y &= y_0 + A \cdot \underbrace{e^{R_0 \cdot x}}_{=e^{-k\cdot x}} \\ &= y_0 + A \cdot e^{-k\cdot x} \end{aligned}$$

Exponential decay function: Origin-ExpDec

$$\begin{aligned} y &= y_0 + A \cdot \underbrace{e^{-\frac{x}{t}}}_{=e^{-k\cdot x}} \\ &= y_0 + A \cdot e^{-k\cdot x} \end{aligned}$$

Biphasic exponential growth function: Origin-ExpGro2

$$\begin{aligned} y &= y_0 \cdot \left[A_1 \cdot e^{\frac{x}{t_1}} + A_2 \cdot e^{\frac{x}{t_2}} \right] \\ &= \underbrace{y_0 \cdot A_1}_{=A_1} \cdot \underbrace{e^{\frac{x}{t_1}}}_{=e^{-k_1\cdot x}} + \underbrace{y_0 \cdot A_2}_{=A_2} \cdot \underbrace{e^{\frac{x}{t_2}}}_{=e^{-k_2\cdot x}} \end{aligned}$$

Table S8: Fitted rate constants for radical generation with *t*BuOOH at different **FcPOM-1** concentrations or fixed **FcPOM-1** concentration (c = 2.0 mM) and *t*BuOOH (0.625 mM) diluted with *MeCN or †H_2O.

Measurement	c / mM	Fit	k_1 / s^{-1}	k_2 / s^{-1}
**FcPOM-1** / *t*BuOOH	2.0	ExpGrowDec	$3.50 \cdot 10^{-2} \pm 5.88 \cdot 10^{-3}$	$2.53 \cdot 10^{-3} \pm 4.26 \cdot 10^{-4}$
	1.0	Exponential	$2.00 \cdot 10^{-2} \pm 1.63 \cdot 10^{-3}$	-
	0.5	ExpGro2	$8.74 \cdot 10^{-3} \pm 8.64 \cdot 10^{-4}$	$9.09 \cdot 10^{-8} \pm 1.89 \cdot 10^{-5}$
	0.2	ExpGro2	$8.48 \cdot 10^{-4} \pm 1.57 \cdot 10^{-4}$	$1.47 \cdot 10^{-2} \pm 6.47 \cdot 10^{-3}$
	2.0*	ExpGro2	$1.51 \cdot 10^{-2} \pm 3.10 \cdot 10^{-3}$	$1.00 \cdot 10^{-3} \pm 1.40 \cdot 10^{-4}$
	2.0†	Exponential	$3.89 \cdot 10^{-3} \pm 1.86 \cdot 10^{-7}$	-

Table S9: Fitted rate constants for radical generation with *t*BuOOH at different PFc1 concentrations or fixed PFc1 concentration (c = 4.0 mM) and *t*BuOOH (0.625 mM) diluted with *MeCN or †H_2O.

Measurement	c / mM	Fit	k_1 / $\frac{1}{s}$	k_2 / $\frac{1}{s}$
**PFc1** / *t*BuOOH	4.0	ExpGro2	$2.47 \cdot 10^{-2} \pm 8.60 \cdot 10^{-3}$	$5.00 \cdot 10^{-3} \pm 3.80 \cdot 10^{-3}$
	2.0	ExpGro2	$1.20 \cdot 10^{-2} \pm 2.40 \cdot 10^{-3}$	$1.50 \cdot 10^{-3} \pm 4.00 \cdot 10^{-4}$
	1.0	ExpGro2	$1.38 \cdot 10^{-2} \pm 5.80 \cdot 10^{-3}$	$1.40 \cdot 10^{-3} \pm 2.00 \cdot 10^{-4}$
	0.4	ExpGro2	$4.60 \cdot 10^{-3} \pm 6.00 \cdot 10^{-3}$	$5.60 \cdot 10^{-4} \pm 3.70 \cdot 10^{-4}$
	4.0*	ExpGro2	$7.56 \cdot 10^{-3} \pm 2.21 \cdot 10^{-3}$	$1.05 \cdot 10^{-3} \pm 2.30 \cdot 10^{-4}$
	4.0†	ExpGrowDec	$1.24 \cdot 10^{-2} \pm 9.34 \cdot 10^{-4}$	$7.61 \cdot 10^{-4} \pm 2.77 \cdot 10^{-4}$

4 Transient absorption spectroscopy

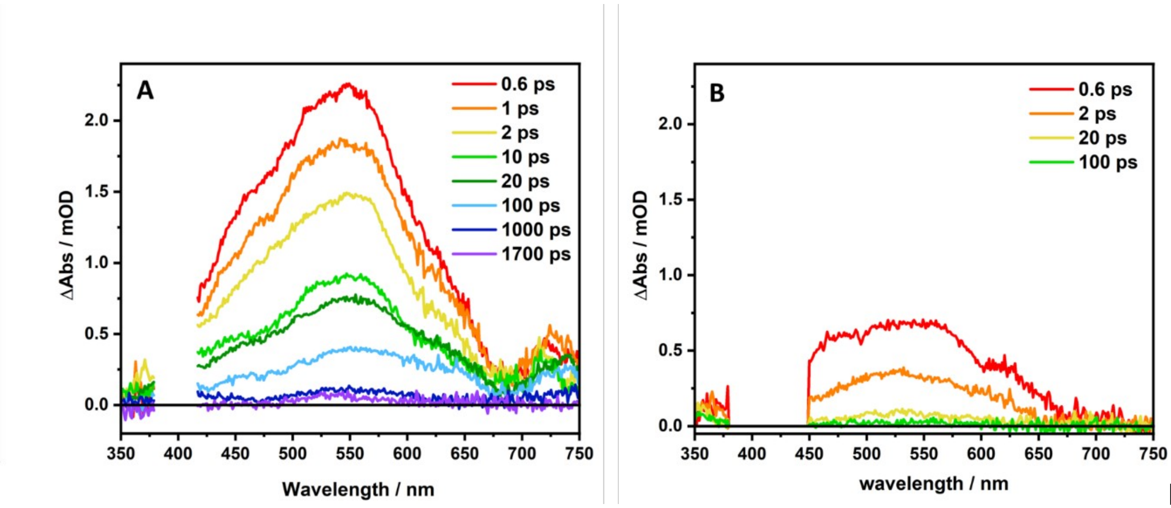

Figure S40: Transient absorption UV-Vis spectra at selected delay times for **FcPOM-1** (A) and **PFc1** (B), pumped at 403 nm.

5 EPR LED Mount

In-situ irradiation for EPR measurements were achieved by a passively cooled P = 1 W λ = 470 ± 20 nm LED (Spot MinoStar 1 W blue, 15°, driven with a I = 350 mA constant current LED driver), mounted in front of the EPR. To mount the LED at the front plate a screwable mount, fitting with the thread provided by the EPR (22x1 mm), was designed. The LED was fitted into the mount and was hold in place with a small PTFE screw (in this case M3 screw but is freely selectable) without damaging the LED body. Side view as well as angled bottom and top views of the designed mount are shown below. A quick test showed, that the irradiation region within the resonator extends well outside the active EPR measurement region. This means that at least the whole area, detected by the EPR is irradiated.

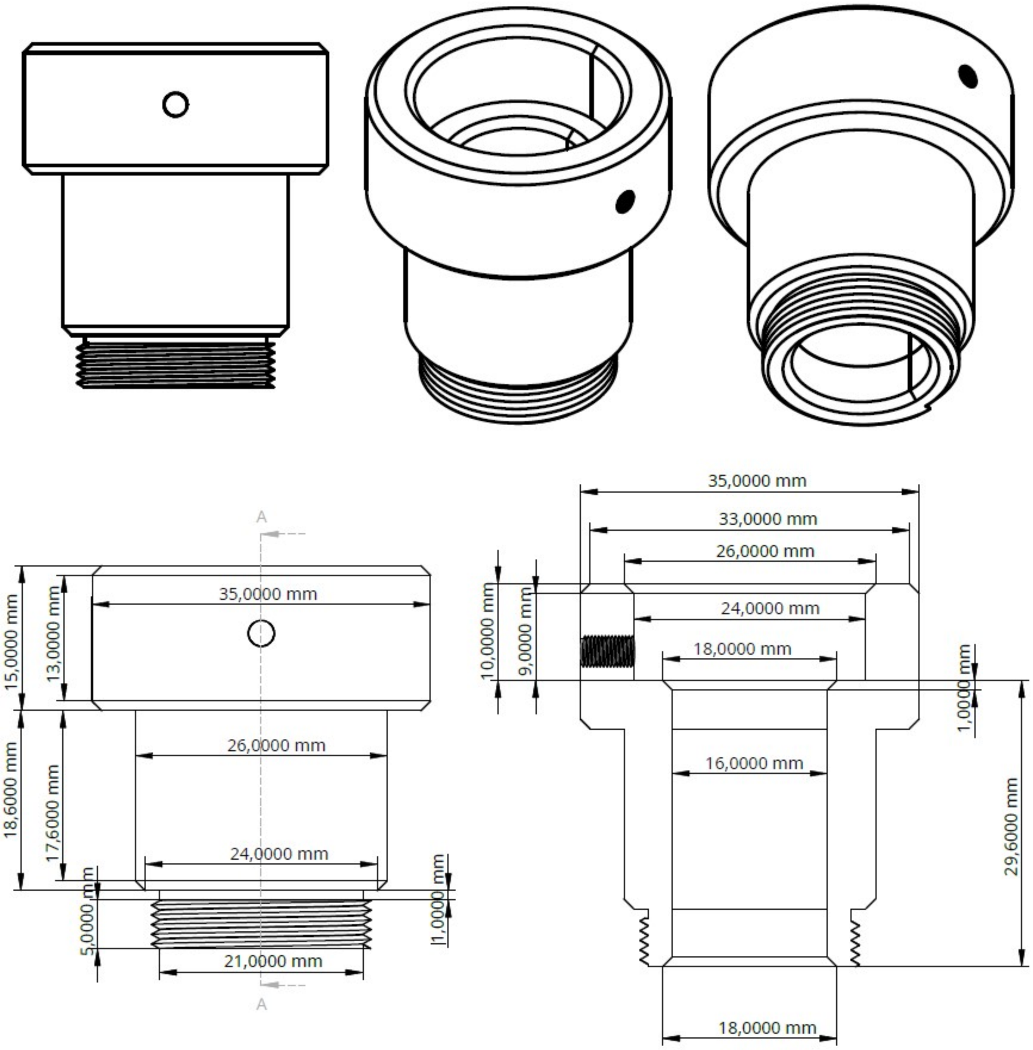

Figure S41: Top: Side and angled top as well as bottom view of the LED mount for Magnettech MS5000; bottom: Design schematic including dimensions of the LED mount for Magnettech MS5000.[8]

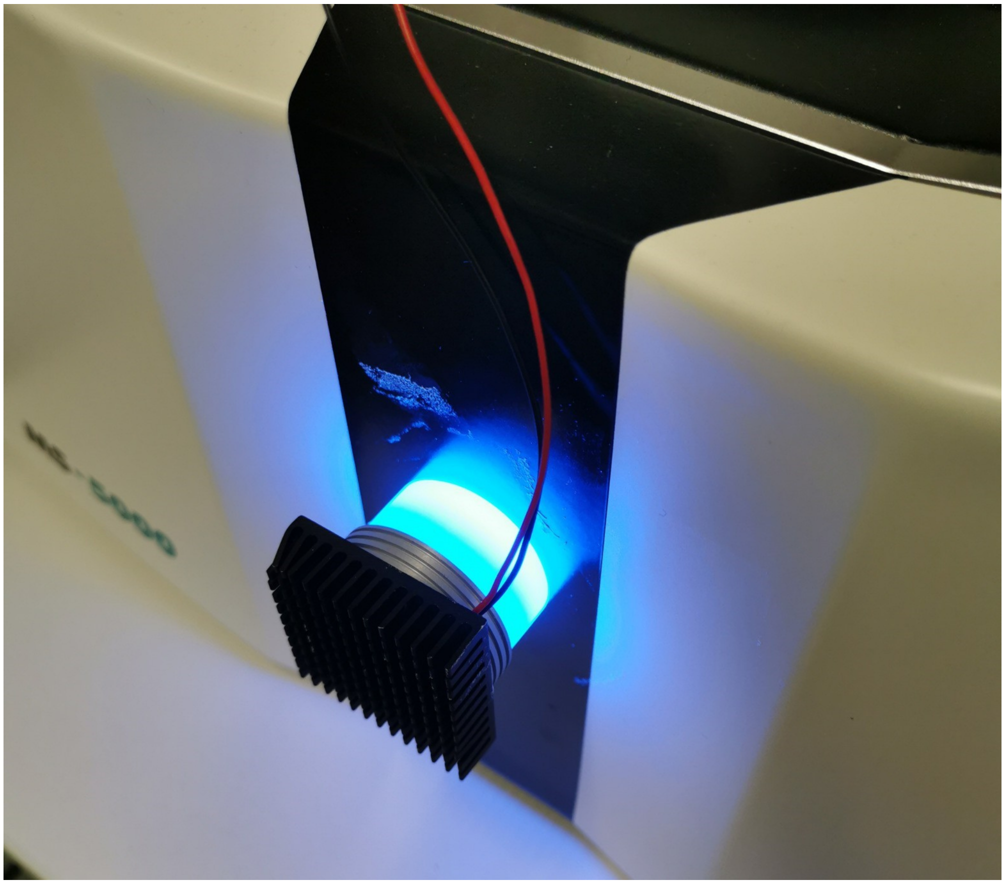

Figure S42: 470 nm LED mounted at the MS5000 front-plate with the custom-designed LED mount.

6 Quantum chemistry

All quantum chemical calculations were performed using the Gaussian 16(C.02)[9] software to investigate structural and electronic properties of the **Fc-POM-1** dyad. Several electronic configurations were considered and fully optimized by means of density functional theory (DFT), namely the closed-shell singlet ground state, **^{1}[Fc-POM-1]**, the (opened-shell and charge-separated) triplet species (**^{3}[Fc-POM-1]**) as well as the singly oxidized dyad within doublet multiplicity (**^{2}[Fc-POM-1]**). The TPSSh[10–12] hybrid[13,14] functional – featuring 15% of exact exchange – was utilized in combination with the all-electron def2-SVP[15] basis set and the respective pseudo potential in case of the tungsten atoms of the Dawson POM. All structures were fully optimized while considering implicit solvent effects (acetonitrile: ε = 35.688) using the solute electron density (SMD) variant of the integral equation formalism of the polarizable continuum model (equilibrium procedure).[16,17] All calculations were performed including D3 dispersion correction with Becke-Johnson damping.[18,19] Subsequently, a vibrational analysis was carried out for each optimized structure to verify that a (local) minimum on the $3N$-6-dimensional potential energy (hyper-)surface (PES) was obtained.

Subsequently, TDDFT simulations were performed to elucidate the electronic transitions involved in the Franck-Condon photophysics of **^{1}[Fc-POM-1]** based on the lowest 300 singlet-to-singlet transitions. To this aim the same computational setup was applied as in the previous ground state calculations, while the non-equilibrium model was utilized to account for implicit solvent effects.

In addition, the transient absorption (TA) signal stemming from the charge-separated triplet species, **^{3}[Fc-POM-1]**, was simulated. The TA spectrum was calculated as difference spectrum based on the excited state absorption (ESA) originating from the 300 lowest energy triplet-to-triplet transitions with the fully relaxed T_1 equilibrium and the ground state bleach (GSB) as modeled by the singlet-to-singlet transitions within the Franck-Condon point. A relative population of 1:1 was assumed for the excited state vs. the ground state species.[20–23]

All calculated equilibrium structures as well as high resolution images (charge density differences and spin densities) are available from the free online repository Zenodo[24].

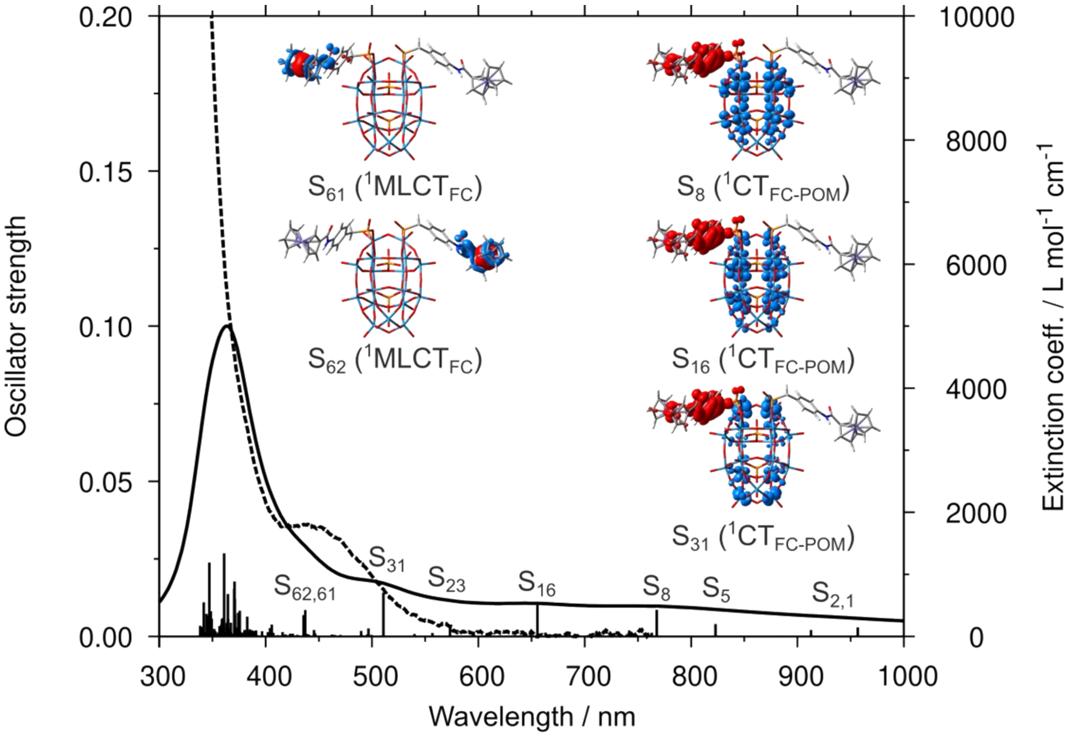

Figure S43: TDDFT-simulated (solid black) and experimental (black dashed) UV-Vis absorption spectra of **Fc-POM-1** in acetonitrile. Key electronic transitions are indicated and visualized by means of charge density difference plots; charge transfer occurs from red to blue.

Table S10: TDDFT-calculated electronic (singlet-to-singlet) transitions involved in the electronic absorption spectrum of **Fc-POM-1** in acetonitrile; electronic properties such as electronic characters, excitation energies, excitation wavelengths and oscillator strengths are indicated.

Excitation $S_0 \rightarrow S_i$	Character	ΔE / eV	λ / nm	f
S_1	$^1CT_{Fc\text{-}POM}$	1.30	957	0.0029
S_2	$^1CT_{Fc\text{-}POM}$	1.36	913	0.0021
S_5	$^1CT_{L\text{-}POM}$	1.51	823	0.0040
S_8	$^1CT_{L\text{-}POM}$	1.62	768	0.0086
S_{16}	$^1CT_{L\text{-}POM}$	1.89	656	0.0101
S_{23}	$^1CT_{Fc\text{-}POM}$	2.16	573	0.0037
S_{31}	$^1CT_{L\text{-}POM}$	2.43	511	0.0141
S_{61}	$^1MLCT_{Fc}$	2.84	437	0.0085
S_{62}	$^1MLCT_{Fc}$	2.85	436	0.0069
S_{90}	$^1MLCT_{Fc}$	3.06	406	0.0038
S_{94}	$^1MLCT_{Fc}$	3.07	404	0.0027
S_{144}	$^1CT_{L\text{-}POM}$	3.24	383	0.0064

Table S11: Electronic (singlet-to-singlet) transitions within the relaxed singlet ground state as visualized by means of charge density difference plots; charge transfer occurs from red to blue.

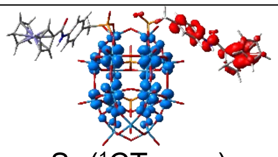 S_1 ($^1CT_{Fc\text{-}POM}$)	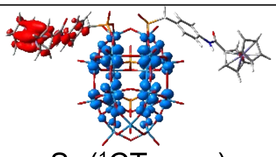 S_2 ($^1CT_{Fc\text{-}POM}$)	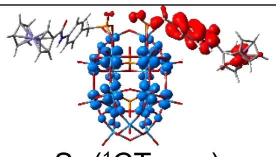 S_5 ($^1CT_{L\text{-}POM}$)	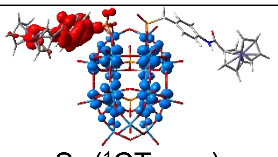 S_8 ($^1CT_{L\text{-}POM}$)
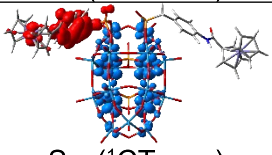 S_{16} ($^1CT_{L\text{-}POM}$)	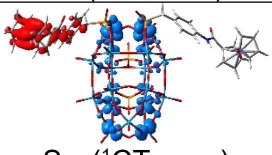 S_{23} ($^1CT_{Fc\text{-}POM}$)	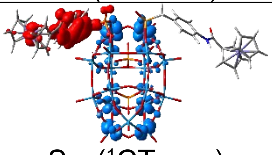 S_{31} ($^1CT_{L\text{-}POM}$)	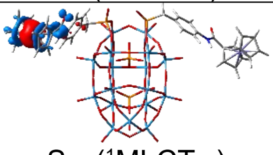 S_{61} ($^1MLCT_{Fc}$)
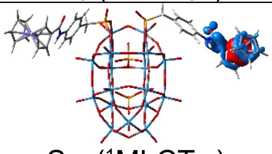 S_{62} ($^1MLCT_{Fc}$)	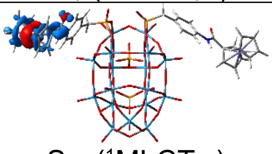 S_{90} ($^1MLCT_{Fc}$)	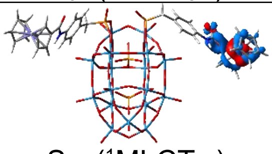 S_{94} ($^1MLCT_{Fc}$)	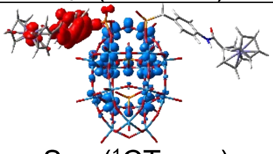 S_{144} ($^1CT_{L\text{-}POM}$)

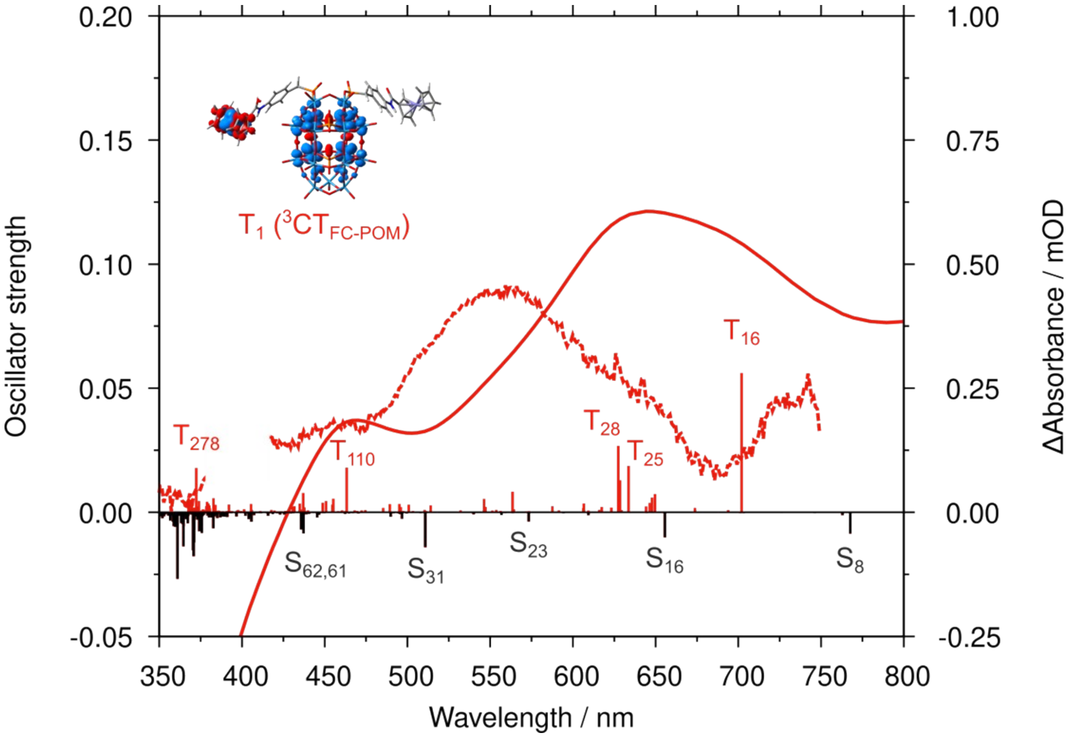

Figure S44: TDDFT-simulated (solid red) and experimental (dashed red) UV-Vis transient absorption spectra of **Fc-POM-1** in acetonitrile. Key electronic transitions involved in the excited state absorption are indicated, while the nature of the charge-separated triplet ground state (T_1) is visualized by means of its spin density.

Table S12: TDDFT-calculated electronic (triplet-to-triplet) transitions involved in the excited state absorption of the transient absorption spectrum of **Fc-POM-1** in acetonitrile; electronic properties such as electronic characters, excitation energies, excitation wavelengths, oscillator strengths and spin-contaminations are indicated.

Excitation $T_1 \rightarrow T_i$	Character	ΔE / eV	λ / nm	f	$\langle \hat{S}^2 \rangle$
T_{13}	$^3LMCT_{POM}$	1.41	880	0.0465	2.04
T_{14}	$^3LMCT_{POM}$	1.45	855	0.0160	2.04
T_{16}	$^3LMCT_{POM}$	1.77	702	0.0561	2.04
T_{25}	$^3LMCT_{POM}$	1.96	634	0.0187	2.05
T_{28}	$^3LMCT_{POM/L\text{-}FC}$	1.98	627	0.0268	2.49
T_{110}	$^3LMCT_{L\text{-}FC}$	2.68	463	0.0180	2.91
T_{278}	$^3LMCT_{POM}$	3.33	372	0.0179	2.95

Table S13: Electronic (triplet-to-triplet) transitions within the relaxed triplet ground state (T_1, see spin density) as visualized by means of charge density difference plots; charge transfer occurs from red to blue.

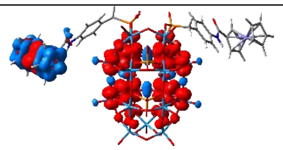<br>T_1 ($^3CT_{Fc\text{-}POM}$)	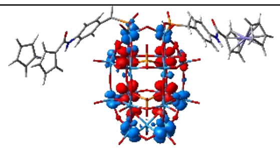<br>T_{13} ($^3LMCT_{POM}$)	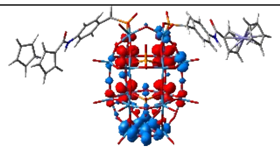<br>T_{14} ($^3LMCT_{POM}$)	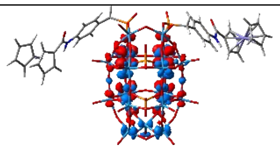<br>T_{16} ($^3LMCT_{POM}$)
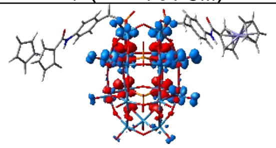<br>T_{25} ($^3LMCT_{POM}$)	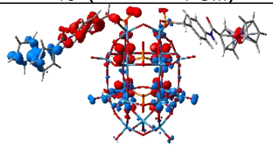<br>T_{28} ($^3LMCT_{POM/L\text{-}FC}$)	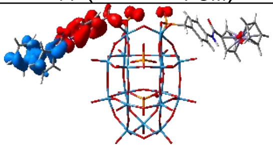<br>T_{110} ($^3LMCT_{L\text{-}FC}$)	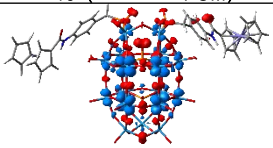<br>T_{278} ($^3LMCT_{POM}$)

7 References

1 R. Siebert, D. Akimov, M. Schmitt, A. Winter, U. S. Schubert, B. Dietzek and J. Popp, *ChemPhysChem*, 2009, **10**, 910–919.
2 M. Karnahl, C. Kuhnt, F. Ma, A. Yartsev, M. Schmitt, B. Dietzek, S. Rau and J. Popp, *ChemPhysChem*, 2011, **12**, 2101–2109.
3 A. L. Dobryakov, S. A. Kovalenko and N. P. Ernsting, *Journal of Chemical Physics*, DOI:10.1063/1.1948383/900640.
4 C. Müller, T. Pascher, A. Eriksson, P. Chabera and J. Uhlig, *J. Phys. Chem. A*, 2022, **126**, 4087–4099.
5 A. P. Ginsberg, *Inorganic Syntheses. Vol. 27; Ginsberg, A. P., Ed.; John Wiley & Sons, Inc.: New York; Chichester, .*
6 S. Knoll and C. Streb, *Inorg. Chem.*, 2023, **62**, 1218–1225.
7 H. Rigneault, N. G. Kumar, R. Cossart, D. Septier, G. Brévalle-Waslilewki, A. Kudlinski and A. Kaszas, *Focus on Microscopy*, 2025, 255.
8 FreeCAD V0.19. https://www.freecad.org/index.php?lang=de.
9 Citation | Gaussian.com, https://gaussian.com/citation/, (accessed 30 January 2026).
10 V. N. Staroverov, G. E. Scuseria, J. Tao and J. P. Perdew, *J. Chem. Phys.*, 2004, **121**, 11507–11507.
11 V. N. Staroverov, G. E. Scuseria, J. Tao and J. P. Perdew, *J. Chem. Phys.*, 2003, **119**, 12129–12137.
12 J. Tao, J. P. Perdew, V. N. Staroverov and G. E. Scuseria, *Phys. Rev. Lett.*, 2003, **91**, 146401.
13 C. Lee, W. Yang and R. G. Parr, *Phys. Rev. B*, 1988, **37**, 785.
14 A. D. Becke, *J. Chem. Phys.*, 1993, **98**, 5648–5652.
15 F. Weigend and R. Ahlrichs, *Physical Chemistry Chemical Physics*, 2005, **7**, 3297–3305.
16 G. D. Scholes, C. Curutchet, B. Mennucci, R. Cammi and J. Tomasi, *Journal of Physical Chemistry B*, 2007, **111**, 6978–6982.
17 A. V. Marenich, C. J. Cramer and D. G. Truhlar, *Journal of Physical Chemistry B*, 2009, **113**, 6378–6396.
18 A look at the density functional theory zoo with the advanced GMTKN55 database for general main group thermochemistry, kinetics and noncovalent intera ... - Physical Chemistry Chemical Physics (RSC Publishing) DOI:10.1039/C7CP04913G, https://pubs.rsc.org/en/content/articlehtml/2017/cp/c7cp04913g, (accessed 30 January 2026).
19 S. Grimme, S. Ehrlich and L. Goerigk, *J. Comput. Chem.*, 2011, **32**, 1456–1465.
20 C. Wegeberg, D. Häussinger, S. Kupfer and O. S. Wenger, *J. Am. Chem. Soc.*, 2024, **146**, 4605–4619.
21 G. Yang, L. Blechschmidt, L. Zedler, C. Zens, K. Witas, M. Schmidt, B. Esser, S. Rau, G. E. Shillito, B. Dietzek-Ivanšić and S. Kupfer, *Chemistry - A European Journal*, 2025, **31**, e202404671.
22 G. E. Shillito, T. B. J. Hall, D. Preston, P. Traber, L. Wu, K. E. A. Reynolds, R. Horvath, X. Z. Sun, N. T. Lucas, J. D. Crowley, M. W. George, S. Kupfer and K. C. Gordon, *J. Am. Chem. Soc.*, 2018, **140**, 4534–4542.
23 L. Zedler, S. Kupfer, H. Schmidt and B. Dietzek-Ivanšić, *Chemistry - A European Journal*, 2024, **30**, e202303079.
24 S. Kupfer, DOI:10.5281/ZENODO.18835408.
